# Synthetic Derivatives of Natural *ent*-Kaurane Atractyligenin Disclose Anticancer Properties in Colon Cancer Cells, Triggering Apoptotic Cell Demise

**DOI:** 10.3390/ijms25073925

**Published:** 2024-03-31

**Authors:** Natale Badalamenti, Antonella Maggio, Gianfranco Fontana, Maurizio Bruno, Marianna Lauricella, Antonella D’Anneo

**Affiliations:** 1 Department of Biological, Chemical and Pharmaceutical Sciences and Technologies (STEBICEF), University of Palermo, Viale delle Scienze, 90128 Palermo, Italy; antonella.maggio@unipa.it (A.M.); gianfranco.fontana@unipa.it (G.F.); maurizio.bruno@unipa.it (M.B.); antonella.danneo@unipa.it (A.D.); 2 NBFC—National Biodiversity Future Center, Piazza Marina 60, 90133 Palermo, Italy; 3 Centro Interdipartimentale di Ricerca “Riutilizzo Bio-Based Degli Scarti da Matrici Agroalimentari” (RIVIVE), University of Palermo, Viale delle Scienze, 90128 Palermo, Italy; 4 Department of Biomedicine, Neurosciences and Advanced Diagnostics (BIND), Institute of Biochemistry, University of Palermo, Via del Vespro 129, 90127 Palermo, Italy; marianna.lauricella@unipa.it

**Keywords:** atractyligenin derivatives, apoptotic cell death, chromatin fragmentation, Poly [ADP-ribose] polymerase 1, HCT116, Caco-2

## Abstract

The antitumor activity of different *ent*-kaurane diterpenes has been extensively studied. Several investigations have demonstrated the excellent antitumor activity of synthetic derivatives of the diterpene atractyligenin. In this research, a series of new synthetic amides and their 15,19-di-oxo analogues obtained from atractyligenin by modifying the C-2, C-15, and C-19 positions were designed in order to dispose of a set of derivatives with different substitutions at the amidic nitrogen. Using different concentrations of the obtained compounds (10–300 μM) a reduction in cell viability of HCT116 colon cancer cells was observed at 48 h of treatment. All the di-oxidized compounds were more effective than their alcoholic precursors. The di-oxidized compounds had already reduced the viability of two colon cancer cells (HCT116 and Caco-2) at 24 h when used at low doses (2.5–15 μM), while they turned out to be poorly effective in differentiated Caco-2 cells, a model of polarized enterocytes. The data reported here provide evidence that di-oxidized compounds induced apoptotic cell death, as demonstrated by the appearance of condensed and fragmented DNA in treated cells, as well as the activation of caspase-3 and fragmentation of its target PARP-1.

## 1. Introduction

Cancer is a leading cause of death worldwide, with nearly 10 million deaths estimated in 2020 [1]. Globally, nearly one in five deaths is due to cancer. At the beginning of 2023, the World Health Organization highlighted that approximately 70% of cancer deaths occur in low- and middle-income countries [2]. Among all types, colorectal cancer is the fourth most common cancer in the world [3]. The onset and progression of colorectal cancer is caused by a combination of multiple factors, with age, gender (it has a higher incidence in women than in men), family history, region, and personal history being the major risk factors [4].

Conventional treatments for colorectal cancer involve the use of surgery and/or chemotherapy. Drugs used for treatment can lead to cancer cell death by causing DNA damage or triggering sudden signaling pathways, including cell cycle arrest, global translation inhibition, DNA repair, etc. [5]. However, it has been shown in many studies, including molecular pathological epidemiology studies, that the use of chemotherapy in patients with colorectal cancer varies according to the severity of the disease. The effects of cytotoxicity, drug resistance, and adverse reactions are the main problems associated with chemotherapy [6].

Natural products isolated from plants have led to the design and synthesis of a great number of biologically active compounds. They have shown excellent bioactivities, such as anti-inflammatory, antiviral [7], and antibacterial [8] properties.

In the last decade, metabolites of the genus *Isodon* (Benth.) Schrad. ex Spach, plants of the Lamiaceae family, have been biologically investigated, and they have proven to be promising phytopharmaceuticals due to their wide range of physiological effects, such as the inhibition of hepatitis viral replication [9] and bacterial infections of the lung or intestine [10], as well as antimalarial [11], anti-inflammatory [12], and anticancer properties [13]. One of the most renowned *ent*-kauranic compounds is kaurenoic acid. Various biological assays have been carried out on this metabolite, attributing important properties to it. It is able to attenuate inflammatory mechanisms through various processes, such as the activation of the nuclear factor E2-related factor-2 (Nrf2) [14,15,16], inducing regulations in T-helper 2 (Th2) and nuclear factor-κB (NF-κB) pathways linked to specific cytokines [17,18]. This particular acid is also able to dose-dependently inhibit the release of prostaglandin E2, influencing the production of nitric oxide (NO) through inducible nitric oxide synthase (iNOS) and cyclooxygenase-2 [19,20,21,22]. The ability to inhibit appropriate cytokines makes it an excellent analgesic product. This is because it is responsible for the activation of the NO cyclic GMP kinase G-ATP protein-sensitive potassium channel signaling pathway [23].

At the anticancer level, dynamics such as genotoxicity and mutagenicity must be seriously taken into consideration. Indeed, kaurenoic acid is considered a mutagenic compound according specific studies carried out in vitro and in vivo [24,25]. In fact, clearly genotoxic and mutagenic effects have been found in human blood leukocytes and on various mouse and hamster cellular structures [24,25]. It is assumed, from these studies, that the effect of this acid is mainly linked to the ability to break the filamentary structures of DNA [25]. Structure–activity relationship studies have demonstrated that the double bond on C-16 is responsible for its genotoxicity [25]. Considering these aspects, it was also highlighted that this compound shows important cytotoxic effects on several cell lines, such as HeLa, A-549, HEp-2, PC-3, and MCF-7, acting in a dose-dependent manner [26], and many of its derivatives have shown antitumor activity against several cancer cell lines, like MCF-7 [27], 4T1 [28], and U87 [29], as well as other tumor lines [30].

**Figure 1:**
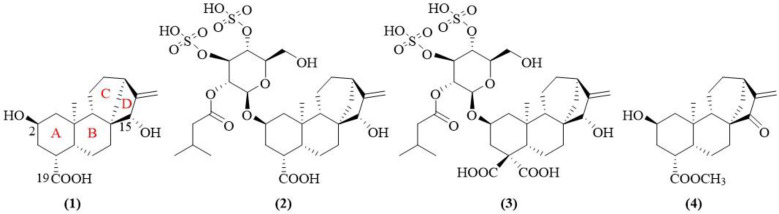
Structures of atractyligenin (**1**), atractyloside (**2**), carboxyatractyloside (**3**), and 15-ketoatractyligenin methyl ester (**4**).

So far, more than 700 *ent*-kauranic-type diterpenoids with compact polycyclic ring systems have been isolated from the genus *Isodon*, especially the oridonin derivatives [31]. A common structural unit present in these diterpene compounds is the *α,β*-unsaturated ketone in the D ring. Unfortunately, however, the use of these natural compounds as anticancer agents has been hampered by their moderate potency [31]. Therefore, it is necessary to find new compounds with simple structures and more robust activity. Among the diterpenes with an *ent*-kaurenic structure, atractyligenin (**1**) (Figure 1), the aglycone of the highly toxic molecules atractyloside (**2**) and carboxyatractyloside (**3**) extractable in good amounts from *Chamaeleon gummifer* (L.) Cass., has undergone numerous chemical modifications in order to explore the biological properties of its derivatives.

These investigations concerned the photoinduced functionalization of its C-20 methyl group, enzyme-catalysed transformations of its alcohol groups [32], and the synthesis of several oxidative derivatives with promising cytotoxic activity against some human cancer cell lines, including A549 (lung), PC-3 (prostate), 1A9 (ovary), MCF-7 (breast), KB (nasopharynx), and KB-VIN (stresses multidrug-resistant KB) [33,34]. One of them, 15-ketoatractyligenin methyl ester (**4**) (Figure 1), has shown potent tumour cell growth inhibition activity, the mechanism of action of which has been widely elucidated [34,35]. According to the literature, the presence of the *α*,*β*-unsaturated carbonyl moiety seems to be important for the antiproliferative activity [33,36]. Previous studies on the thioredoxin system (TrxR) have also demonstrated that the catalytic thiols of TrxR and/or the exposed selenocysteine [37,38] are good Michael donors for the *α*,*β*-unsaturated carbonyl group present in **4**. The reactivity of the electrophilic group towards the Cys/Sec system is also supported by recent results on the Trx-inhibitory activity of oridonine [39]. Furthermore, the evaluation of the antibiotic properties of **1**, **2**, and **4** against *Enterococcus faecalis*, *Escherichia coli*, and *Staphylococcus aureus* [40]; their anti-leishmania activity [41]; and the inhibition of skin photoaging by atractyligenin (**1**) [42] have recently been reported.

In 2022 it was demonstrated that synthetic modifications (bromination, reduction, elimination, and oxidation) on the A ring of **1** modulated its antitumor activity, significantly increasing the antiproliferative efficacy in some cases [43].

Herein, we report the synthesis of modified derivatives on C-2 and C-19 of the A ring and on C-15 of the D ring of **1** using a semi-synthetic approach that involves the amidation of the carboxyl moiety and the oxidation of the alcoholic functionalities. Finally, the antiproliferative activity of all the designed compounds was evaluated at different concentrations and at different times on two lines of colon cancer cells, namely HCT116 and Caco-2, in addition to investigation of the effects on DNA and the mechanism of action.

## 2. Results and Discussion

### 2.1. Synthesis and Spectroscopical Characterization

**Scheme 1:**
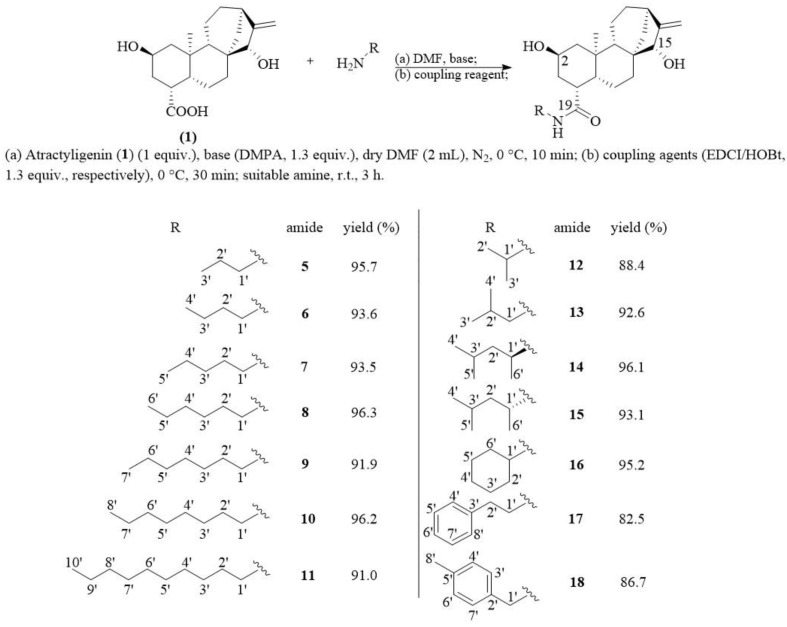
General procedure for the synthesis of atractyligenin amides **5**–**18**.

The design and synthesis of C-19-modified analogues of **1** were successfully processed by an amidation reaction, as reported in Scheme 1. The treatment of atractyligenin (**1**) with a base (DMAP) and coupling reagents EDCI/HOBt in the presence of the suitable amine led to attainment of a small library of amide derivatives (**5**–**18**) (Scheme 1). Altogether, fourteen compounds were prepared, including seven bearing a linear alkyl chain (**5**–**11**), four with a branched alkyl pendant (**12**–**15**), one bearing an aliphatic ring such as cyclohexylamine (**16**), and two bearing an alkyl aryl pendant (**17**–**18**).

After the standard purification procedures, all the synthesized compounds were characterized with extensive spectroscopic and spectrometric analyses, including HRESIMS and ^1^H-NMR, as well as ^13^C-NMR spectral analysis, with the support of homotopic and heterotopic correlations such as COSY, HSQC, HMBC, and NOESY techniques. All NMR and mass spectra of compounds **5**–**18** are included as Supplementary Material file (Figures S1–S47). The amides of **5**–**18** have similar spectroscopic data. By way of example, the diagnostic analysis of compound **5** is discussed below. In the ^13^C-NMR spectrum of **5** (Figure S2), the diagnostic signal at 174.29 ppm can be attributed to a quaternary sp^2^ amide carbon (C-19), while the signal assignable to the carbon sp^3^ of the methylene group (in *α*, to the nitrogenous function) was observed at 41.30 ppm (C-1′). To confirm the successful amidation, the signal at *δ* 3.13 (m, C*H*_2_-1′) in the ^1^H-NMR spectrum (Figure S1), characteristic of the CH_2_-1′ group, showed an HSQC correlation with the C1′ carbon at 41.30 ppm (Figure S4) and an HMBC correlation (Figure S6) with the C-19 carbonilic carbon (174.29 ppm). These signals, together with those expected for the diterpenoid moiety and for the aliphatic portion of the amide pendant, confirmed the formation of atractyligenin *N*-propylamide **5**. The assignments were also corroborated by DEPT (Figure S3), COSY (Figure S5), HSQC, and HMBC, in particular from the correlations between H_2_-3, H-4, H-5, H_2_-1′, and C-19—especially NOESY correlations (Figure S7) which were absent between the H-2, the methylene protons C*H*_3_-20, and the proton in position 4 in this case—reconfirming an *α* orientation for the amide function at position 19. The other amides (**6**–**18**) showed NMR data, including 2D-NMR correlations, very similar to those observed for **5**, thus allowing their structures to be unambiguously determined and all the ^1^H and ^13^C-NMR signals to be fully assigned, as reported in the Section 3.

**Scheme 2:**
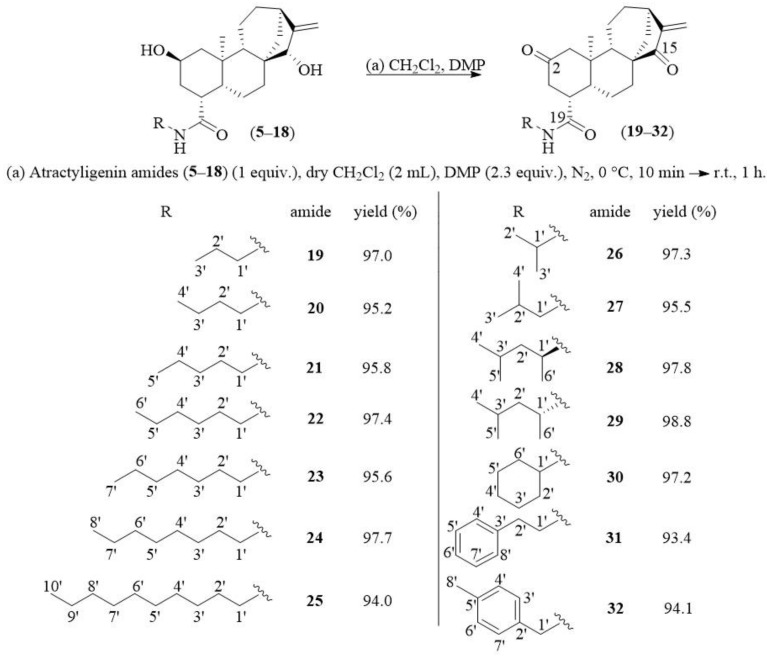
Synthesis of di-oxidates of atractyligenin amides (**19**–**32**).

In order to evaluate the importance of the oxidation states of C-2 and C-15 in terms of biological activity, it was planned to transform the two hydroxyl groups into the corresponding ketones (Scheme 2). All NMR and mass spectra of di-oxo-atractyligenin amides (**19**–**32**) are included in the Supplementary Materials(Figures S48–S89). Also for the case of oxidations, only the model characterization of the modified amide **5** is reported. All the previously obtained amides (**5**–**18**) were reacted with DMP, a hypervalent iodine compound that is suitable for a mild oxidation of secondary alcohols to ketones. After one hour, the starting reagent disappeared on TLC, and a single product (**19**) was obtained from amide **5**. Its proton and carbon spectra (Figures S48 and S49, respectively) showed a clear shift at the low field in the ^1^H-NMR of exocyclic protons (*δ*_H_ 5.96, H-17a; *δ*_H_ 5.28, H-17b), the presence of two ketones function at *δ*_C_ 2 × 209.78 (C-2 and C-15), and the absence of alcohol signals in the proton spectrum. Consequently, compound **19** is consistent with the structure of the di-oxo derivative of atractyligenin amide **5**. The other di-oxo amides (**20**–**32**) obtained by the same oxidative treatment showed NMR data very similar to those observed for **19**, allowing us to determine their structures and, therefore, to completely assign all the proton and carbon signals, as reported in the Section 3.

### 2.2. Investigation of the Cytotoxic Activity of Compounds ***1*** and ***5***–***32*** on Colon Cancer Cells

**Figure 2:**
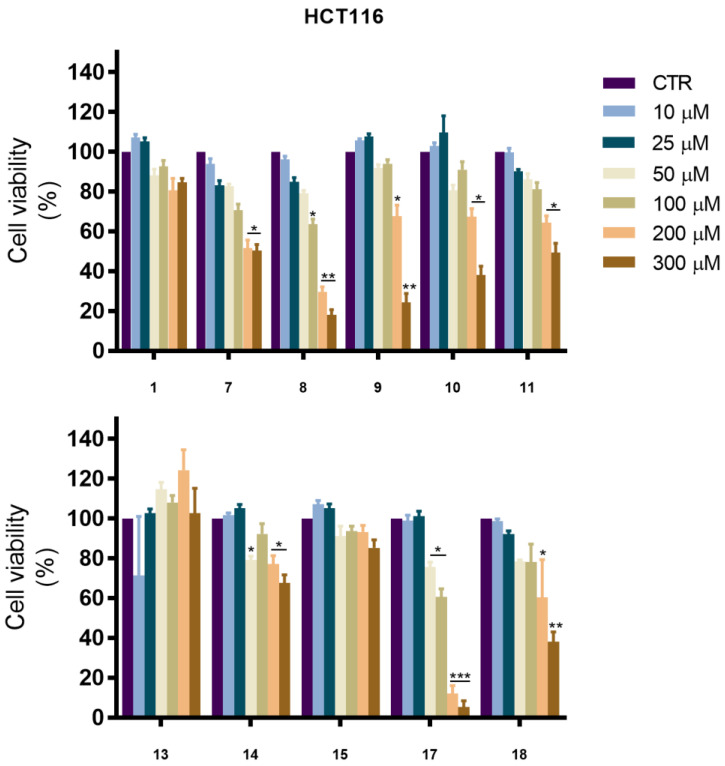
Effects of atractyligenin and its amide derivatives on cell viability of HCT116 colon cancer cells. Cells were incubated in the presence of compounds for 48 h; then, cell viability was assessed by MTT assay as reported in Section 3. Each value reported in the histogram represents the mean of three independent experiments ± SD. Significant differences compared with the untreated sample are represented by (*) *p* < 0.05, (**) *p* < 0.01, and (***) *p* < 0.001 calculated by Student’s *t*-test and one-way analysis of variance.

Both the di-hydroxy amides (**5**–**18**) and the related di-oxo amides (**19**–**32**) obtained from the diterpene atractyligenin were tested on HCT116 colon cancer cells by MTT assays using a wide range concentration of all compounds (10–300 µM). Interestingly, although **1** used at high concentrations did not cause significant changes in colon cancer cell viability after 48 h of treatment, amide derivatives showed remarkable cytotoxic effects only at the highest doses used (200 and 300 μM) (Figure 2). The very low cytotoxic activity of **1** is known. In fact, it was demonstrated that atractyligenin was not biologically active against several human tumor cell lines, including A549 (lung), PC-3 (prostate), 1A9 (ovarian), MCF-7 (breast), KB (nasopharyngeal), and KB-VIN (multidrug-resistant KB subline) [33].

Derivatives **19**–**32** were also tested for 48 h for their effects on colon cancer HCT116 cells. It is interesting to note that the introduction of two ketone functionalities at the C-2 and C-15 positions caused an increase in antiproliferative effectiveness by 8–10 times compared to the corresponding derived amides (**5**–**18**).

**Figure 3:**
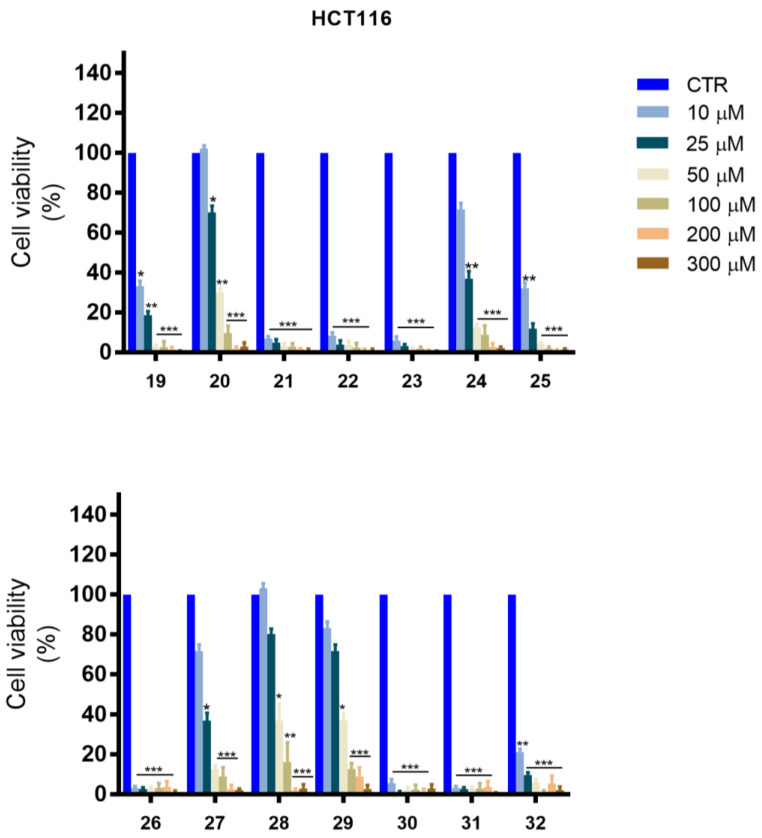
Effects of high doses of **19**–**32** on HCT116 cell viability after 48 h of treatment. Values reported in the histogram represent the mean of three independent experiments ± SD. Significant differences compared with the untreated sample are represented by (*) *p* < 0.05, (**) *p* < 0.01, and (***) *p* < 0.001 calculated by Student’s *t*-test and one-way analysis of variance.

As shown in Figure 3, except for compounds **20**, **27**, and **32**, almost all other di-oxidates were capable of reducing the cell viability at a 10 μM dose (Figure 3). These data show that di-ketones are endowed with a remarkable efficacy in comparison to diols **5**–**18**, indicating that the functional-group interconversion from alcohol to ketone on both C-2 and C-15 is fundamental for increasing the antiproliferative power of atractyligenin derivatives.

**Figure 4:**
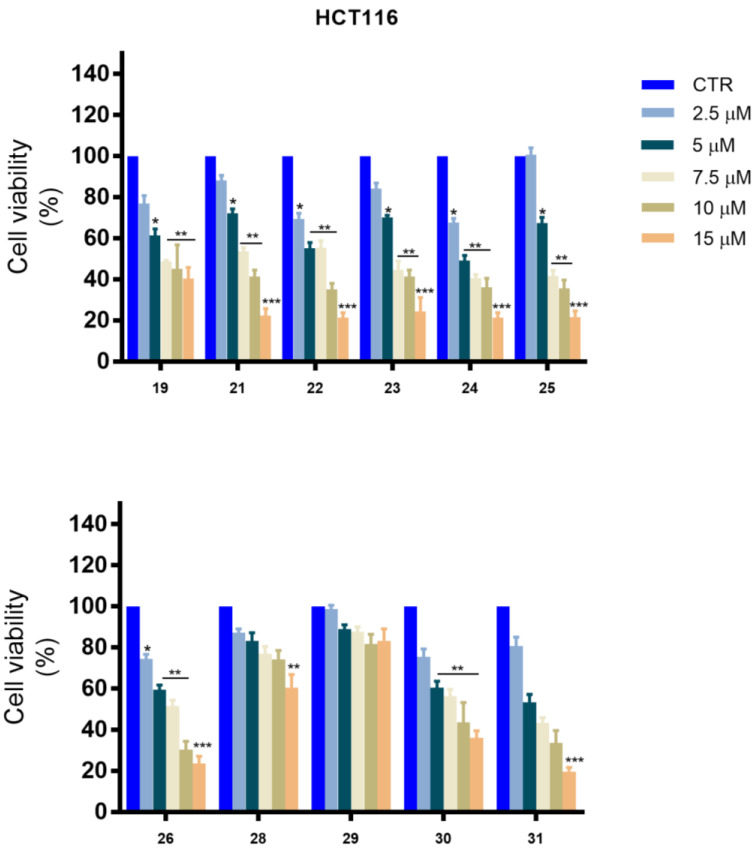
Effects of low doses of some di-oxidated derivates of atractyligenin amides on HCT116 cell viability at 24 h. Values reported in the histogram represent the mean of three independent experiments ± SD. Significant differences compared with the untreated sample are represented by (*) *p* < 0.05, and (**) *p* < 0.01, (***) *p* < 0.001 calculated by Student’s *t*-test and one-way analysis of variance.

In light of the obtained results, the subsequent experiments were performed by incubating colon cancer cells with lower concentrations of the most active di-oxidates (range: 2.5–15 μM). Interestingly, as can be observed in the bar charts reported in Figure 4, except for **28** and **29**, which showed scarce effects at low doses, all other tested compounds displayed clear cytotoxic effects in a dose-dependent fashion on HCT116 cells. Such an effect was already clearly visible at 24 h of incubation (Figure 4).

The insertion of additional nonpolar pharmacophores on the A ring of atractyligenin, as well as the introduction of the two ketone functionalities, led to significant results, contributing to the increase its antiproliferative efficacy in different ways.

Indeed, the targeting capacity of *α*,*β*-unsaturated carbonyl systems, such as those present in derivatives **19**–**32**, has been demonstrated [44]. This functionality can inhibit the activity of the thioredoxin (TrxR) system, comprising the coenzymes nicotinamide adenine dinucleotide phosphate (NADPH), thioredoxin (Trx), and Trx reductase (TrxR), in both cell-free systems and in Jurkat cells. Trx controls nuclear translocation and/or the activity of several transcription factors and those that prevent apoptosis. Trx, among other things, protects mitochondria by the opening of the permeability transition pore (PTP) [44] and the release of cytochrome *c*, in addition to inhibiting the activation of apoptosis signaling kinase 1 (ASK1) [45]. The presence of functional thiol systems in Trx and TrxR [46] therefore makes these proteins suitable targets for the *α*,*β*-unsaturated carbonyl functional group [37,38]. This aspect was also confirmed on synthetic derivatives of oridonin, a natural compound with a basic structure very similar to that of atractyligenin. All oridonin A-ring-modified compounds containing the *α*,*β*-unsaturated ketone presented IC_50_ values ranging between 1.22 and 11.13 μM on four different human cell lines after 72 h of incubation [31].

Table 1 presents reported IC_50_ values of some di-oxidated derivates tested on HCT116 cancer cells after 24 h of treatment. Among the compounds tested, except for compounds **28** and **29**, all derivative analogues were significantly more potent than **1** and the corresponding amides (**5**–**18**).

Compound **24** was the most active of the series, with an IC_50_ value of 5.35 μM—very similar to the activity shown by compound **25** (IC_50_ = 5.50 μM). Both compounds have a very similar structure, the first bearing an octyl linear chain on the amidic nitrogen, while the second is endowed with a decyl chain. The activity tended to worsen slightly when using short (compound **19**) or medium (compounds **21**–**23**) chains as *N* substituents or chains with small branches (compound **26**). Finally, the introduction of an aromatic pendent (compound **31**), compared to a totally aliphatic cycle (presented in **30**), led to a clear improvement in the antiproliferative activity (IC_50_ = 6.37 μM for **31** and 9.27 μM for **30**).

**Figure 5:**
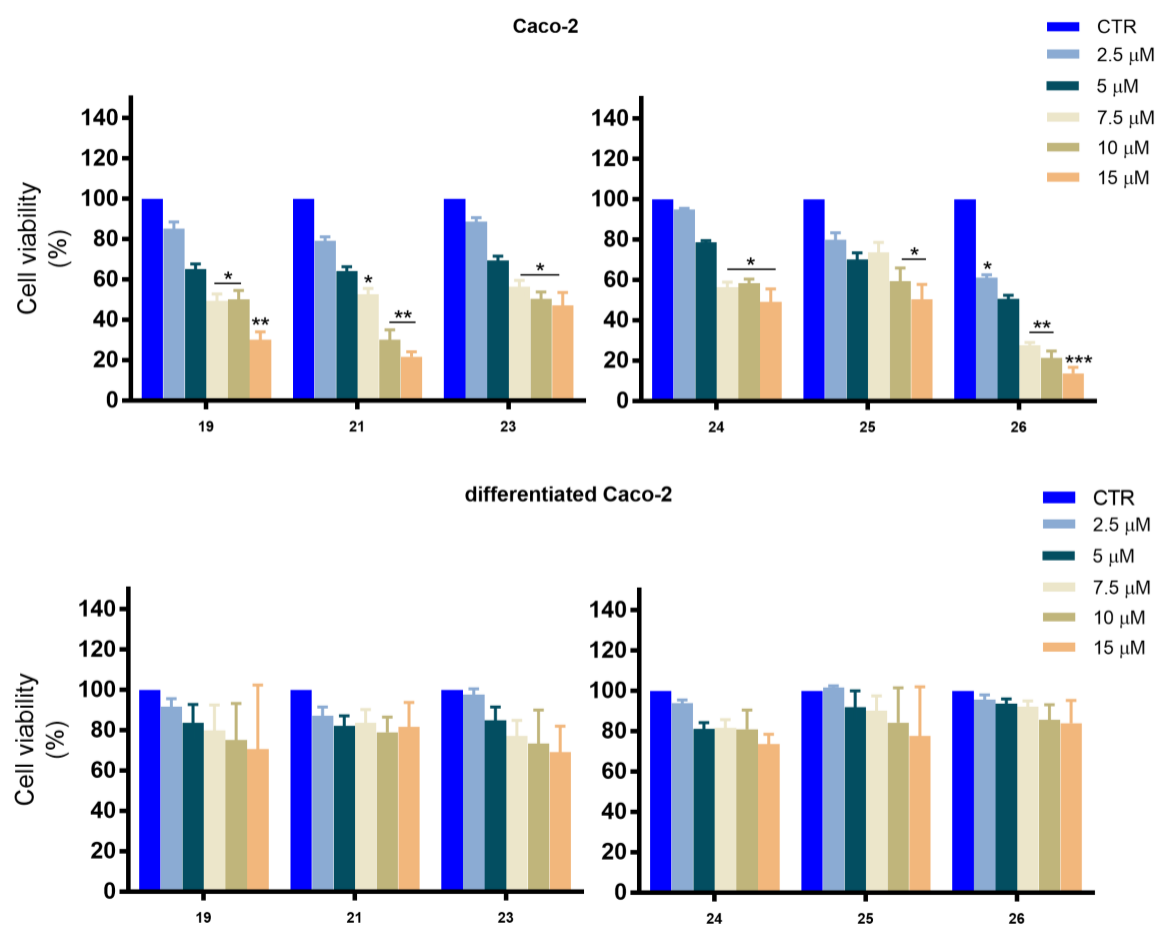
Effects of low doses of some di-oxidated derivates of atractyligenin amides on colon cancer Caco-2 cells and differentiated Caco-2 cells at 24 h of treatment. Values reported in the histogram represent the mean of three independent experiments ± SD. Significant differences compared with the untreated sample are represented by (*) *p* < 0.05, (**) *p* < 0.01, and (***) *p* < 0.001 calculated by Student’s *t*-test and one-way analysis of variance.

The effect of the most active compounds was also evaluated on Caco-2 cells, another colon cancer cell line, as well as on differentiated Caco-2 cells, a model of polarized colon cells grown in culture according to the procedure reported by Natoli et al. [46] (Figure 5). It is noteworthy that cytotoxic effects also appeared in Caco-2 tumor cells, while no cytotoxicity was observed in differentiated Caco-2. The absence of any cytotoxic effect in this enterocyte-like model [47,48] suggests that the tested compounds exert a selective efficacy on tumor cells. However, we aim to better explore this aspect in our future research.

Indeed, a comparative analysis of the IC_50_ values in both colon cancer cells, HCT116 and Caco-2, after 24 h of treatment (Table 2) unveiled **23** and **24** among the most efficacious compounds in reducing viability in Caco-2 cells, while compounds **24** and **25** were the most active for the HCT116 cell line. The activity of compounds **19** and **25** worsened considerably compared to that shown on the HCT116 cell line, while the activity of compounds **21** and **26** improved slightly on the Caco-2 cell line.

**Figure 6:**
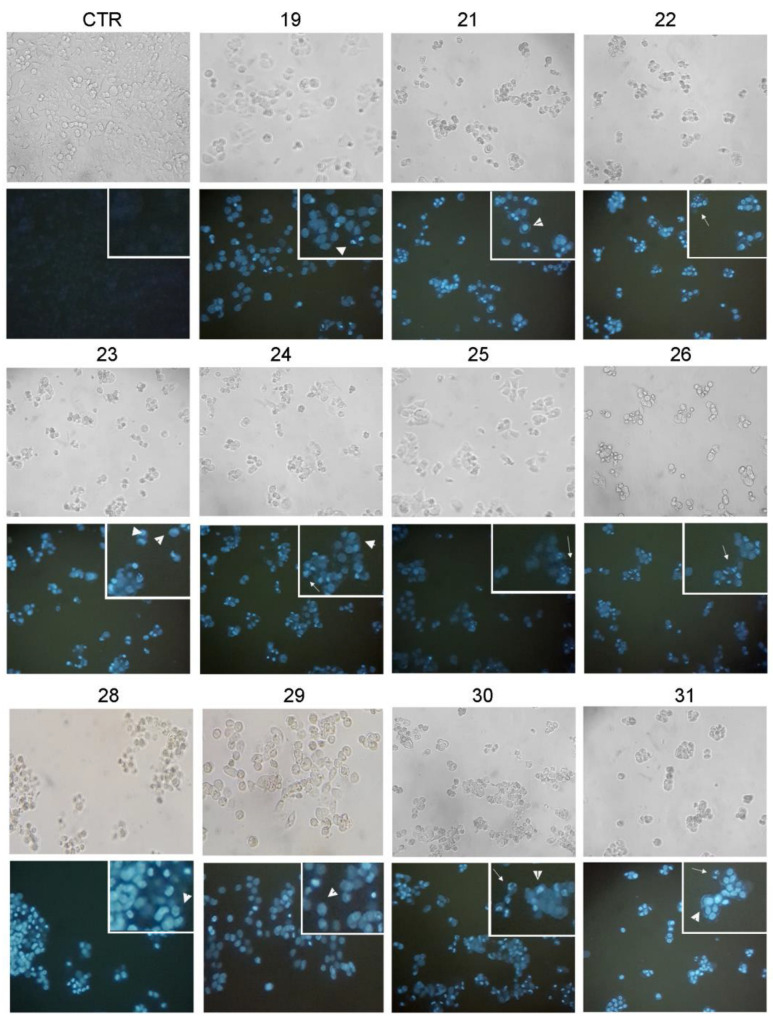
Effects of some di-oxidated derivates of atractyligenin amides on chromatin condensation and fragmentation visualized by Hoechst staining. Cells were treated with a 10 μM dose for 24 h; then, morphological analysis of HCT116 cells was performed under light microscopy (**upper panel**). After Hoechst 33342 staining, condensed or fragmented chromatin (**lower panel**) was visualized using a DAPI filter in a Leica inverted fluorescence microscope. The condensation of the nuclear material (arrowheads), followed by the formation of apoptotic bodies (arrows), is indicated in the figure. White squares correspond to a magnification of stained nuclei. Pictures were taken by Leica Q Fluoro Software (version 2.0, Leica Microsystems S.r.l, Wetzlar, Germany, original magnification of 200×).

To ascertain whether the effects exerted by active derivatives of atractyligenin on colon HCT116 cancer cells could be ascribed to the induction of apoptosis, it was evaluated whether chromatin condensation and its fragmentation (two typical changes of apoptotic cell death) occurred in treated cells. As shown in Figure 6, light microscopy analyses revealed that all tested compounds caused clear morphological changes at 24 h represented by cell detachment and shrinkage, as well as a consistent reduction in cellular density in comparison to untreated samples (upper panel in Figure 6). Interestingly, vital Hoechst staining (lower panel) highlighted that compounds **19**, **21**–**26**, and **28**–**31** caused the condensation of the nuclear material, as well as the formation of apoptotic bodies.

**Figure 7:**
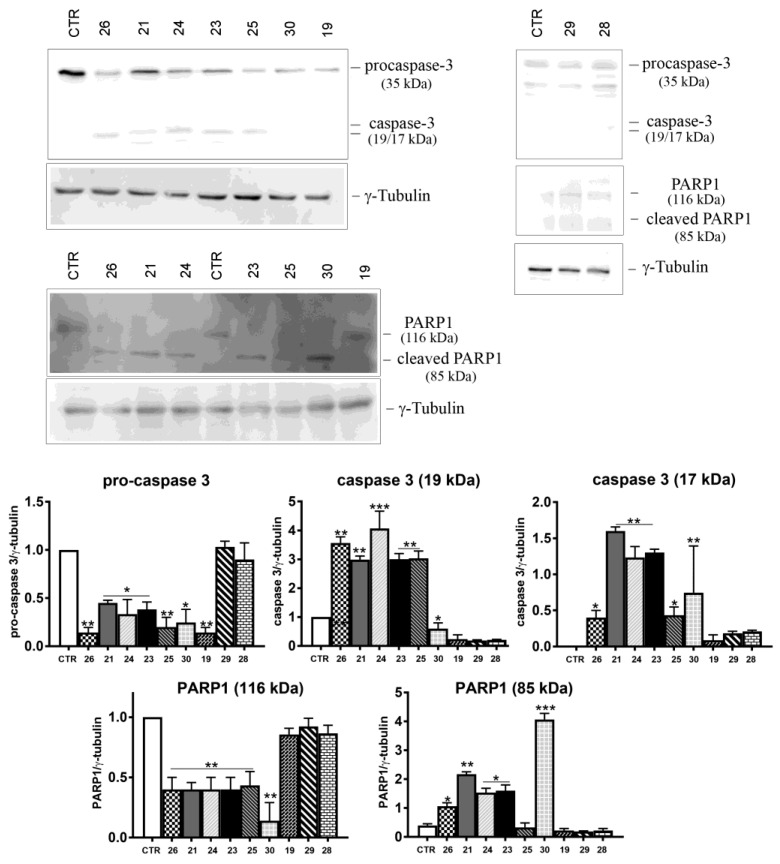
Western blotting analysis of the apoptotic markers caspase-3 and PARP-1 in HCT116 cells incubated in the presence of a 10 μM dose of di-oxidated derivates of atractyligenin amides for 24 h. Each band was determined by densitometric analysis and normalized with *γ*-tubulin used as a loading control. Bar charts of densitometric analyses report the mean values of three independent experiments. Significant differences compared with the untreated sample are represented by (*) *p* < 0.05, (**) *p* < 0.01, and (***) *p* < 0.001 calculated by Student’s *t*-test and one-way analysis of variance.

In addition, Western blotting analyses were performed to elucidate the possible involvement of caspase-3 and PARP-1, typical apoptotic players [49,50]. In particular, it was determined that all active compounds caused a downregulation of pro-enzymatic caspase-3 and the appearance of the fragmented and active forms with molecular weights of 19 and 17 kDa, respectively. Compounds **28** and **29**, which were not found to be significantly cytotoxic, did not show an appreciable effect on pro-caspase 3, confirming the relevant role of this protein in determining apoptosis in this cell line. On the other hand, the cytotoxicity of **19** does not seem to be related to an effect on the pro-caspase 3 pathway. A similar trend can be observed with the fragmentation of PARP-1, a typical caspase-3 target (Figure 7) commonly cleaved in apoptotic cell death programs. All active compounds, except **19**, possess the ability to promote the fragmentation of both pro-caspase 3 and PARP-1. Also in this case, non-cytotoxic **28** and **29** do not exert any influence on PARP-1. The anomaly observed in the behaviour of compound **19** may suggest the involvement of other pathways in the cytotoxicity of this compound. Further investigations are on the way. Upon observing the chemical structures of the molecules discussed above, it comes into evidence that the more active compounds **24** and **25** possess a long, linear aliphatic chain (8C and 10C, respectively) linked to amide-nitrogen. On the contrary, molecules containing highly branched side chains, that is, **28** and **29**, are not active. The bioactivity of the remaining set of tested molecules lies in between those extremes. This mere hypothesis, although quite reasonable, must be confirmed by docking investigations.

## 3. Materials and Methods

### 3.1. Experimental Section

Optical rotations were measured in a CH_3_OH solution on a JASCO P-1010 digital polarimeter (Lecco, Italy) at 25 °C and at 589 nm. The NMR spectra were run on a Bruker Avance II (Milan, Italy) instrument operating at 600 MHz for ^1^H-NMR and at 125 MHz for ^13^C-NMR. Chemical shifts (*δ*) were indirectly referred to tetramethylsilane using residual solvent signals. Deuterated solvents such as CDCl_3_-*d*_1_, DMSO-*d*_6_, and CD_3_OD-*d*_4_ were used for the solubilization of the various synthesized compounds. Residual solvent signals of *δ* = 7.27 ppm in ^1^H and *δ* = 77.00 ppm in ^13^C for CDCl_3_-*d*_1_*, δ* = 2.50 ppm in ^1^H and *δ* = 39.51 ppm in ^13^C for DMSO-*d*_6_, and *δ* = 3.31 ppm in ^1^H and *δ* = 49.15 ppm in ^13^C for CD_3_OD-*d*_4_ were used as references in NMR spectra. DEPT, ^1^H-^1^H-COSY, HMBC, HSQC, and NOESY experiments were performed using Bruker microprograms. Mass spectra were obtained using an HPLC-ESI-QTOF HRMS apparatus (Agilent, Milan, Italy). The HPLC system was an Agilent 1260 Infinity. A reversed-phase C_18_ column (ZORBAX Extended-C_18_ 2.1 × 50 mm, 1.8 μm) with a Phenomenex C_18_ security guard column (4 × 3 mm) was used. The flow rate was 0.4 mL/min, and the column temperature was set to 30 °C. The mass spectra was recorded using an Agilent 6540 UHD accurate-mass Q-TOF spectrometer equipped with a Dual AJS ESI source working both in negative and positive modes. Merck Si gel (70–230 mesh) deactivated with 15% H_2_O was used for column chromatography. TLCs were performed on silica gel (Merck, Kieselgel 60 F_254_, 0.25 and 0.50 mm) plates. The spots were visualized by spraying with 5% 4-anisaldehyde in EtOH acid. All chemicals, such as dried DMF, DMAP, EDCI, HOBt, and Dess–Martin reagent, were purchased from Sigma Aldrich (St. Louis, MO, USA) and used without further purification; H_2_O, CH_3_CN, and HCOOH for HPLC-UV were of special grade (Carlo Erba, Milan, Italy).

### 3.2. Isolation of the Atractyligenin (***1***)

Atractyligenin (**1**) was extracted from the dried roots of *C. gummifer* Cass. as previously reported [43].

### 3.3. General Procedure for the Synthesis of Atractyligenin Amides (***5***–***18***)

**Figure 8:**
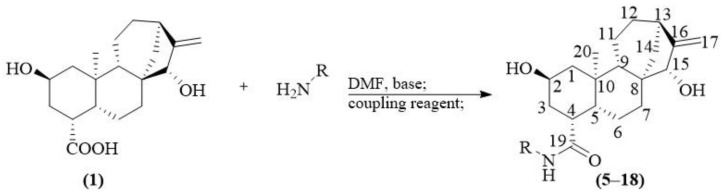
General procedure for the synthesis of amides **5**–**18**.

A general procedure previously reported for the synthesis of rosmarinic acid amides by Cardullo et al. [51] was followed for the synthesis of amides **5**–**18** of 1 (Figure 8). Atractyligenin (1.0 equiv.) was dissolved in dry DMF (2 mL) in a three-necked flask previously filled with N_2_, and DMAP (1.3 equiv.) was added. The mixture was stirred at 0 °C for 10 min; then, EDCI (1.3 equiv.) and HOBt (1.3 equiv.) were added dropwise to the solution with a syringe. The mixture was stirred at 0 °C for 30 min under an N_2_ atmosphere. Finally, the suitable amine (1.3 equiv.) was added to the mixture, stirring for 3 h at r.t. The crude reaction was evaporated under vacuum to remove the solvent, and the residue was partitioned between EtOAc (20 mL) and 1 N HCl (3 × 20 mL); then, the organic layer was partitioned with saturated NaHCO_3_ solution (3 × 20 mL). The organic phase was washed with water, dried over anhydrous Na_2_SO_4_, filtered, and taken to dryness. Each residue was purified by classic liquid column chromatography.

- Compound **5**.

The crude mixture was purified on a silica gel column eluting with CH_2_Cl_2_-MeOH (98:2) to afford a fraction containing compound **5** (21.5 mg, 95.72%) as an amorphous yellow solid; [*α*${]}_{D}^{25}$–135.04 (*c* 0.10, CHCl_3_); ^1^H-NMR (CDCl_3_-*d*_1_, 250 MHz) *δ* 0.68 (1H, dd, 11.7, 11.7 Hz, H-1*β*), 2.17 (1H, ddd, 11.7, 3.7, 1.7 Hz, H-1*α*), 4.40 (1H, dddd, 11.7, 11.7, 3.7, 3.7 Hz, H-2*α*), 1.82 (1H, ov, H-3*α*), 1.35 (1H, ov, H-3*β*), 2.48 (1H, brt, H-4*β*), 1.41 (1H, ov, H-5*β*), 1.76 (1H, ov, H-6*α*), 1.57 (1H, ov, H-6*β*), 1.49 (1H, ov, H-7*β*), 1.74 (1H, ov, H-7*α*), 1.02 (1H, brd, H-9*β*), 1.39 (1H, ov, H-11*β*), 1.55 (1H, ov, H-11*α*), 1.49 (1H, ov, H-12*β*), 1.36 (1H, ov, H-12*α*), 2.70 (1H, brt, H-13), 2.28 (1H, ddd, 12.7, 3.7, 1.7 Hz, H-14*α*), 1.40 (1H, ov, H-14*β*), 3.78 (1H, brs, H-15*β*), 5.18 (1H, brs, H-17a), 5.04 (1H, brs, H-17b), 0.96 (3H, s, CH_3_-20), 3.13 (2H, m, CH_2_-1′), 1.22–1.48 (2H, ov, CH_2_-2′), 0.89 (3H, t, 7.3 Hz, CH_3_-3′) (Figure S1); ^13^C-NMR (CDCl_3_-*d*_1_, 62.5 MHz) *δ* 48.96 (C-1), 64.05 (C-2), 37.65 (C-3), 45.20 (C-4), 48.71 (C-5), 25.86 (C-6), 34.73 (C-7), 47.34 (C-8), 52.67 (C-9), 40.26 (C-10), 17.98 (C-11), 32.17 (C-12), 42.05 (C-13), 36.00 (C-14), 82.36 (C-15), 159.57 (C-16), 108.43 (C-17), 174.29 (C-19), 16.39 (C-20), 41.30 (C-1′), 22.70 (C-2′), 11.46 (C-3′) (Figure S2); HRESIMS *m*/*z* 362.2703 [M + H]^+^ (calcd. for C_22_H_35_NO_3_, 362.2690) (Figure S8).

- Compound **6**.

The crude mixture was purified on a silica gel column eluting with CH_2_Cl_2_-MeOH (98:2) to afford a fraction containing compound **6** (23.1 mg, 93.64%) as an amorphous yellow solid; [*α*${]}_{D}^{25}$–155.91 (*c* 0.10, CHCl_3_); ^1^H-NMR (CDCl_3_-*d*_1_, 250 MHz) *δ* 0.71 (1H, dd, 11.7, 11.7 Hz, H-1*β*), 2.22 (1H, ddd, 11.7, 3.7, 1.7 Hz, H-1*α*), 4.47 (1H, dddd, 11.7, 11.7, 3.7, 3.7 Hz, H-2*α*), 1.80 (1H, ov, H-3*α*), 1.36 (1H, ov, H-3*β*), 2.50 (1H, brt, H-4*β*), 1.50 (1H, ov, H-5*β*), 1.78 (1H, ov, H-6*α*), 1.55 (1H, ov, H-6*β*), 1.46 (1H, ov, H-7*β*), 1.75 (1H, ov, H-7*α*), 1.05 (1H, brd, H-9*β*), 1.36 (1H, ov, H-11*β*), 1.54 (1H, ov, H-11*α*), 1.50 (1H, ov, H-12*β*), 1.49 (1H, ov, H-12*α*), 2.75 (1H, brt, H-13), 2.35 (1H, ddd, 12.7, 3.7, 1.7 Hz, H-14*α*), 1.42 (1H, ov, H-14*β*), 3.82 (1H, brs, H-15*β*), 5.22 (1H, brs, H-17a), 5.08 (1H, brs, H-17b), 0.99 (3H, s, CH_3_-20), 3.22 (2H, m, CH_2_-1′), 1.24-1.58 (2H, ov, CH_2_-2′), 1.34 (2H, ov, CH_2_-3′), 0.93 (3H, t, 7.3 Hz, CH_3_-4′) (Figure S9); ^13^C-NMR (CDCl_3_-*d*_1_, 62.5 MHz) *δ* 49.13 (C-1), 64.40 (C-2), 37.91 (C-3), 45.36 (C-4), 48.72 (C-5), 26.07 (C-6), 34.76 (C-7), 47.45 (C-8), 52.66 (C-9), 40.37 (C-10), 18.03 (C-11), 32.24 (C-12), 42.13 (C-13), 36.07 (C-14), 82.54 (C-15), 159.93 (C-16), 108.52 (C-17), 174.30 (C-19), 16.43 (C-20), 39.34 (C-1′), 31.63 (C-2′), 20.21 (C-3′), 13.72 (C-4′) (Figure S10); HRESIMS *m*/*z* 376.2866 [M + H]^+^ (calcd. for C_23_H_37_NO_3_, 376.2846) (Figure S11).

- Compound **7**.

The crude mixture was purified on a silica gel column eluting with CH_2_Cl_2_-MeOH (98:2) to afford a fraction containing compound **7** (23.9 mg, 93.50%) as an amorphous yellow solid; [*α*${]}_{D}^{25}$–293.21 (*c* 0.10, CHCl_3_); ^1^H-NMR (CDCl_3_-*d*_1_, 250 MHz) *δ* 0.66 (1H, dd, 11.7, 11.7 Hz, H-1*β*), 2.15 (1H, ddd, 11.7, 3.7, 1.7 Hz, H-1*α*), 4.38 (1H, dddd, 11.7, 11.7, 3.7, 3.7 Hz, H-2*α*), 1.81 (1H, ov, H-3*α*), 1.35 (1H, ov, H-3*β*), 2.46 (1H, brt, H-4*β*), 1.52 (1H, ov, H-5*β*), 1.80 (1H, ov, H-6*α*), 1.57 (1H, ov, H-6*β*), 1.48 (1H, ov, H-7*β*), 1.73 (1H, ov, H-7*α*), 0.98 (1H, brd, H-9*β*), 1.37 (1H, ov, H-11*β*), 1.54 (1H, ov, H-11*α*), 1.50 (1H, ov, H-12*β*), 1.48 (1H, ov, H-12*α*), 2.68 (1H, brt, H-13), 2.27 (1H, ddd, 12.7, 3.7, 1.7 Hz, H-14*α*), 1.38 (1H, ov, H-14*β*), 3.77 (1H, brs, H-15*β*), 5.16 (1H, brs, H-17a), 5.02 (1H, brs, H-17b), 0.94 (3H, s, CH_3_-20), 3.16 (2H, ov, CH_2_-1′), 1.21-1.41 (2H, ov, CH_2_-2′), 1.25 (4H, ov, CH_2_-3′,-4′), 0.84 (3H, t, 7.3 Hz, CH_3_-5′) (Figure S12); ^13^C-NMR (CDCl_3_-*d*_1_, 62.5 MHz) *δ* 48.95 (C-1), 64.03 (C-2), 37.63 (C-3), 45.18 (C-4), 48.69 (C-5), 25.85 (C-6), 34.70 (C-7), 47.32 (C-8), 52.64 (C-9), 40.23 (C-10), 17.95 (C-11), 32.15 (C-12), 42.02 (C-13), 35.95 (C-14), 82.33 (C-15), 159.53 (C-16), 108.38 (C-17), 174.16 (C-19), 16.37 (C-20), 39.48 (C-1′), 29.11 (C-2′), 29.05 (C-3′), 22.17 (C-4′), 13.87 (C-5′) (Figure S13); HRESIMS *m*/*z* 390.3033 [M + H]^+^ (calcd. for C_24_H_39_NO_3_, 390.3003) (Figure S14).

- Compound **8**.

The crude mixture was purified on a silica gel column eluting with CH_2_Cl_2_-MeOH (98:2) to afford a fraction containing compound **8** (24.1 mg, 96.29%) as an amorphous yellow solid; [*α*${]}_{D}^{25}$–120.45 (*c* 0.10, CHCl_3_); ^1^H-NMR (CDCl_3_-*d*_1_, 250 MHz) *δ* 0.67 (1H, dd, 11.7, 11.7 Hz, H-1*β*), 2.17 (1H, ddd, 11.7, 3.7, 1.7 Hz, H-1*α*), 4.40 (1H, dddd, 11.7, 11.7, 3.7, 3.7 Hz, H-2*α*), 1.84 (1H, ov, H-3*α*), 1.37 (1H, ov, H-3*β*), 2.47 (1H, brt, H-4*β*), 1.52 (1H, ov, H-5*β*), 1.81 (1H, ov, H-6*α*), 1.60 (1H, ov, H-6*β*), 1.47 (1H, ov, H-7*β*), 1.74 (1H, ov, H-7*α*), 0.99 (1H, brd, H-9*β*), 1.39 (1H, ov, H-11*β*), 1.55 (1H, ov, H-11*α*), 1.51 (1H, ov, H-12*β*), 1.45 (1H, ov, H-12*α*), 2.70 (1H, brt, H-13), 2.30 (1H, ddd, 12.7, 3.7, 1.7 Hz, H-14*α*), 1.37 (1H, ov, H-14*β*), 3.78 (1H, brs, H-15*β*), 5.18 (1H, brs, H-17a), 5.04 (1H, brs, H-17b), 0.95 (3H, s, CH_3_-20), 3.16 (2H, m, CH_2_-1′), 1.22–1.40 (2H, ov, CH_2_-2′), 1.25 (6H, ov, CH_2_-3′,-4′,-5′), 0.84 (3H, t, 7.3 Hz, CH_3_-6′) (Figure S15); ^13^C-NMR (CDCl_3_-*d*_1_, 62.5 MHz) *δ* 48.96 (C-1), 64.10 (C-2), 37.67 (C-3), 45.23 (C-4), 48.70 (C-5), 25.91 (C-6), 34.72 (C-7), 47.35 (C-8), 52.65 (C-9), 40.26 (C-10), 17.97 (C-11), 32.18 (C-12), 42.05 (C-13), 36.00 (C-14), 82.38 (C-15), 159.61 (C-16), 108.43 (C-17), 174.21 (C-19), 16.39 (C-20), 39.56 (C-1′), 29.37 (C-2′), 26.66 (C-3′), 31.34 (C-4′), 22.46 (C-5′), 13.89 (C-6′) (Figure S16); HRESIMS *m*/*z* 404.3181 [M + H]^+^ (calcd. for C_25_H_41_NO_3_, 404.3159) (Figure S17).

- Compound **9**.

The crude mixture was purified on a silica gel column eluting with CH_2_Cl_2_-MeOH (98:2) to afford a fraction containing compound **9** (23.8 mg, 91.89%) as an amorphous yellow solid; [*α*${]}_{D}^{25}$–105.84 (*c* 0.10, CHCl_3_); ^1^H-NMR (CDCl_3_-*d*_1_, 250 MHz) *δ* 0.65 (1H, dd, 11.7, 11.7 Hz, H-1*β*), 2.14 (1H, ddd, 11.7, 3.7, 1.7 Hz, H-1*α*), 4.36 (1H, dddd, 11.7, 11.7, 3.7, 3.7 Hz, H-2*α*), 1.80 (1H, ov, H-3*α*), 1.38 (1H, ov, H-3*β*), 2.45 (1H, brt, H-4*β*), 1.51 (1H, ov, H-5*β*), 1.80 (1H, ov, H-6*α*), 1.59 (1H, ov, H-6*β*), 1.49 (1H, ov, H-7*β*), 1.74 (1H, ov, H-7*α*), 0.98 (1H, brd, H-9*β*), 1.41 (1H, ov, H-11*β*), 1.52 (1H, ov, H-11*α*), 1.51 (1H, ov, H-12*β*), 1.45 (1H, ov, H-12*α*), 2.67 (1H, brt, H-13), 2.27 (1H, ddd, 12.7, 3.7, 1.7 Hz, H-14*α*), 1.37 (1H, ov, H-14*β*), 3.76 (1H, brs, H-15*β*), 5.15 (1H, brs, H-17a), 5.01 (1H, brs, H-17b), 0.93 (3H, s, CH_3_-20), 3.14 (2H, m, CH_2_-1′), 1.21–1.41 (2H, ov, CH_2_-2′), 1.23 (8H, ov, CH_2_-3′,-4′,-5′,-6′), 0.82 (3H, t, 7.3 Hz, CH_3_-7′) (Figure S18); ^13^C-NMR (CDCl_3_-*d*_1_, 62.5 MHz) *δ* 48.95 (C-1), 63.94 (C-2), 37.62 (C-3), 45.17 (C-4), 48.68 (C-5), 25.83 (C-6), 34.69 (C-7), 47.30 (C-8), 52.63 (C-9), 40.21 (C-10), 17.94 (C-11), 32.14 (C-12), 42.01 (C-13), 35.96 (C-14), 82.29 (C-15), 159.52 (C-16), 108.35 (C-17), 174.18 (C-19), 16.36 (C-20), 39.50 (C-1′), 29.36 (C-2′), 26.93 (C-3′), 28.78 (C-4′), 31.62 (C-5′), 22.38 (C-6′), 13.89 (C-7′) (Figure S19); HRESIMS *m*/*z* 418.3343 [M + H]^+^ (calcd. for C_26_H_43_NO_3_, 418.3316) (Figure S20).

- Compound **10**.

The crude mixture was purified on a silica gel column eluting with CH_2_Cl_2_-MeOH (98:2) to afford a fraction containing compound **10** (27.1 mg, 96.23%) as an amorphous yellow solid; [*α*${]}_{D}^{25}$–349.90 (*c* 0.10, CHCl_3_); ^1^H-NMR (CDCl_3_-*d*_1_, 250 MHz) *δ* 0.67 (1H, dd, 11.7, 11.7 Hz, H-1*β*), 2.17 (1H, ddd, 11.7, 3.7, 1.7 Hz, H-1*α*), 4.38 (1H, dddd, 11.7, 11.7, 3.7, 3.7 Hz, H-2*α*), 1.79 (1H, ov, H-3*α*), 1.38 (1H, ov, H-3*β*), 2.46 (1H, brt, H-4*β*), 1.52 (1H, ov, H-5*β*), 1.82 (1H, ov, H-6*α*), 1.58 (1H, ov, H-6*β*), 1.48 (1H, ov, H-7*β*), 1.74 (1H, ov, H-7*α*), 0.98 (1H, brd, H-9*β*), 1.39 (1H, ov, H-11*β*), 1.53 (1H, ov, H-11*α*), 1.51 (1H, ov, H-12*β*), 1.45 (1H, ov, H-12*α*), 2.69 (1H, brt, H-13), 2.29 (1H, ddd, 12.7, 3.7, 1.7 Hz, H-14*α*), 1.36 (1H, ov, H-14*β*), 3.77 (1H, brs, H-15*β*), 5.17 (1H, brs, H-17a), 5.03 (1H, brs, H-17b), 0.94 (3H, s, CH_3_-20), 3.16 (2H, m, CH_2_-1′), 1.20–1.42 (2H, ov, CH_2_-2′), 1.24 (10H, ov, CH_2_-3′,-4′,-5′,-6′,-7′), 0.83 (3H, t, 7.3 Hz, CH_3_-8′) (Figure S21); ^13^C-NMR (CDCl_3_-*d*_1_, 62.5 MHz) *δ* 49.03 (C-1), 64.04 (C-2), 37.74 (C-3), 45.24 (C-4), 48.72 (C-5), 25.93 (C-6), 34.73 (C-7), 47.36 (C-8), 52.65 (C-9), 40.27 (C-10), 17.97 (C-11), 32.19 (C-12), 42.06 (C-13), 36.01 (C-14), 82.37 (C-15), 159.68 (C-16), 108.39 (C-17), 174.36 (C-19), 16.40 (C-20), 39.55 (C-1′), 29.43 (C-2′), 27.03 (C-3′), 29.13 (C-4′), 29.13 (C-5′), 31.64 (C-6′), 22.49 (C-7′), 13.95 (C-8′) (Figure S22); HRESIMS *m*/*z* 432.3498 [M + H]^+^ (calcd. for C_27_H_45_NO_3_, 432.3472) (Figure S23).

- Compound **11**.

The crude mixture was purified on a silica gel column eluting with CH_2_Cl_2_-MeOH (98:2) to afford a fraction containing compound **11** (27.2 mg, 90.97%) as an amorphous yellow solid; [*α*${]}_{D}^{25}$–305.92 (*c* 0.10, CHCl_3_); ^1^H-NMR (CDCl_3_-*d*_1_, 250 MHz) *δ* 0.66 (1H, dd, 11.7, 11.7 Hz, H-1*β*), 2.14 (1H, ddd, 11.7, 3.7, 1.7 Hz, H-1*α*), 4.36 (1H, dddd, 11.7, 11.7, 3.7, 3.7 Hz, H-2*α*), 1.79 (1H, ov, H-3*α*), 1.36 (1H, ov, H-3*β*), 2.45 (1H, brt, H-4*β*), 1.52 (1H, ov, H-5*β*), 1.80 (1H, ov, H-6*α*), 1.59 (1H, ov, H-6*β*), 1.48 (1H, ov, H-7*β*), 1.73 (1H, ov, H-7*α*), 0.97 (1H, brd, H-9*β*), 1.38 (1H, ov, H-11*β*), 1.57 (1H, ov, H-11*α*), 1.52 (1H, ov, H-12*β*), 1.45 (1H, ov, H-12*α*), 2.68 (1H, brt, H-13), 2.25 (1H, ddd, 12.7, 3.7, 1.7 Hz, H-14*α*), 1.36 (1H, ov, H-14*β*), 3.76 (1H, brs, H-15*β*), 5.16 (1H, brs, H-17a), 5.02 (1H, brs, H-17b), 0.93 (3H, s, CH_3_-20), 3.14 (2H, ov, CH_2_-1′), 1.21–1.42 (2H, ov, CH_2_-2′), 1.22 (14H, ov, CH_2_-3′,-4′,-5′,-6′,-7′,-8′,-9′), 0.82 (3H, t, 7.3 Hz, CH_3_-10′) (Figure S24); ^13^C-NMR (CDCl_3_-*d*_1_, 62.5 MHz) *δ* 48.97 (C-1), 63.93 (C-2), 37.65 (C-3), 45.18 (C-4), 48.70 (C-5), 25.86 (C-6), 34.71 (C-7), 47.32 (C-8), 52.64 (C-9), 40.22 (C-10), 17.95 (C-11), 32.16 (C-12), 42.03 (C-13), 35.99 (C-14), 82.30 (C-15), 159.56 (C-16), 108.35 (C-17), 174.20 (C-19), 16.37 (C-20), 39.53 (C-1′), 31.70 (C-2′), 27.02 (C-3′), 29.13 (C-4′), 29.39 (C-5′), 29.39 (C-6′), 29.16 (C-7′), 29.46 (C-8′), 22.49 (C-9′), 13.93 (C-10′) (Figure S25); HRESIMS *m*/*z* 460.3812 [M + H]^+^ (calcd. for C_29_H_49_NO_3_, 460.3785) (Figure S26).

- Compound **12**.

The crude mixture was purified on a silica gel column eluting with CH_2_Cl_2_-MeOH (98:2) to afford a fraction containing compound **12** (19.8 mg, 88.39%) as an amorphous yellow solid; [*α*${]}_{D}^{25}$–111.37 (*c* 0.10, CHCl_3_); ^1^H-NMR (CDCl_3_-*d*_1_, 250 MHz) *δ* 0.66 (1H, dd, 11.7, 11.7 Hz, H-1*β*), 2.16 (1H, ddd, 11.7, 3.7, 1.7 Hz, H-1*α*), 4.39 (1H, dddd, 11.7, 11.7, 3.7, 3.7 Hz, H-2*α*), 1.87 (1H, ov, H-3*α*), 1.32 (1H, ov, H-3*β*), 2.43 (1H, brt, H-4*β*), 1.38 (1H, ov, H-5*β*), 1.78 (1H, ov, H-6*α*), 1.59 (1H, ov, H-6*β*), 1.43 (1H, ov, H-7*β*), 1.71 (1H, ov, H-7*α*), 1.00 (1H, brd, H-9*β*), 1.42 (1H, ov, H-11*β*), 1.55 (1H, ov, H-11*α*), 1.58 (1H, ov, H-12*β*), 1.44 (1H, ov, H-12*α*), 2.65 (1H, brt, H-13), 2.27 (1H, ddd, 12.7, 3.7, 1.7 Hz, H-14*α*), 1.38 (1H, ov, H-14*β*), 3.77 (1H, brs, H-15*β*), 5.17 (1H, brs, H-17a), 5.03 (1H, brs, H-17b), 0.95 (3H, s, CH_3_-20), 4.00 (1H, m, CH-1′), 1.10 (3H, d, 6.6 Hz, CH_3_-2′), 1.08 (3H, d, 6.6 Hz, CH_3_-3′) (Figure S27); ^13^C-NMR (CDCl_3_-*d*_1_, 62.5 MHz) *δ* 48.94 (C-1), 63.98 (C-2), 37.62 (C-3), 45.17 (C-4), 48.68 (C-5), 25.88 (C-6), 34.71 (C-7), 47.31 (C-8), 52.65 (C-9), 40.27 (C-10), 17.95 (C-11), 32.15 (C-12), 42.02 (C-13), 35.98 (C-14), 82.34 (C-15), 159.60 (C-16), 108.40 (C-17), 173.30 (C-19), 16.36 (C-20), 41.25 (C-1′), 22.68 (C-2′), 22.46 (C-3′) (Figure S28); HRESIMS *m*/*z* 362.2704 [M + H]^+^ (calcd. for C_22_H_35_NO_3_, 362.2690) (Figure S29).

- Compound **13**.

The crude mixture was purified on a silica gel column eluting with CH_2_Cl_2_-MeOH (98:2) to afford a fraction containing compound **13** (21.6 mg, 92.62%) as an amorphous yellow solid; [*α*${]}_{D}^{25}$–111.42 (*c* 0.10, CHCl_3_); ^1^H-NMR (CDCl_3_, 250 MHz) *δ* 0.68 (1H, dd, 11.7, 11.7 Hz, H-1*β*), 2.18 (1H, ddd, 11.7, 3.7, 1.7 Hz, H-1*α*), 4.41 (1H, dddd, 11.7, 11.7, 3.7, 3.7 Hz, H-2*α*), 1.85 (1H, ov, H-3*α*), 1.35 (1H, ov, H-3*β*), 2.48 (1H, ov, H-4*β*), 1.40 (1H, ov, H-5*β*), 1.77 (1H, ov, H-6*α*), 1.57 (1H, ov, H-6*β*), 1.74 (1H, ov, H-7*β*), 1.45 (1H, ov, H-7*α*), 1.03 (1H, brd, H-9*β*), 1.37 (1H, ov, H-11*β*), 1.53 (1H, ov, H-11*α*), 1.53 (1H, ov, H-12*β*), 1.43 (1H, ov, H-12*α*), 2.71 (1H, brt, H-13), 2.30 (1H, ddd, 12.7, 3.7, 1.7 Hz, H-14*α*), 1.35 (1H, ov, H-14*β*), 3.79 (1H, brs, H-15*β*), 5.18 (1H, brs, H-17a), 5.04 (1H, brs, H-17b), 0.96 (3H, s, CH_3_-20), 3.02 (2H, m, CH-1′), 2.48 (1H, ov, CH-2′), 0.90 (3H, d, 6.6 Hz, CH_3_-3′), 0.87 (3H, d, 6.6 Hz, CH_3_-4′) (Figure S30); ^13^C-NMR (CDCl_3_, 62.5 MHz) *δ* 49.05 (C-1), 64.10 (C-2), 37.82 (C-3), 45.39 (C-4), 48.73 (C-5), 25.95 (C-6), 34.75 (C-7), 47.38 (C-8), 52.65 (C-9), 40.27 (C-10), 17.98 (C-11), 32.17 (C-12), 42.06 (C-13), 35.99 (C-14), 82.40 (C-15), 159.69 (C-16), 108.43 (C-17), 174.29 (C-19), 16.47 (C-20), 47.06 (C-1′), 28.27 (C-2′), 20.25 (C-3′), 20.22 (C-4′) (Figure S31); HRESIMS *m*/*z* 376.2864 [M + H]^+^ (calcd. for C_23_H_37_NO_3_, 376.2846) (Figure S32).

- Compound **14**.

The crude mixture was purified on a silica gel column eluting with CH_2_Cl_2_-MeOH (98:2) to afford a fraction containing compound **14** (25.4 mg, 96.10%) as an amorphous yellow solid; [*α*${]}_{D}^{25}$–28.41 (*c* 0.10, CHCl_3_); ^1^H-NMR (CDCl_3_-*d*_1_, 250 MHz) *δ* 0.70 (1H, dd, 11.7, 11.7 Hz, H-1*β*), 2.33 (1H, ddd, 11.7, 3.7, 1.7 Hz, H-1*α*), 4.47 (1H, dddd, 11.7, 11.7, 3.7, 3.7 Hz, H-2*α*), 1.85 (1H, ov, H-3*α*), 1.34 (1H, ov, H-3*β*), 2.73 (1H, ov, H-4*β*), 1.76 (1H, ov, H-5*β*), 1.58 (1H, ov, H-6*α*), 1.59 (1H, ov, H-6*β*), 1.43 (1H, ov, H-7*β*), 1.56 (1H, ov, H-7*α*), 1.06 (1H, brd, H-9*β*), 1.37 (1H, ov, H-11*β*), 1.56 (1H, ov, H-11*α*), 1.40 (1H, ov, H-12*β*), 1.35 (1H, ov, H-12*α*), 2.47 (1H, brt, H-13), 2.21 (1H, ddd, 12.7, 3.7, 1.7 Hz, H-14*α*), 1.36 (1H, ov, H-14*β*), 3.81 (1H, brs, H-15*β*), 5.21 (1H, brs, H-17a), 5.07 (1H, brs, H-17b), 1.01 (3H, s, CH_3_-20), 4.07 (1H, m, CH-1′), 1.41 (2H, ov, CH_2_-2′), 1.25 (1H, brd, CH-3′), 0.88 (3H, d, 6.6 Hz, CH_3_-4′), 0.89 (3H, d, 6.6 Hz, CH_3_-5′), 1.10 (3H, d, 6.6 Hz, CH_3_-6′) (Figure S33); ^13^C-NMR (CDCl_3_-*d*_1_, 62.5 MHz) *δ* 49.04 (C-1), 64.29 (C-2), 37.86 (C-3), 45.33 (C-4), 48.75 (C-5), 26.08 (C-6), 34.77 (C-7), 47.41 (C-8), 52.69 (C-9), 40.38 (C-10), 18.01 (C-11), 32.21 (C-12), 42.10 (C-13), 36.06 (C-14), 82.49 (C-15), 159.81 (C-16), 108.49 (C-17), 173.31 (C-19), 16.46 (C-20), 43.43 (C-1′), 46.20 (C-2′), 25.03 (C-3′), 22.59 (C-4′), 22.52 (C-5′), 21.39 (C-6′) (Figure S34); HRESIMS *m*/*z* 404.3186 [M + H]^+^ (calcd. for C_25_H_41_NO_3_, 404.3159) (Figure S35).

- Compound **15**.

The crude mixture was purified on a silica gel column eluting with CH_2_Cl_2_-MeOH (98:2) to afford a fraction containing compound **15** (24.5 mg, 93.15%) as an amorphous yellow solid; [*α*${]}_{D}^{25}$–58.69 (*c* 0.10, CHCl_3_); ^1^H-NMR (CDCl_3_-*d*_1_, 250 MHz) *δ* 0.71 (1H, dd, 11.7, 11.7 Hz, H-1*β*), 2.33 (1H, ddd, 11.7, 3.7, 1.7 Hz, H-1*α*), 4.45 (1H, dddd, 11.7, 11.7, 3.7, 3.7 Hz, H-2*α*), 1.85 (1H, ov, H-3*α*), 1.33 (1H, ov, H-3*β*), 2.74 (1H, ov, H-4*β*), 1.78 (1H, ov, H-5*β*), 1.60 (1H, ov, H-6*α*), 1.58 (1H, ov, H-6*β*), 1.45 (1H, ov, H-7*β*), 1.57 (1H, ov, H-7*α*), 1.04 (1H, brd, H-9*β*), 1.38 (1H, ov, H-11*β*), 1.57 (1H, ov, H-11*α*), 1.41 (1H, ov, H-12*β*), 1.34 (1H, ov, H-12*α*), 2.47 (1H, brt, H-13), 2.22 (1H, ddd, 12.7, 3.7, 1.7 Hz, H-14*α*), 1.34 (1H, ov, H-14*β*), 3.82 (1H, brs, H-15*β*), 5.21 (1H, brs, H-17a), 5.08 (1H, brs, H-17b), 1.01 (3H, s, CH_3_-20), 4.02 (1H, m, CH-1′), 1.44 (2H, ov, CH_2_-2′), 1.61 (1H, brd, CH-3′), 0.90 (3H, d, 6.6 Hz, CH_3_-4′), 0.90 (3H, d, 6.6 Hz, CH_3_-5′), 1.10 (3H, d, 6.6 Hz, CH_3_-6′) (Figure S36); ^13^C-NMR (CDCl_3_-*d*_1_, 62.5 MHz) *δ* 49.12 (C-1), 64.34 (C-2), 37.89 (C-3), 45.57 (C-4), 48.85 (C-5), 26.04 (C-6), 34.77 (C-7), 47.44 (C-8), 52.62 (C-9), 40.10 (C-10), 18.02 (C-11), 32.20 (C-12), 42.12 (C-13), 35.96 (C-14), 82.52 (C-15), 159.86 (C-16), 108.49 (C-17), 173.33 (C-19), 16.55 (C-20), 43.59 (C-1′), 46.17 (C-2′), 25.17 (C-3′), 22.63 (C-4′), 22.35 (C-5′), 21.28 (C-6′) (Figure S37); HRESIMS *m*/*z* 404.3186 [M + H]^+^ (calcd. for C_25_H_41_NO_3_, 404.3159) (Figure S38).

- Compound **16**.

The crude mixture was purified on a silica gel column eluting with CH_2_Cl_2_-MeOH (98:2) to afford a fraction containing compound **16** (23.7 mg, 95.18%) as an amorphous yellow solid; [*α*${]}_{D}^{25}$–117.91 (*c* 0.10, CHCl_3_); ^1^H-NMR (CDCl_3_-*d*_1_, 250 MHz) *δ* 0.64 (1H, dd, 11.7, 11.7 Hz, H-1*β*), 2.16 (1H, ddd, 11.7, 3.7, 1.7 Hz, H-1*α*), 4.36 (1H, dddd, 11.7, 11.7, 3.7, 3.7 Hz, H-2*α*), 1.80 (1H, ov, H-3*α*), 1.34 (1H, ov, H-3*β*), 2.43 (1H, m, H-4*β*), 1.43 (1H, ov, H-5*β*), 1.68 (1H, ov, H-6*α*), 1.56 (1H, ov, H-6*β*), 1.39 (1H, ov, H-7*β*), 1.61 (1H, ov, H-7*α*), 1.03 (1H, brd, H-9*β*), 1.38 (1H, ov, H-11*β*), 1.54 (1H, ov, H-11*α*), 1.63 (1H, ov, H-12*β*), 1.53 (1H, ov, H-12*α*), 2.66 (1H, brt, H-13), 2.25 (1H, ddd, 12.7, 3.7, 1.7 Hz, H-14*α*), 1.43 (1H, ov, H-14*β*), 3.75 (1H, brs, H-15*β*), 5.15 (1H, brs, H-17a), 5.00 (1H, brs, H-17b), 0.93 (3H, s, CH_3_-20), 3.69 (1H, m, CH-1′), 1.20–1.80 (10H, ov, CH_2_-2′,-3′,-4′,-5′,-6′) (Figure S39); ^13^C-NMR (CDCl_3_-*d*_1_, 62.5 MHz) *δ* 48.64 (C-1), 63.92 (C-2), 37.62 (C-3), 45.12 (C-4), 48.98 (C-5), 25.34 (C-6), 34.68 (C-7), 47.27 (C-8), 52.63 (C-9), 40.23 (C-10), 17.91 (C-11), 32.11 (C-12), 41.98 (C-13), 36.34 (C-14), 82.26 (C-15), 159.54 (C-16), 108.29 (C-17), 173.08 (C-19), 16.34 (C-20), 48.06 (C-1′), 33.03 (C-2′), 24.68 (C-3′), 25.83 (C-4′), 24.75 (C-5′), 32.76 (C-6′) (Figure S40); HRESIMS *m*/*z* 402.3018 [M + H]^+^ (calcd. for C_25_H_39_NO_3_, 402.3003) (Figure S41).

- Compound **17**.

The crude mixture was purified on a silica gel column eluting with CH_2_Cl_2_-MeOH (97:3) to afford a fraction containing compound **17** (21.7 mg, 82.50%) as an amorphous yellow–orange solid; [*α*${]}_{D}^{25}$–78.18 (*c* 0.10, CHCl_3_); ^1^H-NMR (CDCl_3_-*d*_1_, 250 MHz) *δ* 0.65 (1H, dd, 11.7, 11.7 Hz, H-1*β*), 2.14 (1H, ddd, 11.7, 3.7, 1.7 Hz, H-1*α*), 4.37 (1H, dddd, 11.7, 11.7, 3.7, 3.7 Hz, H-2*α*), 1.74 (1H, ov, H-3*α*), 1.33 (1H, ov, H-3*β*), 2.41 (1H, m, H-4*β*), 1.48 (1H, ov, H-5*β*), 1.58 (1H, ov, H-6*α*), 1.60 (1H, ov, H-6*β*), 1.48 (1H, ov, H-7*β*), 1.56 (1H, ov, H-7*α*), 0.98 (1H, brd, H-9*β*), 1.38 (1H, ov, H-11*β*), 1.57 (1H, ov, H-11*α*), 1.44 (1H, ov, H-12*β*), 1.45 (1H, ov, H-12*α*), 2.71 (1H, brt, H-13), 2.31 (1H, ddd, 12.7, 3.7, 1.7 Hz, H-14*α*), 1.36 (1H, ov, H-14*β*), 3.76 (1H, brs, H-15*β*), 5.18 (1H, brs, H-17a), 5.05 (1H, brs, H-17b), 0.87 (3H, s, CH_3_-20), 3.50 (2H, m, CH_2_-1′), 2.80 (2H, m, CH_2_-2′) 7.20–7.27 (5H, m, CH-4′,-5′,-6′,-7′,-8′) (Figure S42); ^13^C-NMR (CDCl_3_-*d*_1_, 62.5 MHz) *δ* 48.93 (C-1), 63.99 (C-2), 37.64 (C-3), 45.13 (C-4), 48.54 (C-5), 25.65 (C-6), 34.63 (C-7), 47.31 (C-8), 52.57 (C-9), 40.19 (C-10), 17.93 (C-11), 32.16 (C-12), 42.03 (C-13), 36.13 (C-14), 82.32 (C-15), 159.65 (C-16), 108.37 (C-17), 174.35 (C-19), 16.29 (C-20), 40.41 (C-1′), 35.13 (C-2′), 138.70 (C-3′), 128.52 (C-4′), 128.56 (C-5′), 126.47 (C-6′), 128.56 (C-7′), 128.52 (C-8′) (Figure S43); HRESIMS *m*/*z* 424.2864 [M + H]^+^ (calcd. for C_27_H_37_NO_3_, 424.2846) (Figure S44).

- Compound **18**.

The crude mixture was purified on a silica gel column eluting with CH_2_Cl_2_-MeOH (97:3) to afford a fraction containing compound **18** (22.8 mg, 86.69%) as an amorphous yellow–orange solid; [*α*${]}_{D}^{25}$–78.80 (*c* 0.10, CHCl_3_); ^1^H-NMR (DMSO-*d*_6_, 250 MHz) *δ* 0.66 (1H, dd, 11.7, 11.7 Hz, H-1*β*), 2.17 (1H, ddd, 11.7, 3.7, 1.7 Hz, H-1*α*), 4.41 (1H, dddd, 11.7, 11.7, 3.7, 3.7 Hz, H-2*α*), 1.80 (1H, ov, H-3*α*), 1.33 (1H, ov, H-3*β*), 2.69 (1H, m, H-4*β*), 1.65 (1H, ov, H-5*β*), 1.62 (1H, ov, H-6*α*), 1.59 (1H, ov, H-6*β*), 1.48 (1H, ov, H-7*β*), 1.56 (1H, ov, H-7*α*), 1.02 (1H, brd, H-9*β*), 1.32 (1H, ov, H-11*β*), 1.57 (1H, ov, H-11*α*), 1.43 (1H, ov, H-12*β*), 1.34 (1H, ov, H-12*α*), 2.62 (1H, brt, H-13), 2.19 (1H, ddd, 12.7, 3.7, 1.7 Hz, H-14*α*), 1.36 (1H, ov, H-14*β*), 3.74 (1H, brs, H-15*β*) 5.18 (1H, brs, H-17a), 5.07 (1H, brs, H-17b), 0.96 (3H, s, CH_3_-20), 4.29 (2H, m, CH_2_-1′), 7.10–7.17 (4H, m, CH-3′,-4′,-6′,-7′), 2.30 (3H, s, CH_3_-8′) (Figure S45); ^13^C-NMR (DMSO-*d*_6_, 62.5 MHz) *δ* 50.46 (C-1), 65.34 (C-2), 38.55 (C-3), 46.66 (C-4), 50.32 (C-5), 26.24 (C-6), 36.18 (C-7), 48.78 (C-8), 54.67 (C-9), 43.70 (C-10), 19.26 (C-11), 33.61 (C-12), 44.13 (C-13), 37.23 (C-14), 83.67 (C-15), 160.35 (C-16), 109.21 (C-17), 176.79 (C-19), 17.51 (C-20), 41.56 (C-1′), 137.41 (C-2′), 129.00 (C-3′), 130.12 (C-4′), 137.90 (C-5′), 130.12 (C-6′), 129.00 (C-7′), 21.33 (C-8′) (Figure S46); HRESIMS *m*/*z* 424.2867 [M + H]^+^ (calcd. for C_27_H_37_NO_3_, 424.2846) (Figure S47).

### 3.4. General Procedure for the Synthesis of Di-Oxidates of Atractyligenin Amides (***19***–***32***)

**Figure 9:**
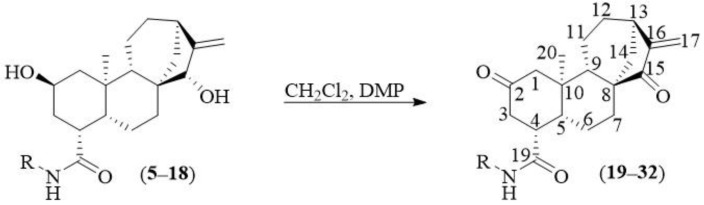
General procedure for the synthesis of di-oxi-amides **19**–**32**.

The amides (**5**–**18**) (1.0 equiv.) were dissolved in a small amount of CH_2_Cl_2_ (2 mL) in a two-necked flask previously filled with N_2_ and added dropwise over a period of 10 min (0 °C) to a homogeneous mixture of DMP (2.3 equiv.) in CH_2_Cl_2_ (Figure 9). The reaction mixture was stirred at r.t. (≈1 h) until it was quenched with saturated aqueous Na_2_S_2_O_3_ (2 mL), and the resulting mixture was stirred vigorously until it became clear. The mixture was then poured into EtOAc (10 mL), and the organic phase was washed twice with 10% aq. Na_2_S_2_O_3_/aq. NaHCO_3_ (1:1 mixture, 8 mL) brine (8 mL), then dried with Na_2_SO_4_. Removal of the solvent under vacuum afforded the di-oxidates of atractyligenin amides (**19**–**32**), which were purified by silica gel column chromatography.

- Compound **19**.

The crude mixture was purified on a silica gel column eluting with CH_2_Cl_2_-MeOH (99:1 → 98:2) to afford a fraction containing compound **19** (11.2 mg, 75.47%) as a white solid; [*α*${]}_{D}^{25}$–125.98 (*c* 0.10, CHCl_3_); ^1^H-NMR (CDCl_3_-*d*_1_, 250 MHz) *δ* 1.85 (1H, ov, H-1*β*), 2.55 (1H, ov, H-1*α*), 2.69 (1H, m, H-3*α*), 2.94 (1H, m, H-3*β*), 3.07 (1H, m, H-4*β*), 2.01 (1H, ov, H-5*β*), 1.88 (1H, ov, H-6*α*), 1.85 (1H, ov, H-6*β*), 1.97 (1H, ov, H-7*β*), 1.40 (1H, ov, H-7*α*), 1.61 (1H, ov, H-9*β*), 1.43 (1H, ov, H-11*β*), 1.49 (1H, ov, H-11*α*), 1.62 (1H, ov, H-12*β*), 1.45 (1H, ov, H-12*α*), 2.67 (1H, m, H-13), 2.32 (1H, d, 12.1 Hz, H-14*α*), 1.39 (1H, ov, H-14*β*), 5.96 (1H, brs, H-17a), 5.28 (1H, brs, H-17b), 1.03 (3H, s, CH_3_), 3.17 (2H, m, CH_2_-1′), 1.51-1.28 (2H, ov, CH_2_-2′), 0.91 (3H, t, 7.3 Hz, CH_3_-3′) (Figure S48); ^13^C-NMR (CDCl_3_-*d*_1_, 62.5 MHz) *δ* 55.35 (C-1), 209.78 (C-2), 43.97 (C-3), 47.21 (C-4), 48.15 (C-5), 24.56 (C-6), 32.96 (C-7), 51.74 (C-8), 50.64 (C-9), 44.13 (C-10), 18.10 (C-11), 31.66 (C-12), 37.81 (C-13), 35.92 (C-14), 209.78 (C-15), 148.87 (C-16), 115.15 (C-17), 172.24 (C-19), 16.95 (C-20), 41.50 (C-1′), 22.42 (C-2′), 11.44 (C-3′) (Figure S49); HRESIMS *m*/*z* 358.2389 [M + H]^+^ (calcd. for C_22_H_31_NO_3_, 358.2377) (Figure S50).

- Compound **20**.

The crude mixture was purified on a silica gel column eluting with CH_2_Cl_2_-MeOH (99:1 → 98:2) to afford a fraction containing compound **20** (12.4 mg, 81.36%) as a white solid; [*α*${]}_{D}^{25}$–127.00 (*c* 0.10, CHCl_3_); ^1^H-NMR (CDCl_3_-*d*_1_, 250 MHz) *δ* 1.84 (1H, ov, H-1*β*), 2.56 (1H, ov, H-1*α*), 2.62 (1H, ov, H-3*α*), 2.95 (1H, m, H-3*β*), 3.07 (1H, m, H-4*β*), 2.01 (1H, ov, H-5*β*), 1.83 (1H, ov, H-6*α*), 1.83 (1H, ov, H-6*β*), 1.99 (1H, ov, H-7*β*), 1.42 (1H, ov, H-7*α*), 1.53 (1H, ov, H-9*β*), 1.42 (1H, ov, H-11*β*), 1.50 (1H, ov, H-11*α*), 1.58 (1H, ov, H-12*β*), 1.44 (1H, ov, H-12*α*), 2.70 (1H, m, H-13), 2.33 (1H, d, 12.1 Hz, H-14*α*), 1.39 (1H, ov, H-14*β*), 5.96 (1H, brs, H-17a), 5.29 (1H, brs, H-17b), 1.06 (3H, s, CH_3_), 3.20 (2H, m, CH_2_-1′), 1.46–1.25 (2H, ov, CH_2_-2′), 1.33–1.25 (2H, ov, CH_2_-3′), 0.92 (3H, t, 7.3 Hz, CH_3_-4′) (Figure S51); ^13^C-NMR (CDCl_3_-*d*_1_, 62.5 MHz) *δ* 55.38 (C-1), 209.85 (C-2), 44.04 (C-3), 47.23 (C-4), 48.25 (C-5), 24.59 (C-6), 32.98 (C-7), 51.75 (C-8), 50.65 (C-9), 44.22 (C-10), 18.11 (C-11), 31.68 (C-12), 37.83 (C-13), 35.93 (C-14), 209.79 (C-15), 148.88 (C-16), 115.17 (C-17), 172.19 (C-19), 16.92 (C-20), 39.50 (C-1′), 31.20 (C-2′), 20.15 (C-3′), 13.68 (C-4′) (Figure S52); HRESIMS *m*/*z* 372.2549 [M + H]^+^ (calcd. for C_23_H_33_NO_3_, 372.2533) (Figure S53).

- Compound **21**.

The crude mixture was purified on a silica gel column eluting with CH_2_Cl_2_-MeOH (99:1 → 98:2) to afford a fraction containing compound **21** (14.3 mg, 85.47%) as a white solid; [*α*${]}_{D}^{25}$–90.56 (*c* 0.10, CHCl_3_); ^1^H-NMR (CDCl_3_-*d*_1_, 250 MHz) *δ* 1.83 (1H, ov, H-1*β*), 2.57 (1H, ov, H-1*α*), 2.69 (1H, ov, H-3*α*), 2.93 (1H, ov, H-3*β*), 3.03 (1H, m, H-4*β*), 2.00 (1H, ov, H-5*β*), 1.85 (1H, ov, H-6*α*), 1.96 (1H, ov, H-6*β*), 1.89 (1H, ov, H-7*β*), 1.44 (1H, ov, H-7*α*), 1.46 (1H, ov, H-9*β*), 1.35 (1H, ov, H-11*β*), 1.52 (1H, ov, H-11*α*), 1.69 (1H, ov, H-12*β*), 1.50 (1H, ov, H-12*α*), 2.69 (1H, m, H-13), 2.29 (1H, d, 12.1 Hz, H-14*α*), 1.39 (1H, ov, H-14*β*), 5.92 (1H, brs, H-17a), 5.26 (1H, brs, H-17b), 1.03 (3H, s, CH_3_), 3.13 (2H, m, CH_2_-1′), 1.46–1.25 (2H, ov, CH_2_-2′), 1.34–1.24 (2H, ov, CH_2_-3′), 1.24 (2H, ov, CH_2_-4′), 0.85 (3H, t, 7.3 Hz, CH_3_-5′) (Figure S54); ^13^C-NMR (CDCl_3_-*d*_1_, 62.5 MHz) *δ* 55.19 (C-1), 209.78 (C-2), 43.74 (C-3), 46.99 (C-4), 47.82 (C-5), 24.38 (C-6), 32.82 (C-7), 51.63 (C-8), 50.52 (C-9), 43.86 (C-10), 17.99 (C-11), 31.55 (C-12), 37.69 (C-13), 35.80 (C-14), 209.71 (C-15), 148.76 (C-16), 115.11 (C-17), 172.29 (C-19), 16.89 (C-20), 39.65 (C-1′), 29.02 (C-2′), 28.75 (C-3′), 22.16 (C-4′), 13.87 (C-5′) (Figure S55); HRESIMS *m*/*z* 386.2698 [M + H]^+^ (calcd. for C_24_H_35_NO_3_, 386.2690) (Figure S56).

- Compound **22**.

The crude mixture was purified on a silica gel column eluting with CH_2_Cl_2_-MeOH (99:1 → 98:2) to afford a fraction containing compound **22** (13.9 mg, 84.70%) as a white solid; [*α*${]}_{D}^{25}$–85.00 (*c* 0.10, CHCl_3_); ^1^H-NMR (CDCl_3_-*d*_1_, 250 MHz) *δ* 1.84 (1H, ov, H-1*β*), 2.61 (1H, ov, H-1*α*), 2.69 (1H, ov, H-3*α*), 2.94 (1H, ov, H-3*β*), 3.07 (1H, m, H-4*β*), 2.00 (1H, ov, H-5*β*), 1.87 (1H, ov, H-6*α*), 1.97 (1H, ov, H-6*β*), 1.76 (1H, ov, H-7*β*), 1.41 (1H, ov, H-7*α*), 1.44 (1H, ov, H-9*β*), 1.31 (1H, ov, H-11*β*), 1.48 (1H, ov, H-11*α*), 1.67 (1H, ov, H-12*β*), 1.53 (1H, ov, H-12*α*), 2.67 (1H, m, H-13), 2.32 (1H, d, 12.1 Hz, H-14*α*), 1.38 (1H, ov, H-14*β*), 5.96 (1H, brs, H-17a), 5.29 (1H, brs, H-17b), 1.06 (3H, s, CH_3_), 3.19 (2H, m, CH_2_-1′), 1.46–1.28 (2H, ov, CH_2_-2′), 1.33–1.28 (6H, ov, CH_2_-3′,-4′,-5′), 0.88 (3H, t, 7.3 Hz, CH_3_-6′) (Figure S57); ^13^C-NMR (CDCl_3_-*d*_1_, 62.5 MHz) *δ* 55.36 (C-1), 209.84 (C-2), 44.00 (C-3), 47.21 (C-4), 48.17 (C-5), 24.60 (C-6), 32.96 (C-7), 51.75 (C-8), 50.64 (C-9), 44.17 (C-10), 18.10 (C-11), 31.67 (C-12), 37.81 (C-13), 35.92 (C-14), 209.84 (C-15), 148.86 (C-16), 115.20 (C-17), 172.22 (C-19), 16.94 (C-20), 39.80 (C-1′), 30.08 (C-2′), 26.64 (C-3′), 31.38 (C-4′), 22.50 (C-5′), 13.97 (C-6′) (Figure S58); HRESIMS *m*/*z* 400.2861 [M + H]^+^ (calcd. for C_25_H_37_NO_3_, 400.2846) (Figure S59).

- Compound **23**.

The crude mixture was purified on a silica gel column eluting with CH_2_Cl_2_-MeOH (99:1 → 98:2) to afford a fraction containing compound **23** (14.2 mg, 83.58%) as a white solid; [*α*${]}_{D}^{25}$–89.98 (*c* 0.10, CHCl_3_); ^1^H-NMR (CDCl_3_-*d*_1_, 250 MHz) *δ* 1.96 (1H, ov, H-1*β*), 2.63 (1H, ov, H-1*α*), 2.65 (1H, ov, H-3*α*), 2.92 (1H, m, H-3*β*), 3.03 (1H, m, H-4*β*), 1.96 (1H, ov, H-5*β*), 1.88 (1H, ov, H-6*α*), 1.92 (1H, ov, H-6*β*), 1.67 (1H, ov, H-7*β*), 1.22 (1H, ov, H-7*α*), 1.44 (1H, ov, H-9*β*), 1.30 (1H, ov, H-11*β*), 1.50 (1H, ov, H-11*α*), 1.65 (1H, ov, H-12*β*), 1.63 (1H, ov, H-12*α*), 2.63 (1H, m, H-13), 2.27 (1H, d, 12.1 Hz, H-14*α*), 1.38 (1H, ov, H-14*β*), 5.91 (1H, brs, H-17a), 5.25 (1H, brs, H-17b), 1.03 (3H, s, CH_3_), 3.15 (2H, m, CH_2_-1′), 1.38 (2H, ov, CH_2_-2′), 1.22 (8H, ov, CH_2_-3′,-4′,-5′,-6′), 0.82 (3H, t, 7.3 Hz, CH_3_-7′) (Figure S60); ^13^C-NMR (CDCl_3_-*d*_1_, 62.5 MHz) *δ* 55.24 (C-1), 209.91 (C-2), 43.78 (C-3), 47.08 (C-4), 47.91 (C-5), 24.45 (C-6), 32.87 (C-7), 51.69 (C-8), 50.58 (C-9), 43.94 (C-10), 18.04 (C-11), 31.77 (C-12), 37.76 (C-13), 35.86 (C-14), 209.83 (C-15), 148.81 (C-16), 115.19 (C-17), 172.43 (C-19), 16.92 (C-20), 39.80 (C-1′), 28.80 (C-2′), 26.88 (C-3′), 31.63 (C-4′), 22.46 (C-5′), 22.46 (C-6′), 13.96 (C-7′) (Figure S61); HRESIMS *m*/*z* 414.3015 [M + H]^+^ (calcd. for C_26_H_39_NO_3_, 414.3003) (Figure S62).

- Compound **24**.

The crude mixture was purified on a silica gel column eluting with CH_2_Cl_2_-MeOH (99:1 → 98:2) to afford a fraction containing compound **24** (15.6 mg, 88.84%) as a white solid; [*α*${]}_{D}^{25}$–107.26 (*c* 0.10, CHCl_3_); ^1^H-NMR (CDCl_3_-*d*_1_, 250 MHz) *δ* 1.96 (1H, ov, H-1*β*), 2.63 (1H, ov, H-1*α*), 2.64 (1H, ov, H-3*α*), 2.92 (1H, ov, H-3*β*), 3.01 (1H, ov, H-4*β*), 2.01 (1H, ov, H-5*β*), 1.82 (1H, ov, H-6*α*), 1.96 (1H, ov, H-6*β*), 1.65 (1H, ov, H-7*β*), 1.25 (1H, ov, H-7*α*), 1.44 (1H, ov, H-9*β*), 1.31 (1H, ov, H-11*β*), 1.52 (1H, ov, H-11*α*), 1.66 (1H, ov, H-12*β*), 1.62 (1H, ov, H-12*α*), 2.63 (1H, m, H-13), 2.27 (1H, ov, H-14*α*), 1.39 (1H, ov, H-14*β*), 5.89 (1H, brs, H-17a), 5.23 (1H, brs, H-17b), 1.02 (3H, s, CH_3_), 3.13 (2H, m, CH_2_-1′), 1.41–1.20 (2H, ov, CH_2_-2′), 1.37–1.20 (10H, ov, CH_2_-3′,-4′,-5′,-6′,-7′), 0.80 (3H, t, 7.3 Hz, CH_3_-8′) (Figure S63); ^13^C-NMR (CDCl_3_-*d*_1_, 62.5 MHz) *δ* 55.09 (C-1), 209.73 (C-2), 43.57 (C-3), 46.84 (C-4), 47.62 (C-5), 24.21 (C-6), 32.74 (C-7), 51.56 (C-8), 50.44 (C-9), 43.67 (C-10), 17.92 (C-11), 31.49 (C-12), 37.62 (C-13), 35.73 (C-14), 209.66 (C-15), 148.71 (C-16), 115.01 (C-17), 172.37 (C-19), 16.88 (C-20), 39.63 (C-1′), 29.01 (C-2′), 26.84 (C-3′), 31.55 (C-4′), 26.84 (C-5′), 2×22.41 (C-6′,-7′), 13.90 (C-8′) (Figure S64); HRESIMS *m*/*z* 428.3176 [M + H]^+^ (calcd. for C_27_H_41_NO_3_, 428.3159) (Figure S65).

- Compound **25**.

The crude mixture was purified on a silica gel column eluting with CH_2_Cl_2_-MeOH (99:1 → 98:2) to afford a fraction containing compound **25** (16.2 mg, 86.58%) as a white solid; [*α*${]}_{D}^{25}$–86.04 (*c* 0.10, CHCl_3_); ^1^H-NMR (CDCl_3_-*d*_1_, 250 MHz) *δ* 1.94 (1H, ov, H-1*β*), 2.57 (1H, ov, H-1*α*), 2.67 (1H, ov, H-3*α*), 2.53 (1H, ov, H-3*β*), 2.93 (1H, ov, H-4*β*), 1.97 (1H, ov, H-5*β*), 1.85 (1H, ov, H-6*α*), 1.94 (1H, ov, H-6*β*), 1.68 (1H, ov, H-7*β*), 1.25 (1H, ov, H-7*α*), 1.44 (1H, ov, H-9*β*), 1.33 (1H, ov, H-11*β*), 1.51 (1H, ov, H-11*α*), 1.66 (1H, ov, H-12*β*), 1.59 (1H, ov, H-12*α*), 2.64 (1H, m, H-13), 2.29 (1H, d, 12.1 Hz, H-14*α*), 1.40 (1H, ov, H-14*β*), 5.92 (1H, brs, H-17a), 5.28 (1H, brs, H-17b), 1.03 (3H, s, CH_3_), 3.15 (2H, m, CH_2_-1′), 1.41–1.20 (2H, ov, CH_2_-2′), 1.37–1.22 (14H, ov, CH_2_-3′,-4′,-5′,-6′,-7′,-8′,-9′), 0.84 (3H, t, 7.3 Hz, CH_3_-10′) (Figure S66); ^13^C-NMR (CDCl_3_-*d*_1_, 62.5 MHz) *δ* 55.21 (C-1), 209.78 (C-2), 43.78 (C-3), 47.01 (C-4), 47.86 (C-5), 24.41 (C-6), 32.84 (C-7), 51.65 (C-8), 50.52 (C-9), 43.89 (C-10), 18.01 (C-11), 31.57 (C-12), 37.70 (C-13), 35.82 (C-14), 209.70 (C-15), 148.76 (C-16), 115.12 (C-17), 172.27 (C-19), 16.91 (C-20), 39.70 (C-1′), 31.75 (C-2′), 26.92 (C-3′), 29.09 (C-4′), 29.43 (C-5′), 29.17 (C-6′), 29.13 (C-7′), 31.75 (C-8′), 22.25 (C-9′), 14.00 (C-10′) (Figure S67); HRESIMS *m*/*z* 456.3489 [M + H]^+^ (calcd. for C_29_H_45_NO_3_, 456.3472) (Figure S68).

- Compound **26**.

The crude mixture was purified on a silica gel column eluting with CH_2_Cl_2_-MeOH (99:1 → 98:2) to afford a fraction containing compound **26** (11.9 mg, 81.06%) as a white solid; [*α*${]}_{D}^{25}$–179.46 (*c* 0.10, CHCl_3_); ^1^H-NMR (CDCl_3_-*d*_1_, 250 MHz) *δ* 1.96 (1H, ov, H-1*β*), 2.58 (1H, ov, H-1*α*), 2.67 (1H, ov, H-3*α*), 2.53 (1H, ov, H-3*β*), 2.90 (1H, m, H-4*β*), 1.99 (1H, ov, H-5*β*), 1.85 (1H, ov, H-6*α*), 1.94 (1H, ov, H-6*β*), 1.70 (1H, ov, H-7*β*), 1.23 (1H, ov, H-7*α*), 1.42 (1H, ov, H-9*β*), 1.36 (1H, ov, H-11*β*), 1.52 (1H, ov, H-11*α*), 1.67 (1H, ov, H-12*β*), 1.53 (1H, ov, H-12*α*), 2.65 (1H, ov, H-13), 2.30 (1H, d, 12.1 Hz, H-14*α*), 1.40 (1H, ov, H-14*β*), 5.95 (1H, brs, H-17a), 5.28 (1H, brs, H-17b), 1.06 (3H, s, CH_3_), 4.01 (1H, m, CH-1′), 1.12 (3H, d, 6.6 Hz, CH_3_-2′), 1.08 (3H, d, 6.6 Hz, CH_3_-3′) (Figure S69); ^13^C-NMR (CDCl_3_-*d*_1_, 62.5 MHz) *δ* 55.28 (C-1), 209.83 (C-2), 43.88 (C-3), 47.06 (C-4), 48.01 (C-5), 24.48 (C-6), 32.90 (C-7), 51.68 (C-8), 50.59 (C-9), 44.08 (C-10), 18.05 (C-11), 31.60 (C-12), 37.74 (C-13), 35.86 (C-14), 209.81 (C-15), 148.79 (C-16), 115.19 (C-17), 171.37 (C-19), 17.00 (C-20), 41.53 (C-1′), 22.65 (C-2′), 21.99 (C-3′) (Figure S70); HRESIMS *m*/*z* 358.2395 [M + H]^+^ (calcd. for C_22_H_31_NO_3_, 358.2376) (Figure S71).

- Compound **27**.

The crude mixture was purified on a silica gel column eluting with CH_2_Cl_2_-MeOH (99:1 → 98:2) to afford a fraction containing compound **27** (12.2 mg, 79.94%) as a white solid; [*α*${]}_{D}^{25}$–110.62 (*c* 0.10, CHCl_3_); ^1^H-NMR (CDCl_3_-*d*_1_, 250 MHz) *δ* 1.95 (1H, ov, H-1*β*), 2.56 (1H, ov, H-1*α*), 2.67 (1H, ov, H-3*α*), 2.54 (1H, ov, H-3*β*), 2.96 (1H, ov, H-4*β*), 1.99 (1H, ov, H-5*β*), 1.85 (1H, ov, H-6*α*), 1.91 (1H, ov, H-6*β*), 1.71 (1H, ov, H-7*β*), 1.21 (1H, ov, H-7*α*), 1.40 (1H, ov, H-9*β*), 1.36 (1H, ov, H-11*β*), 1.51 (1H, ov, H-11*α*), 1.67 (1H, ov, H-12*β*), 1.53 (1H, ov, H-12*α*), 2.65 (1H, m, H-13), 2.30 (1H, d, 12.1 Hz, H-14*α*), 1.40 (1H, ov, H-14*β*), 5.92 (1H, brs, H-17a), 5.26 (1H, brs, H-17b), 1.03 (3H, s, CH_3_), 3.01 (2H, m, CH_2_-1′), 1.71 (1H, ov, CH-2′), 0.87 (3H, d, 6.6 Hz, CH_3_-3′), 0.87 (3H, d, 6.6 Hz, CH_3_-4′) (Figure S72); ^13^C-NMR (CDCl_3_-*d*_1_, 62.5 MHz) *δ* 55.23 (C-1), 209.76 (C-2), 43.88 (C-3), 47.16 (C-4), 47.96 (C-5), 24.40 (C-6), 32.87 (C-7), 51.66 (C-8), 50.53 (C-9), 43.99 (C-10), 18.01 (C-11), 31.56 (C-12), 37.71 (C-13), 35.82 (C-14), 209.76 (C-15), 148.78 (C-16), 115.09 (C-17), 172.40 (C-19), 16.91 (C-20), 47.18 (C-1′), 28.01 (C-2′), 20.17 (C-3′), 20.17 (C-4′) (Figure S73); HRESIMS *m*/*z* 372.2555 [M + H]^+^ (calcd. for C_23_H_33_NO_3_, 372.2533) (Figure S74).

- Compound **28**.

The crude mixture was purified on a silica gel column eluting with CH_2_Cl_2_-MeOH (99:1 → 98:2) to afford a fraction containing compound **28** (13.8 mg, 84.09%) as a white solid; [*α*${]}_{D}^{25}$–19.34 (*c* 0.10, CHCl_3_); ^1^H-NMR (CDCl_3_-*d*_1_, 250 MHz) *δ* 2.00 (1H, ov, H-1*β*), 2.53 (1H, ov, H-1*α*), 2.70 (1H, ov, H-3*α*), 2.55 (1H, ov, H-3*β*), 2.91 (1H, m, H-4*β*), 2.02 (1H, ov, H-5*β*), 1.85 (1H, ov, H-6*α*), 1.93 (1H, ov, H-6*β*), 1.71 (1H, ov, H-7*β*), 1.20 (1H, ov, H-7*α*), 1.44 (1H, ov, H-9*β*), 1.33 (1H, ov, H-11*β*), 1.53 (1H, ov, H-11*α*), 1.71 (1H, ov, H-12*β*), 1.58 (1H, ov, H-12*α*), 2.68 (1H, m, H-13), 2.34 (1H, d, 12.1 Hz, H-14*α*), 1.42 (1H, ov, H-14*β*), 5.97 (1H, brs, H-17a), 5.29 (1H, brs, H-17b), 1.09 (3H, s, CH_3_), 3.98 (1H, m, CH-1′), 1.44 (2H, ov, CH_2_-2′), 1.28 (1H, d, 6.6 Hz, CH-3′), 0.89 (3H, d, 6.6 Hz, CH_3_-4′), 0.89 (3H, d, 6.6 Hz, CH_3_-5′), 1.12 (3H, d, 6.6 Hz, CH_3_-6′) (Figure S75); ^13^C-NMR (CDCl_3_-*d*_1_, 62.5 MHz) *δ* 55.35 (C-1), 209.77 (C-2), 43.83 (C-3), 47.24 (C-4), 48.13 (C-5), 24.66 (C-6), 33.00 (C-7), 51.75 (C-8), 50.67 (C-9), 44.09 (C-10), 18.11 (C-11), 31.68 (C-12), 37.83 (C-13), 35.96 (C-14), 209.48 (C-15), 148.89 (C-16), 115.17 (C-17), 171.45 (C-19), 17.05 (C-20), 45.74 (C-1′), 43.99 (C-2′), 24.93 (C-3′), 22.54 (C-4′), 22.49 (C-5′), 21.21 (C-6′) (Figure S76); HRESIMS *m*/*z* 400.2875 [M + H]^+^ (calcd. for C_25_H_37_NO_3_, 400.2846) (Figure S77).

- Compound **29**.

The crude mixture was purified on a silica gel column eluting with CH_2_Cl_2_-MeOH (99:1 → 98:2) to afford a fraction containing compound **29** (14.0 mg, 85.31%) as a white solid; [*α*${]}_{D}^{25}$–32.60 (*c* 0.10, CHCl_3_); ^1^H-NMR (CDCl_3_-*d*_1_, 250 MHz) *δ* 2.02 (1H, ov, H-1*β*), 2.57 (1H, ov, H-1*α*), 2.68 (1H, ov, H-3*α*), 2.61 (1H, ov, H-3*β*), 2.94 (1H, m, H-4*β*), 1.97 (1H, ov, H-5*β*), 1.89 (1H, ov, H-6*α*), 1.93 (1H, ov, H-6*β*), 1.70 (1H, ov, H-7*β*), 1.23 (1H, ov, H-7*α*), 1.44 (1H, ov, H-9*β*), 1.38 (1H, ov, H-11*β*), 1.55 (1H, ov, H-11*α*), 1.66 (1H, ov, H-12*β*), 1.50 (1H, ov, H-12*α*), 2.64 (1H, m, H-13), 2.32 (1H, d, 12.1 Hz, H-14*α*), 1.38 (1H, ov, H-14*β*), 5.97 (1H, brs, H-17a), 5.29 (1H, brs, H-17b), 1.08 (3H, s, CH_3_), 4.02 (1H, m, CH-1′), 1.43 (2H, ov, CH_2_-2′), 1.24 (1H, d, 6.6 Hz, CH-3′), 0.91 (3H, d, 6.6 Hz, CH_3_-4′), 0.91 (3H, d, 6.6 Hz, CH_3_-5′), 1.06 (3H, d, 6.6 Hz, CH_3_-6′) (Figure S78); ^13^C-NMR (CDCl_3_-*d*_1_, 62.5 MHz) *δ* 55.45 (C-1), 209.99 (C-2), 43.64 (C-3), 47.38 (C-4), 48.46 (C-5), 24.63 (C-6), 33.04 (C-7), 51.79 (C-8), 50.65 (C-9), 44.49 (C-10), 18.14 (C-11), 31.69 (C-12), 37.85 (C-13), 35.93 (C-14), 209.81 (C-15), 148.91 (C-16), 115.16 (C-17), 171.30 (C-19), 17.11 (C-20), 46.23 (C-1′), 44.27 (C-2′), 25.15 (C-3′), 22.65 (C-4′), 22.49 (C-5′), 20.54 (C-6′) (Figure S79); HRESIMS *m*/*z* 400.2861 [M + H]^+^ (calcd. for C_25_H_37_NO_3_, 400.2846) (Figure S80).

- Compound **30**.

The crude mixture was purified on a silica gel column eluting with CH_2_Cl_2_-MeOH (99:1 → 98:2) to afford a fraction containing compound **30** (14.3 mg, 87.62%) as a white solid; [*α*${]}_{D}^{25}$–139.14 (*c* 0.10, CHCl_3_); ^1^H-NMR (CDCl_3_-*d*_1_, 250 MHz) *δ* 2.00 (1H, d, 13.4 Hz, H-1*β*), 2.55 (1H, dd, 13.4, 2.1 Hz, H-1*α*), 2.68 (1H, m, H-3*α*), 2.58 (1H, brt, H-3*β*), 2.91 (1H, m, H-4*β*), 2.02 (1H, ov, H-5*β*), 1.86 (1H, ov, H-6*α*), 1.96 (1H, ov, H-6*β*), 1.70 (1H, ov, H-7*β*), 1.21 (1H, ov, H-7*α*), 1.42 (1H, ov, H-9*β*), 1.39 (1H, ov, H-11*β*), 1.53 (1H, ov, H-11*α*), 1.67 (1H, ov, H-12*β*), 1.85 (1H, ov, H-12*α*), 3.05 (1H, brdd, H-13), 2.31 (1H, brd, H-14*α*), 1.42 (1H, ov, H-14*β*), 5.95 (1H, brs, H-17a), 5.28 (1H, brs, H-17b), 1.06 (3H, s, CH_3_), 3.71 (1H, m, CH-1′), 1.20–1.65 (10H, ov, CH_2_-2′,-3′,-4′,-5′,-6′) (Figure S81); ^13^C-NMR (CDCl_3_-*d*_1_, 62.5 MHz) *δ* 55.29 (C-1), 209.80 (C-2), 43.93 (C-3), 47.11 (C-4), 48.23 (C-5), 24.63 (C-6), 32.31 (C-7), 51.69 (C-8), 50.60 (C-9), 44.10 (C-10), 18.05 (C-11), 31.61 (C-12), 37.74 (C-13), 35.85 (C-14), 209.72 (C-15), 148.81 (C-16), 115.16 (C-17), 171.22 (C-19), 17.04 (C-20), 48.02 (C-1′), 32.92 (C-2′), 24.66 (C-3′), 25.41 (C-4′), 24.49 (C-5′), 32.95 (C-6′) (Figure S82); HRESIMS *m*/*z* 398.2707 [M + H]^+^ (calcd. for C_25_H_35_NO_3_, 398.2690) (Figure S83).

- Compound **31**.

The crude mixture was purified on a silica gel column eluting with CH_2_Cl_2_-MeOH (99:1 → 98:2) to afford a fraction containing compound **31** (11.6 mg, 67.32%) as a white solid; [*α*${]}_{D}^{25}$–431.17 (*c* 0.10, CHCl_3_); ^1^H-NMR (CDCl_3_-*d*_1_, 250 MHz) *δ* 1.98 (1H, d, 13.4 Hz, H-1*β*), 2.44 (1H, dd, 13.4, 2.1 Hz, H-1*α*), 2.66 (1H, m, H-3*α*), 2.52 (1H, brt, H-3*β*), 2.87 (1H, m, H-4*β*), 2.02 (1H, ov, H-5*β*), 1.82 (1H, ov, H-6*α*), 1.94 (1H, ov, H-6*β*), 1.68 (1H, ov, H-7*β*), 1.28 (1H, ov, H-7*α*), 1.45 (1H, ov, H-9*β*), 1.37 (1H, ov, H-11*β*), 1.53 (1H, ov, H-11*α*), 1.67 (1H, ov, H-12*β*), 1.85 (1H, ov, H-12*α*), 3.06 (1H, brdd, H-13), 2.24 (1H, brd, H-14*α*), 1.42 (1H, ov, H-14*β*), 5.94 (1H, brs, H-17a), 5.28 (1H, brs, H-17b), 0.98 (3H, s, CH_3_), 3.44 (1H, dddd, 7.1, 7.1, 6.3, 6.3, CH-1a’) 3.71 (1H, dddd, 7.1, 7.1, 6.3, 6.3, CH-1b’), 2.80–2.87 (2H, m, CH_2_-2′) 7.21–7.29 (5H, m, CH-4′,-5′,-6′,-7′,-8′) (Figure S84); ^13^C-NMR (CDCl_3_-*d*_1_, 62.5 MHz) *δ* 55.07 (C-1), 209.69 (C-2), 43.43 (C-3), 46.85 (C-4), 47.40 (C-5), 24.23 (C-6), 32.72 (C-7), 51.57 (C-8), 50.45 (C-9), 43.46 (C-10), 17.93 (C-11), 31.53 (C-12), 37.67 (C-13), 35.88 (C-14), 209.12 (C-15), 148.74 (C-16), 115.08 (C-17), 172.56 (C-19), 16.85 (C-20), 40.66 (C-1′), 35.05 (C-2′), 138.60 (C-3′), 128.51 (C-4′), 128.54 (C-5′), 126.41 (C-6′), 128.54 (C-7′), 128.51 (C-8′) (Figure S85); HRESIMS *m*/*z* 420.2555 [M + H]^+^ (calcd. for C_27_H_33_NO_3_, 420.2533) (Figure S86).

- Compound **32**.

The crude mixture was purified on a silica gel column eluting with CH_2_Cl_2_-MeOH (99:1 → 98:2) to afford a fraction containing compound **32** (11.9 mg, 69.06%) as a white solid; [*α*${]}_{D}^{25}$–160.54 (*c* 0.10, CHCl_3_); ^1^H-NMR (CD_3_OD-*d*_4_, 250 MHz) *δ* 1.88 (1H, d, 13.4 Hz, H-1*β*), 2.18 (1H, dd, 13.4, 2.1 Hz, H-1*α*), 1.86 (1H, m, H-3*α*), 2.18 (1H, brt, H-3*β*), 2.65 (1H, m, H-4*β*), 1.80 (1H, ov, H-5*β*), 1.76 (1H, ov, H-6*α*), 1.71 (1H, ov, H-6*β*), 1.68 (1H, ov, H-7*β*), 1.22 (1H, ov, H-7*α*), 1.13 (1H, ov, H-9*β*), 1.37 (1H, ov, H-11*β*), 1.54 (1H, ov, H-11*α*), 1.67 (1H, ov, H-12*β*), 1.82 (1H, ov, H-12*α*), 3.05 (1H, brdd, H-13), 2.33 (1H, brd, H-14*α*), 1.34 (1H, ov, H-14*β*), 5.88 (1H, brs, H-17a), 5.32 (1H, brs, H-17b), 1.02 (3H, s, CH_3_), 4.43 (2H, m, CH_2_-1′), 7.10–7.18 (4H, m, CH-3′,-4′,-6′,-7′), 2.29 (3H, s, CH_3_-8′) (Figure S87); ^13^C-NMR (DMSO-*d*_6_, 62.5 MHz) *δ* 51.46 (C-1), 209.10 (C-2), 44.66 (C-3), 47.80 (C-4), 48.63 (C-5), 23.35 (C-6), 32.81 (C-7), 51.46 (C-8), 50.85 (C-9), 42.09 (C-10), 17.80 (C-11), 31.54 (C-12), 37.25 (C-13), 35.91 (C-14), 209.10 (C-15), 149.20 (C-16), 114.39 (C-17), 173.72 (C-19), 16.34 (C-20), 40.22 (C-1′), 136.92 (C-2′), 127.37 (C-3′), 128.69 (C-4′), 134.46 (C-5′), 128.69 (C-6′), 127.37 (C-7′), 20.67 (C-8′) (Figure S88); HRESIMS *m*/*z* 420.2552 [M + H]^+^ (calcd. for C_27_H_33_NO_3_, 420.2533) (Figure S89).

### 3.5. Cell Culture Conditions and Reagents

Colon cancer cells (HCT116 and Caco-2) were purchased from the Interlab Cell Line Collection (ICLC, Genoa, Italy) and cultured as monolayers in DMEM supplemented with 10% (*v*/*v*) heat-inactivated FCS and 2 mM glutamine in the presence of a 1% penicillin/streptomycin solution [52]. Differentiated Caco-2 cells, used as an enterocyte-like model [53,54], were prepared as previously reported following the Natoli procedure [46].

For the reported experiments, cells were seeded in 96-well microplates for MTT assay or 6-well plates for the other experimental conditions. Then, cells were incubated at 37 °C in a humidified atmosphere containing 5% CO_2_ for 24 h before proceeding with the treatment with the compounds or vehicle only. Stock solutions of amides or their di-oxidated derivatives were prepared in DMSO and stored at −20 °C until use. All working solutions were prepared in DMEM, never exceeding 0.01% (*v*/*v*) DMSO. The vehicle condition reported in each experiment as control was represented by untreated cells incubated in the presence of the corresponding DMSO volume. All cell culture media and culture reagents were provided by Euroclone SpA (Pero, Italy). All other reagents and chemicals, except where differently indicated, were purchased from Millipore Sigma (Milan, Italy).

### 3.6. Assessment of Cell Viability

The cytotoxic effects of analysed compounds were assessed by the colorimetric 3-(4,5-dimethylthiazol-2-yl)-2,5-diphenyltetrazolium bromide (MTT) assay. MTT was purchased from Millipore Sigma (Milan, Italy). For this assay, 8 × 10^3^ cells/well were plated in a 96-well plate and incubated with compounds for periods reported in the Results section. Then, 10 μL of MTT solution (5 mg/mL in PBS) of added to the cell medium, and the incubation was protracted for 2 h at 37 °C in the dark. MTT is metabolised to formazan salt in viable cells [55]. Afterwards, the media were taken out, and cells were lysed in lysis buffer (20% SDS and 10% dimethylformamide). The cell viability was determined by analysing the absorbance of the formazan read at 490 nm with 630 nm as a reference wavelength using an automatic ELISA plate reader (OPSYS MR, Dynex Technologies, Chantilly, VA, USA). IC_50_ values were determined by Graphpad Prism 7.0 software (San Diego, CA, USA).

### 3.7. Analysis of Apoptotic Cell Death by Vital Hoechst Staining

Apoptotic cell death was assessed by vital Hoechst 33342 staining [56] (Invitrogen; Thermo Fisher Scientific, Inc., Waltham, MA, USA) according to vendor’s specifications. Morphological analysis of condensed or fragmented chromatin was estimated by a fluorescence microscope and pictures were taken with Leica Q Fluoro software (Leica Microsystems, Wetzlar, Germany; https://www.leica-microsystems.com/it, (accessed on 21 March 2024)).

### 3.8. Western Blotting Analysis

Whole-cell extracts prepared in ice-cold lysis RIPA buffer (1% NP-40, 0.5% sodium deoxycholate and 0.1% SDS in PBS, pH 7.4) and supplemented with a protease inhibitor cocktail were subjected to SDS-PAGE and consequent immunoblot. PARP-1 antibody (cat. no. sc-53643) was provided by Santa Cruz Biotechnology Inc. (Santa Cruz, CA, USA), while anti-caspase-3 was from Cell Signaling Technology (cat. no. #9662; Cell Signaling Technology Inc., Beverly, MA, USA). In all performed experiments, the analysed proteins were normalised with *γ*-tubulin (cat. no. T3559; Sigma-Aldrich, Milan, Italy) used as loading control. In all analyses, protein bands were detected with an ECL™ Prime Western Blotting System (Cytiva, Merck KGaA, Milan, Italy) using a ChemiDoc XRS System equipped with Quantity One software 4.6.6 (Bio-Rad Laboratories, Inc., Hercules, CA, USA).

### 3.9. Statistics

All data and statistics were analysed by GraphPad PrismTM 7.0 software (Graph PadPrismTM Software Inc., San Diego, CA, USA). Data were reported as the mean ± S.E. Comparisons between the control (untreated) vs. treated samples were analysed by applying Student’s *t*-test and one-way analysis of variance.

## 4. Conclusions

In conclusion, 36 new derivatives were synthesized starting from the natural diterpene atractyligenin. The different designated compounds were evaluated as potential anticancer agents. Overall, the obtained results provide evidence that all new compounds are endowed with an antitumor potential. In particular, this research highlights that derivatives bearing keto functionalities at C-2 and C-15 are more effective than atractyligenin amides. The presence of a linear, medium-sized aliphatic chain appears to be another important structural requisite to obtain higher cytotoxicity. However, more interestingly, such synthetized molecules can selectively target colon cancer cells with respect to colon-like polarized cells. The study of the mechanism of action also revealed that the induction of apoptotic cell death could be at the root of the antitumour efficacy of di-oxidated derivatives of diterpene atractyligenin amides on colon cancer cells. However, in light of the impact of p53 status on cytotoxicity and anticancer activity, since p53 is frequently dysregulated in colon cancer [57,58], our future investigations will be focused on the role of p53 of colon cancer cells treated with di-oxidated derivatives of diterpene atractyligenin amides. For these studies, the two colon cancer cell lines used in this research represent ideal models that fit the scope well, since HCT116 cells possess wild-type p53 [52], while Caco-2 cells are p53-null.

## Figures and Tables

**Table 1:** IC_50_ values of di-oxidated derivates of atractyligenin amides on HCT116 colon cancer cells after 24 h of treatment.

Compound	IC_50_ (μM)	Compound	IC_50_ (μM)
**19**	6.79	26	8.14
**21**	11.06	28	n.d.
**22**	8.21	29	n.d.
**23**	7.44	30	9.47
**24**	5.35	31	6.37
**25**	5.50		

**Table 2:** Comparative analysis of IC_50_ values of some di-oxidated derivates of atractyligenin amides tested on HCT116 and Caco-2 colon cancer cells after 24 h of treatment.

Colon Cancer Cell	HCT116	Caco-2
Compound	IC_50_ (μM)	IC_50_ (μM)
**19**	6.79	11.19
**21**	11.06	8.68
**23**	7.44	4.45
**24**	5.35	5.27
**25**	5.50	14.92
**26**	8.14	5.50

## Data Availability

Data are contained within the article and Supplementary Materials.

## Supplementary Materials

The following supporting information can be downloaded at: https://www.mdpi.com/article/10.3390/ijms25073925/s1.
