# Supplementary material for: Synthetic Derivatives of Natural ent-Kaurane Atractyligenin Disclose Anticancer Properties in Colon Cancer Cells, Triggering Apoptotic Cell Demise
Source: Int J Mol Sci. 2024 Mar 31;25(7):3925. doi: 10.3390/ijms25073925 (PMC11011390; doi:10.3390/ijms25073925)
Supplement: Supplementary file 1 [file ijms-25-03925-s001.zip › ijms-2928404-supplementary.pdf]

Supplementary Materials

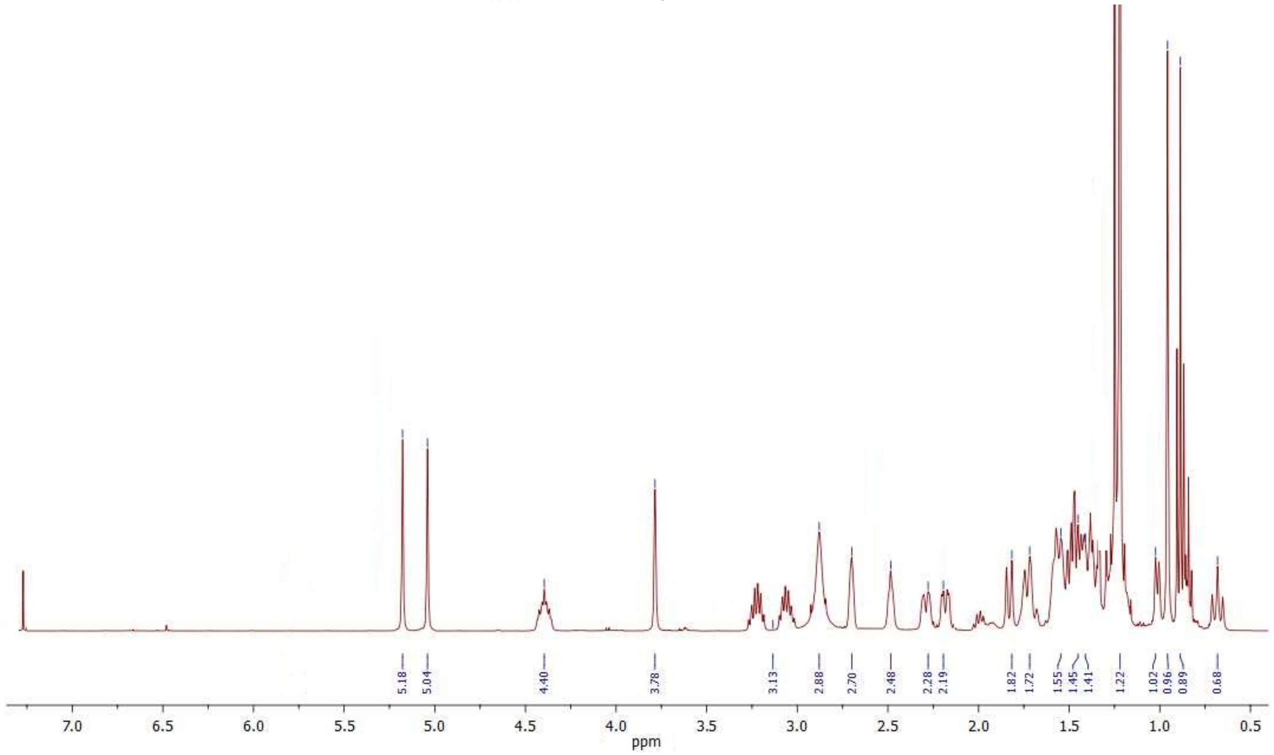

Figure S1. ^{1}H-NMR spectrum of compound **5**.

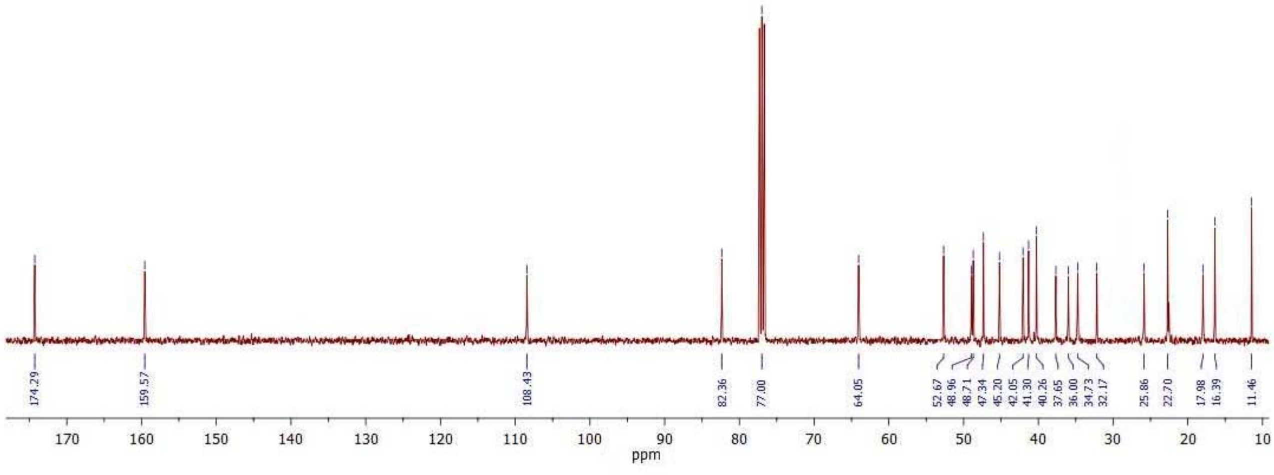

Figure S2. ^{13}C-NMR spectrum of compound **5**.

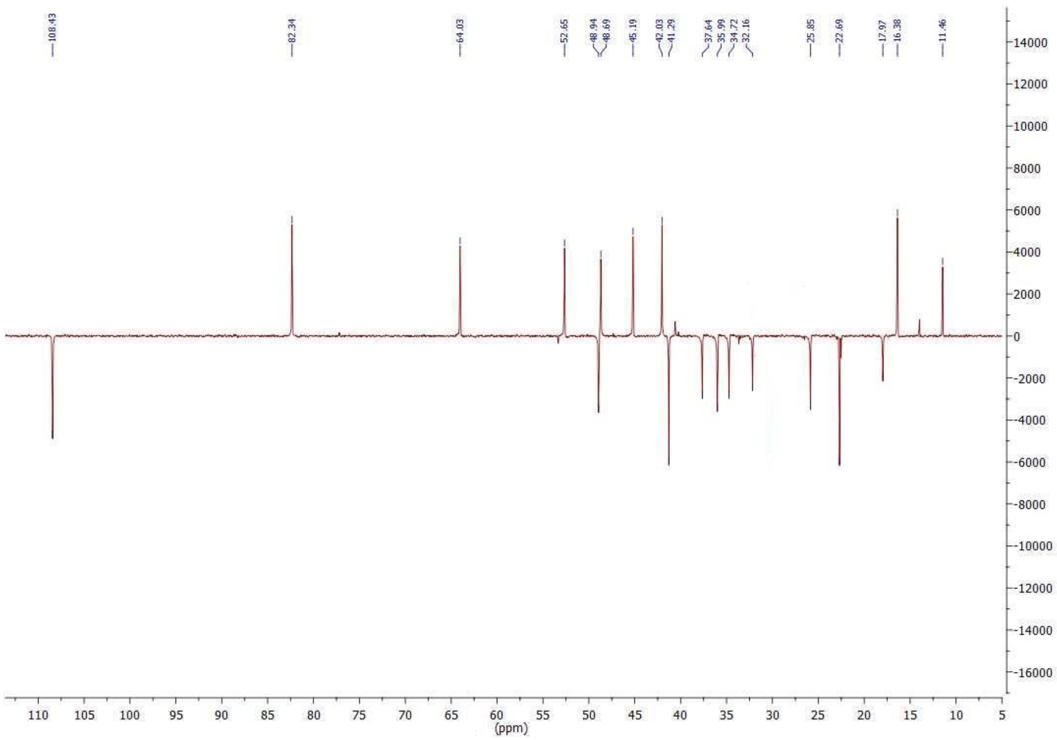

Figure S3. DEPT spectrum of compound **5**.

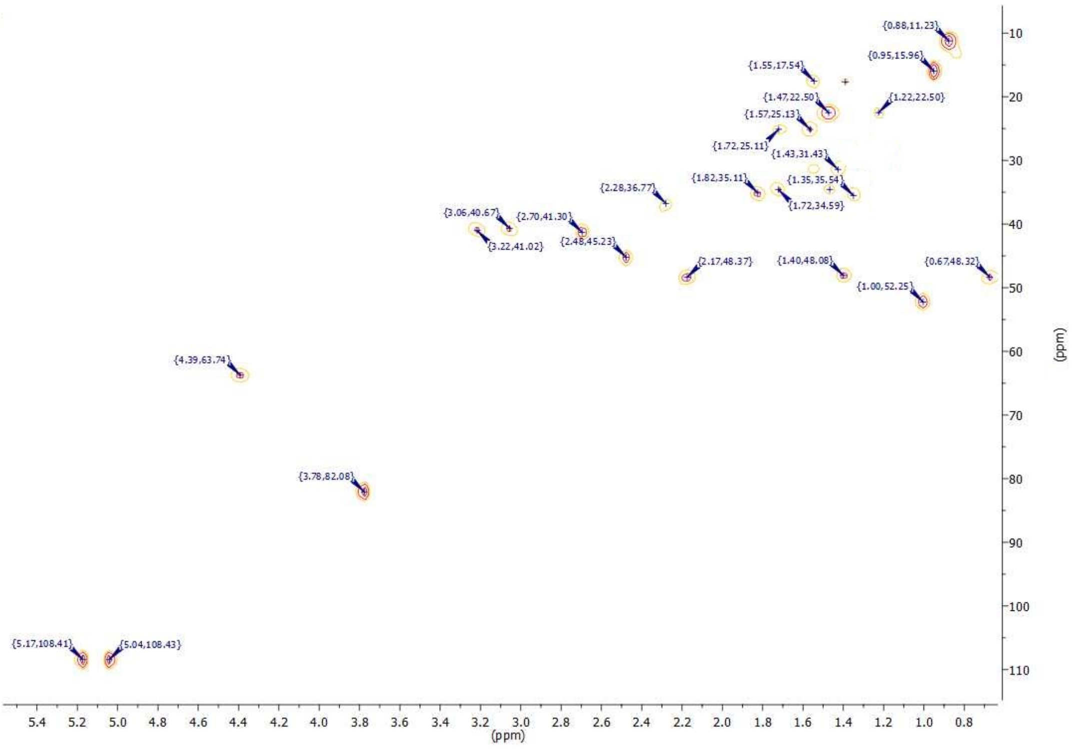

Figure S4. HSQC spectrum of compound **5**.

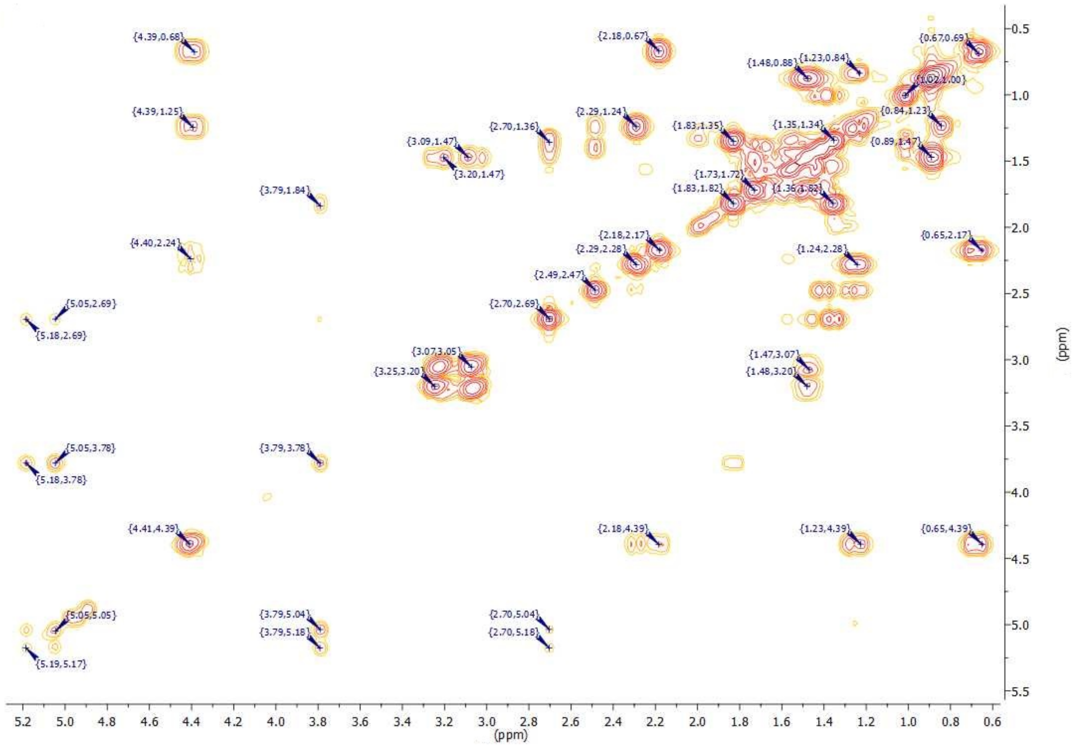

Figure S5. COSY spectrum of compound **5**.

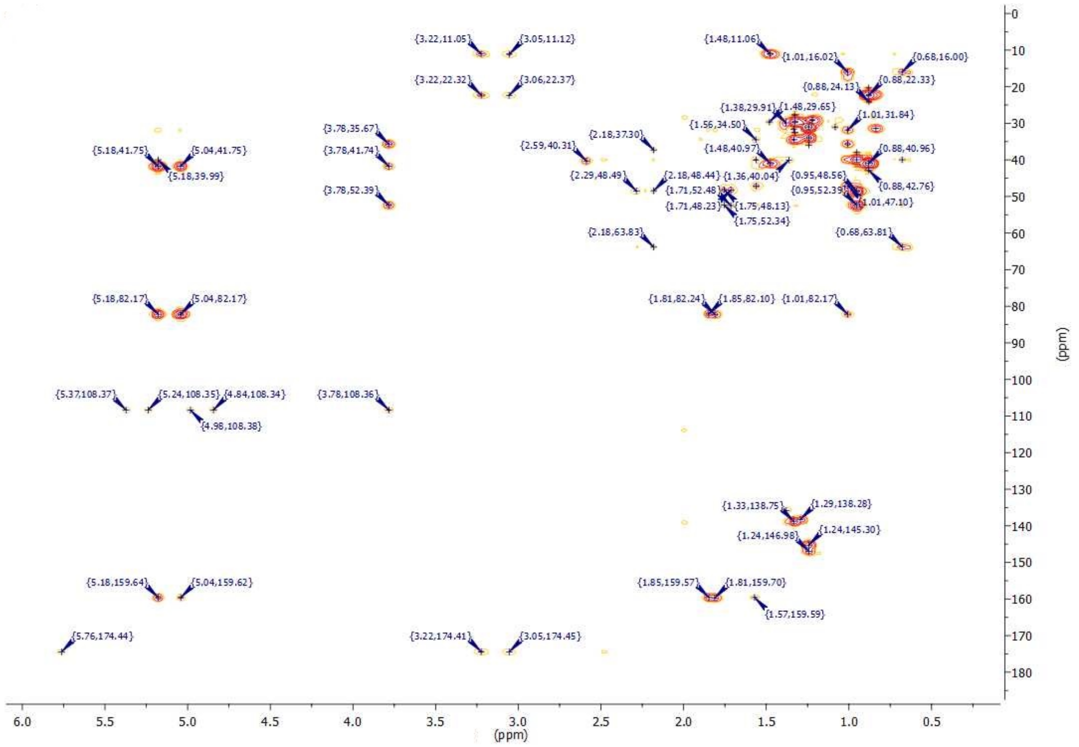

Figure S6. HMBC spectrum of compound **5**.

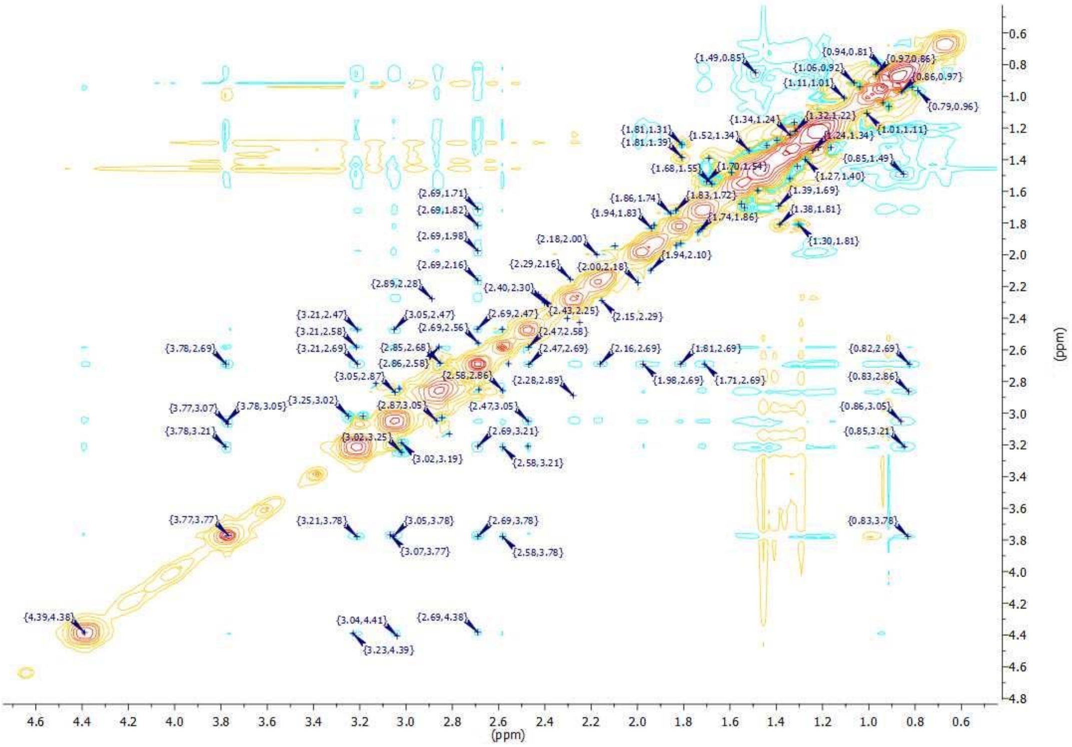

Figure S7. NOESY spectrum of compound **5**.

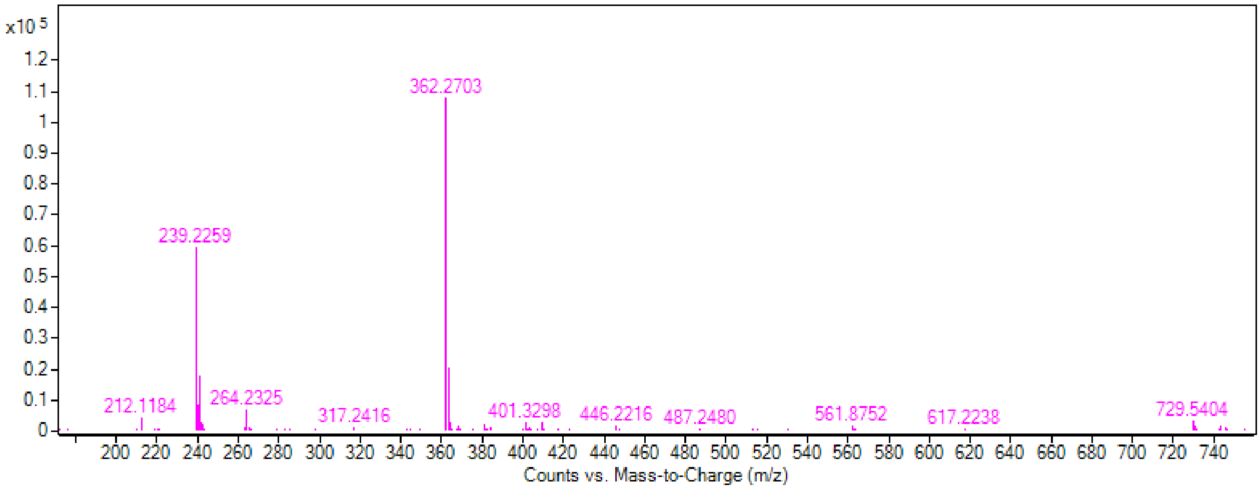

Figure S8. Mass spectrum of compound **5**.

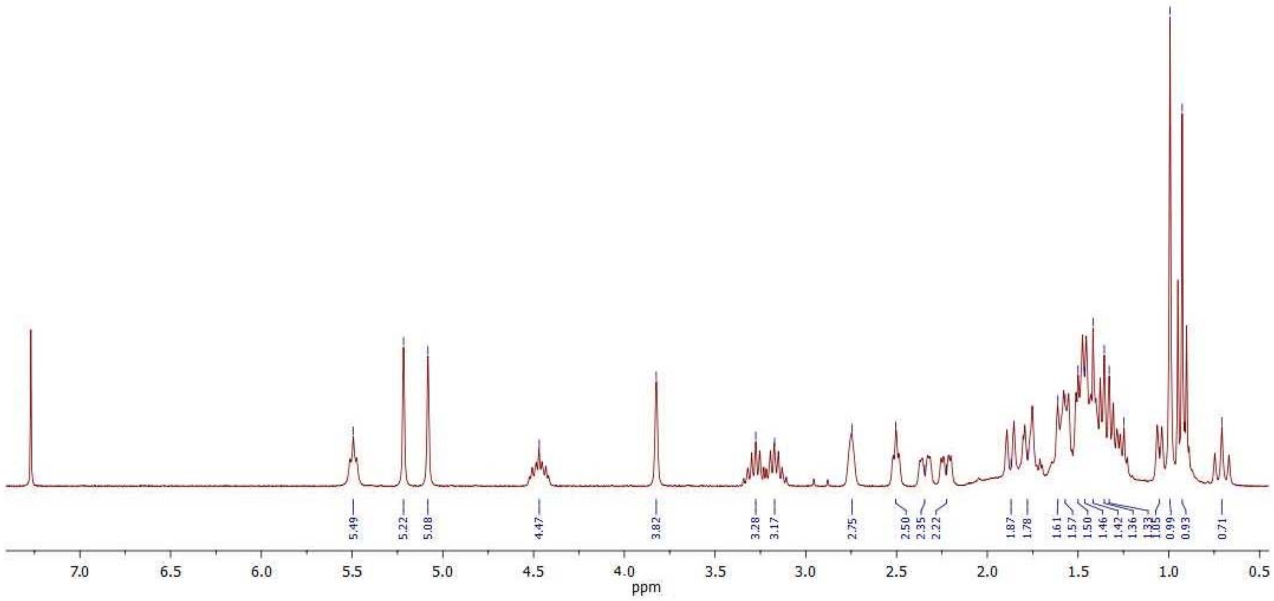

Figure S9. ^{1}H-NMR spectrum of compound **6**.

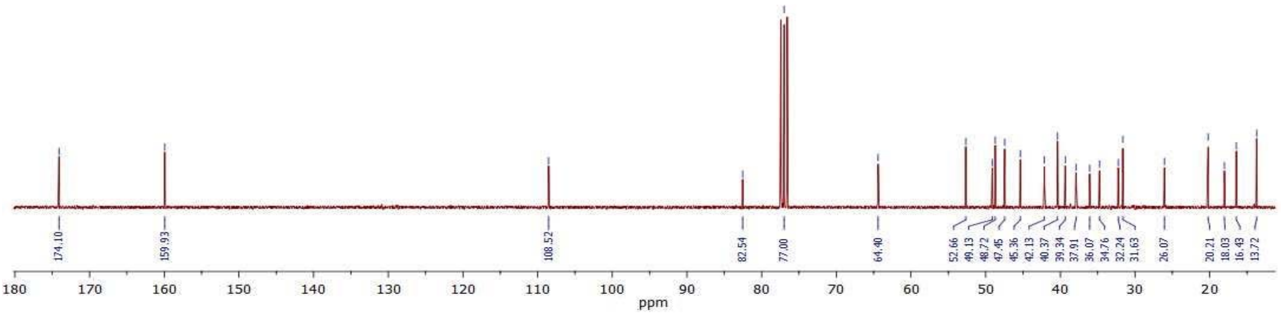

Figure S10. ^{13}C-NMR spectrum of compound **6**.

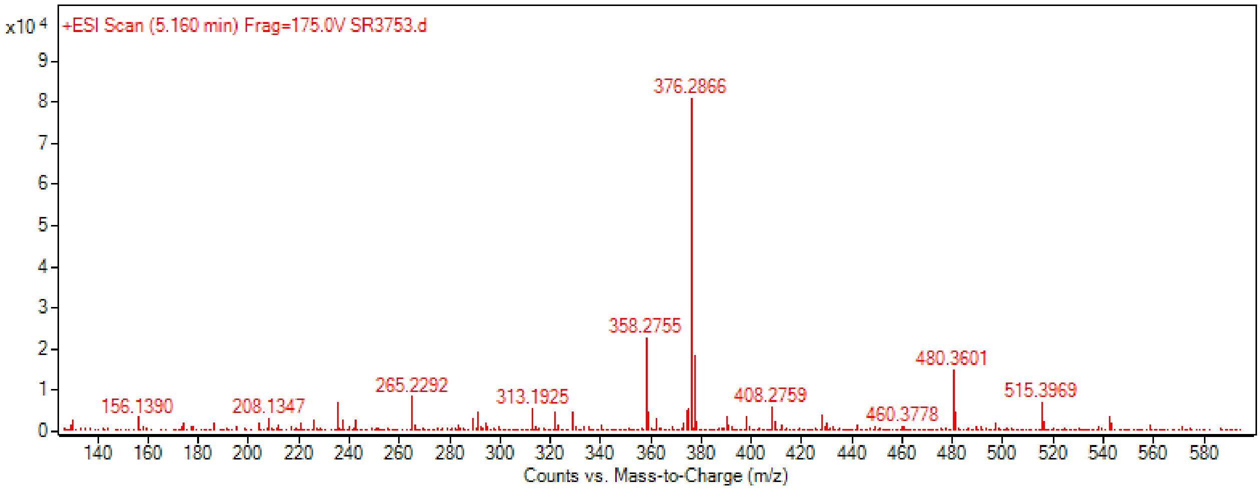

Figure S11. Mass spectrum of compound **6**.

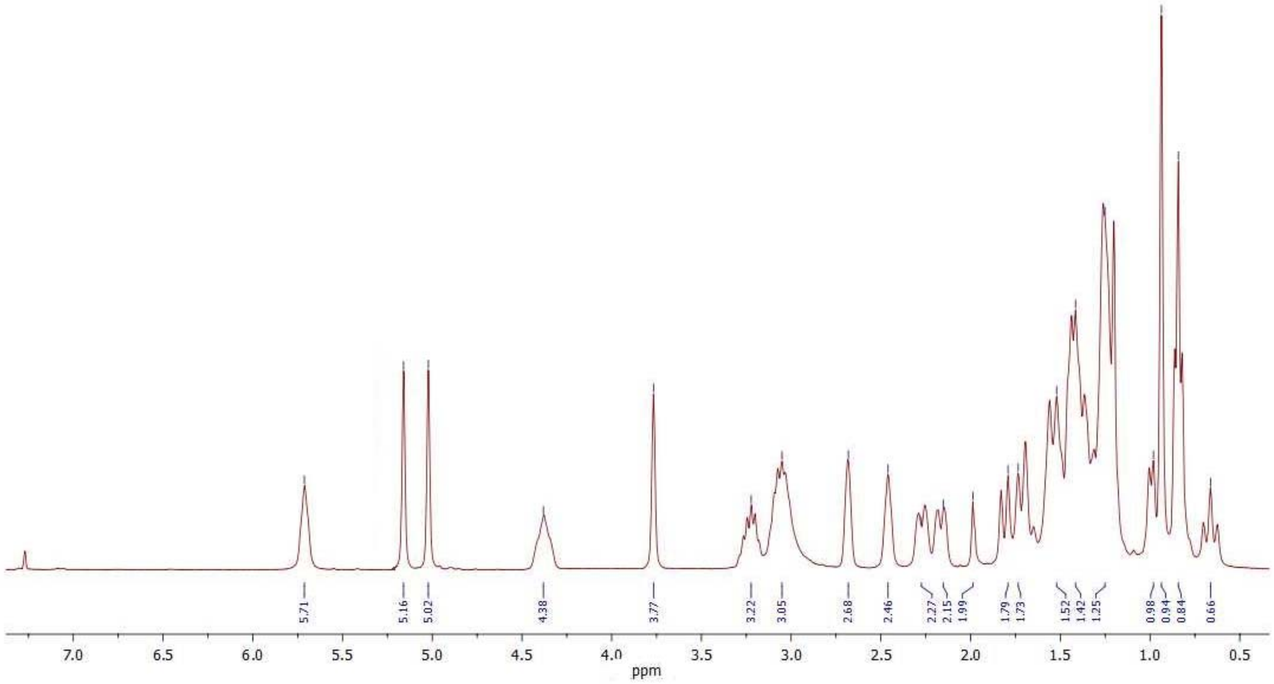

Figure S12. ^{1}H-NMR spectrum of compound **7**.

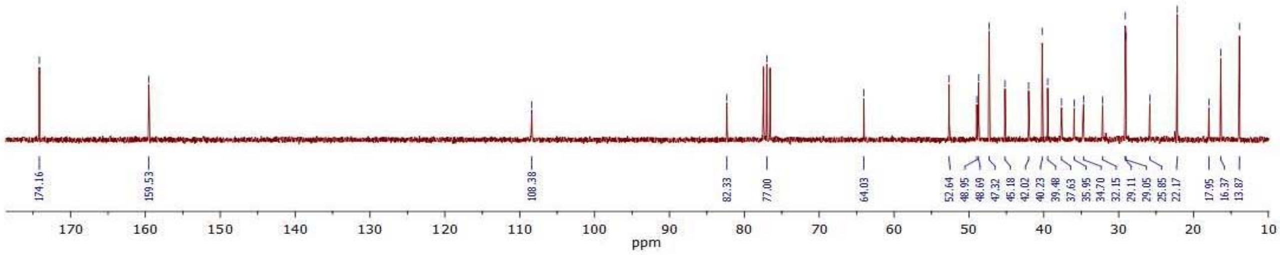

Figure S13. ^{13}C-NMR spectrum of compound **7**.

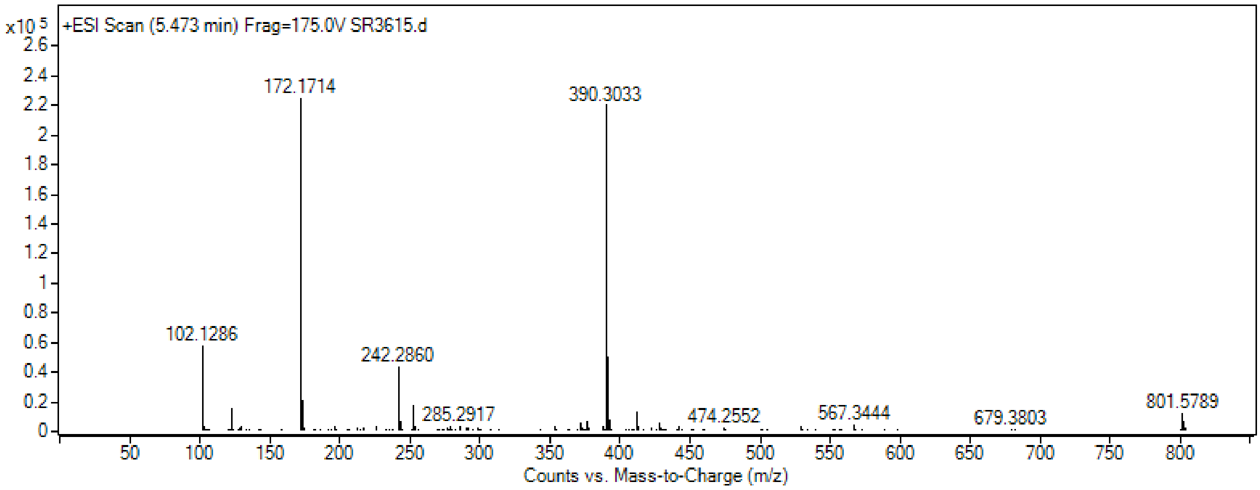

Figure S14. Mass spectrum of compound **7**.

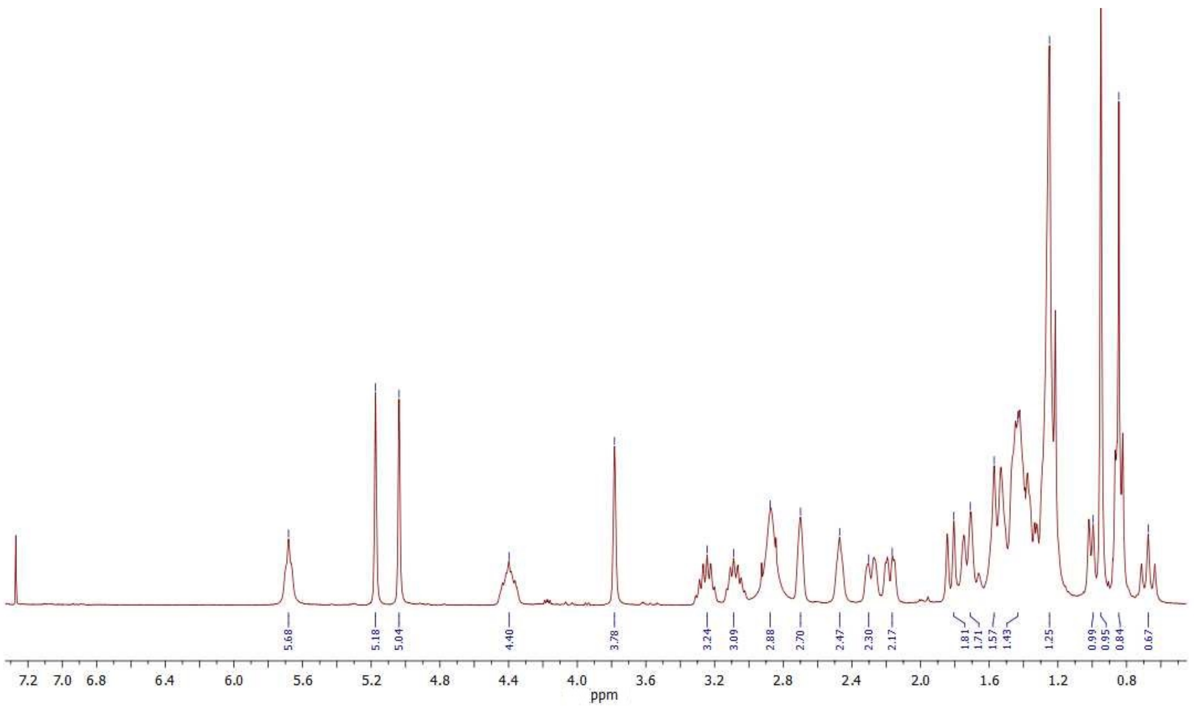

Figure S15. ^{1}H-NMR spectrum of compound **8**.

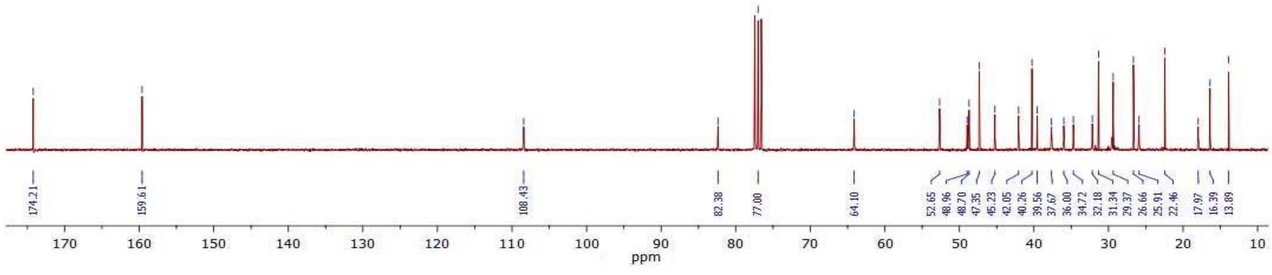

Figure S16. ^{13}C-NMR spectrum of compound **8**.

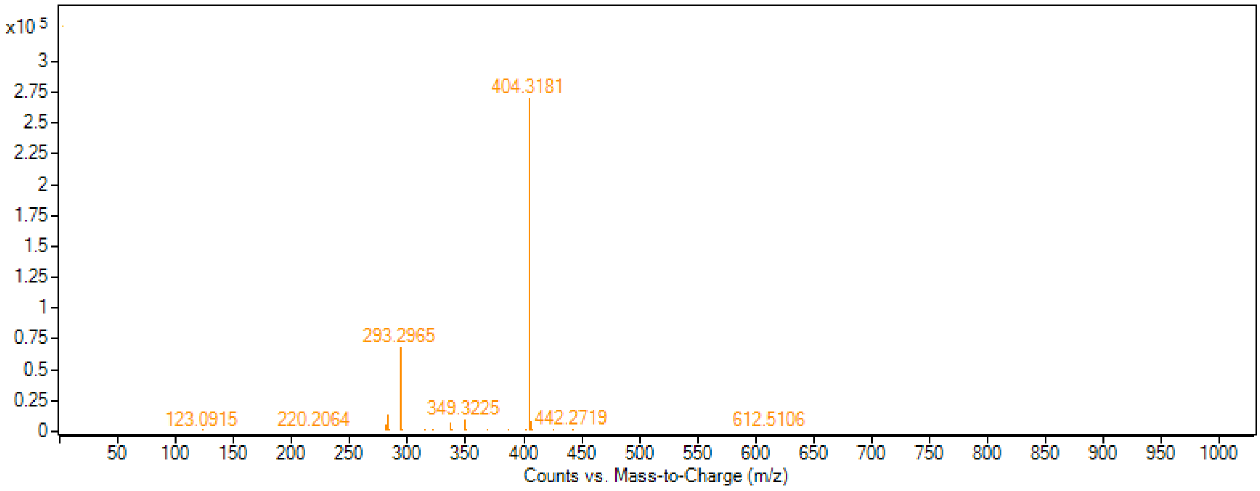

Figure S17. Mass spectrum of compound **8**.

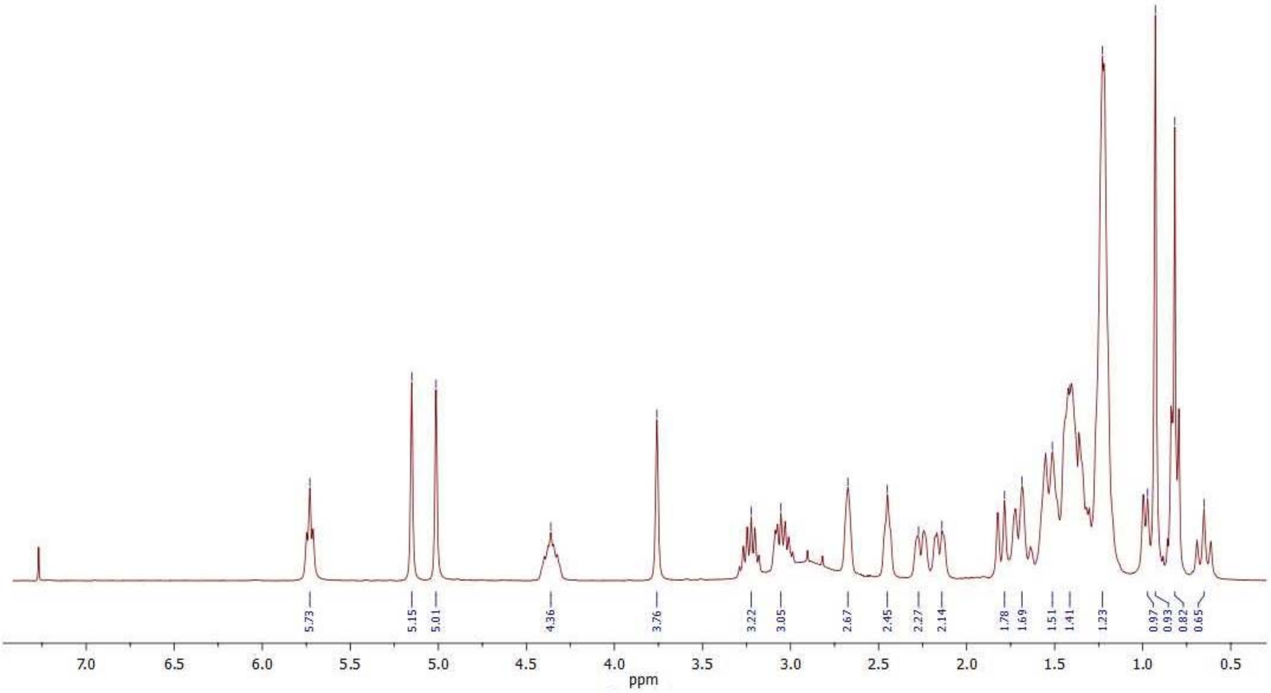

Figure S18. ^{1}H-NMR spectrum of compound **9**.

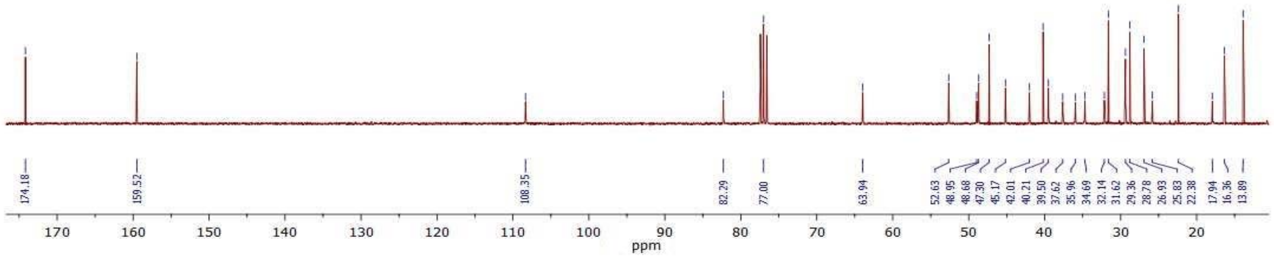

Figure S19. ^{13}C-NMR spectrum of compound **9**.

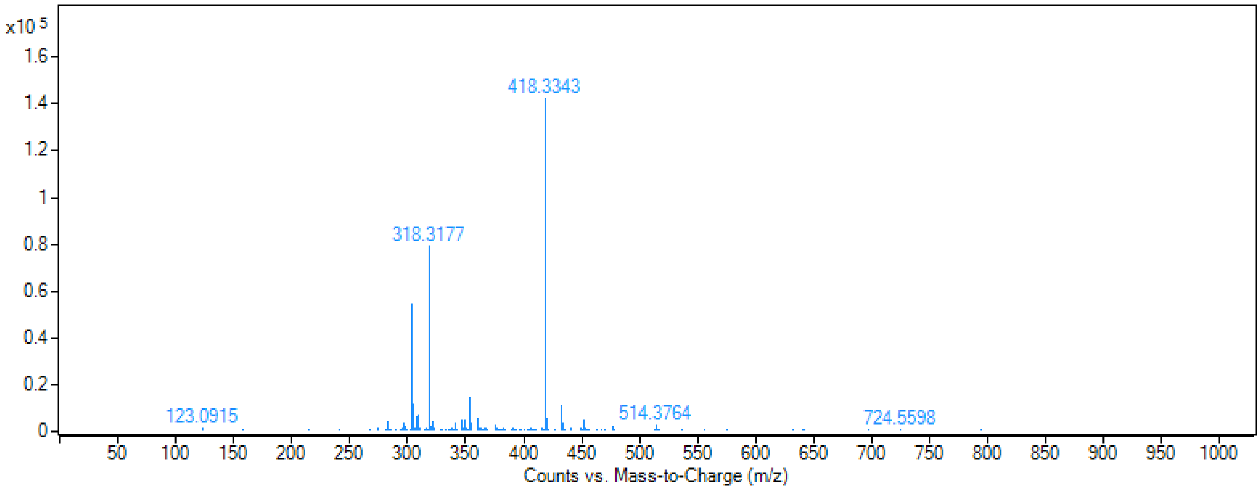

Figure S20. Mass spectrum of compound **9**.

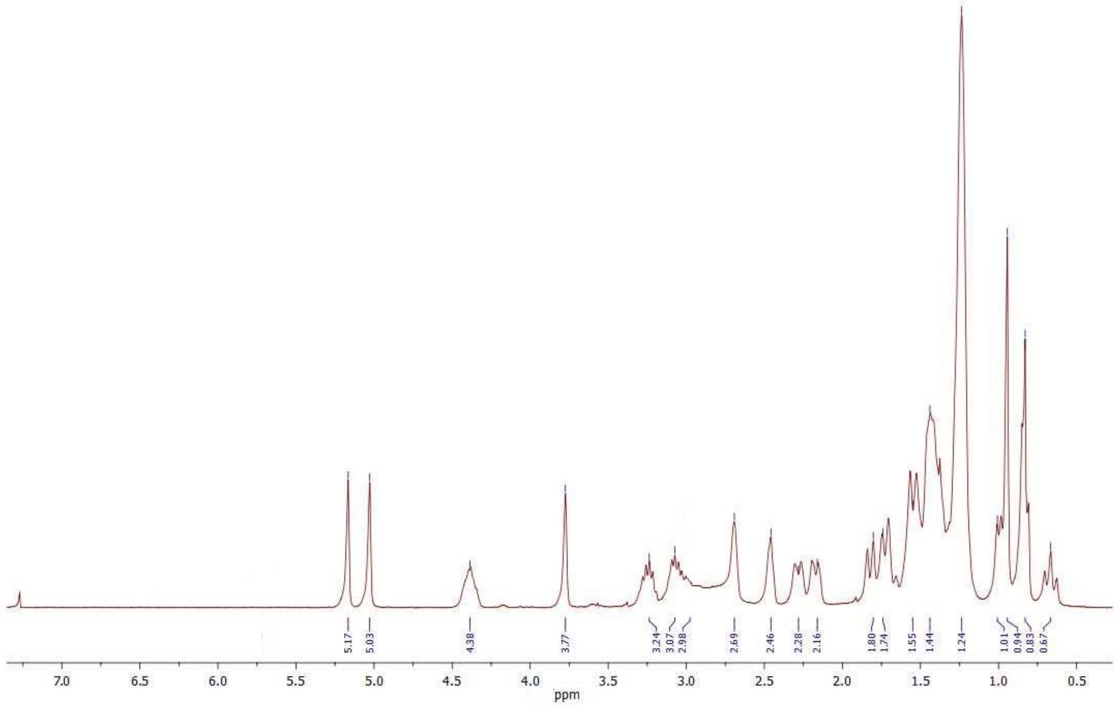

Figure S21. ¹H-NMR spectrum of compound **10**.

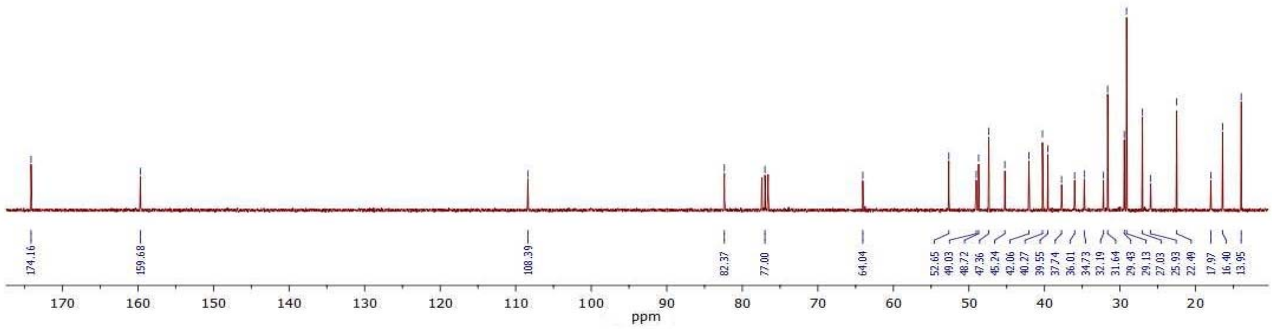

Figure S22. ¹³C-NMR spectrum of compound **10**.

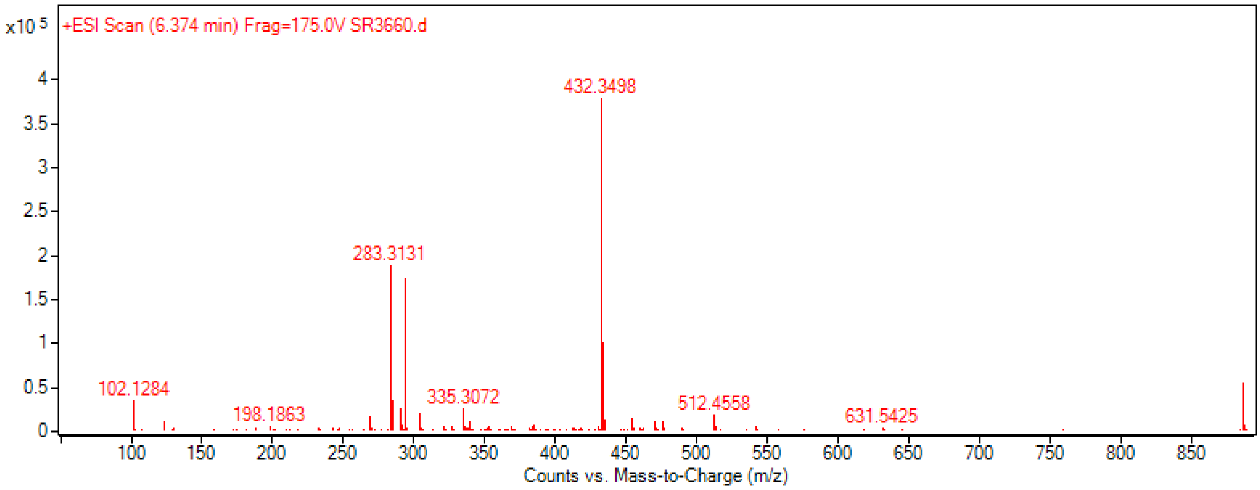

Figure S23. Mass spectrum of compound **10**.

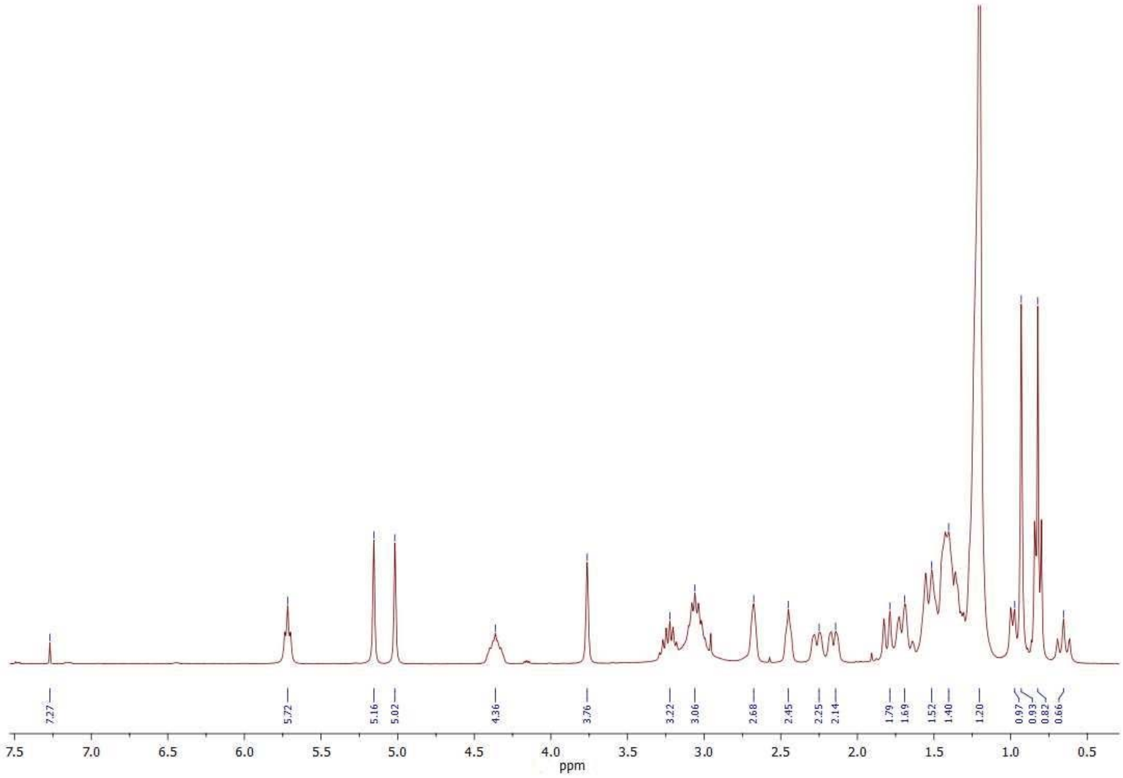

Figure S24. ^{1}H-NMR spectrum of compound **11**.

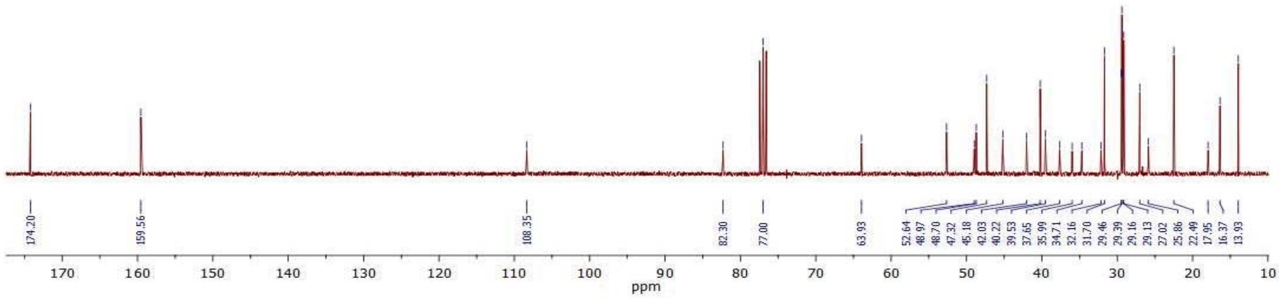

Figure S25. ^{13}C-NMR spectrum of compound **11**.

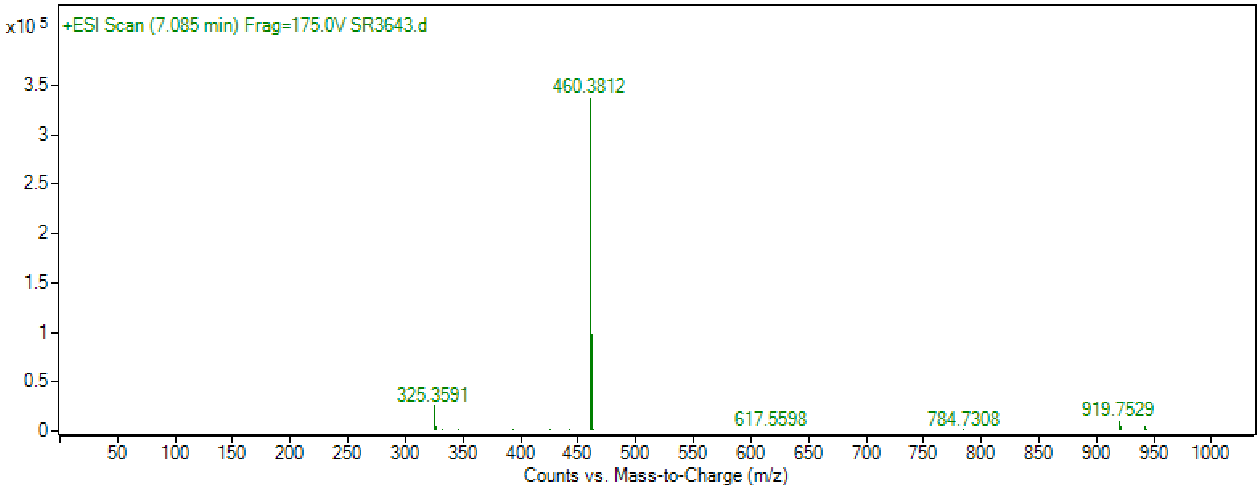

Figure S26. Mass spectrum of compound **11**.

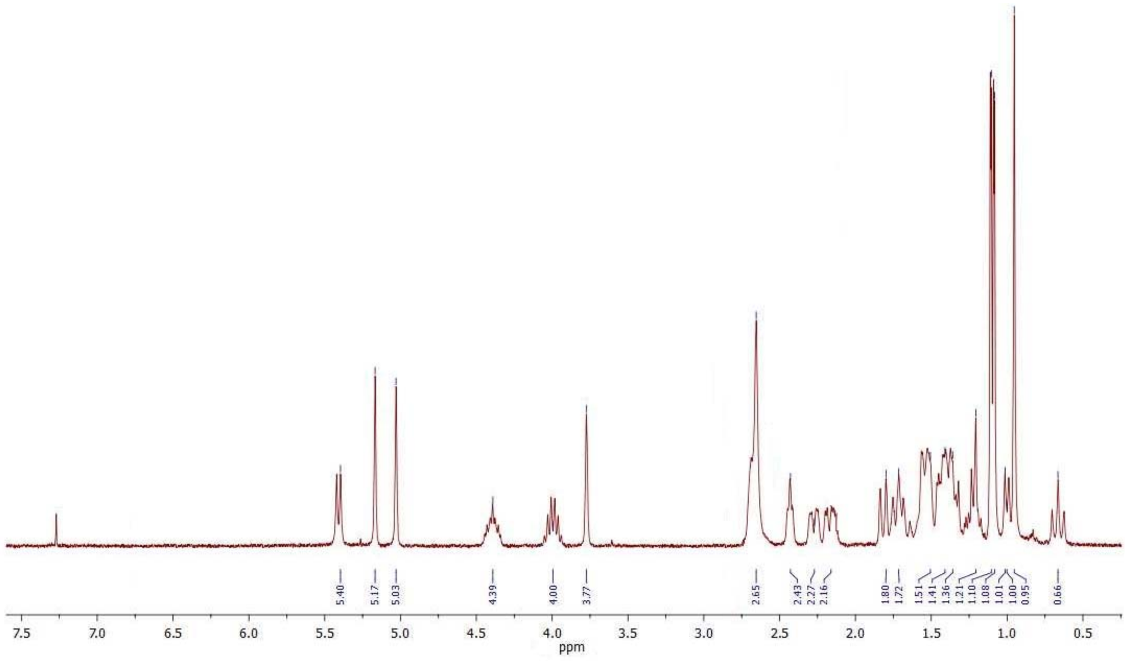

Figure S27. ¹H-NMR spectrum of compound **12**.

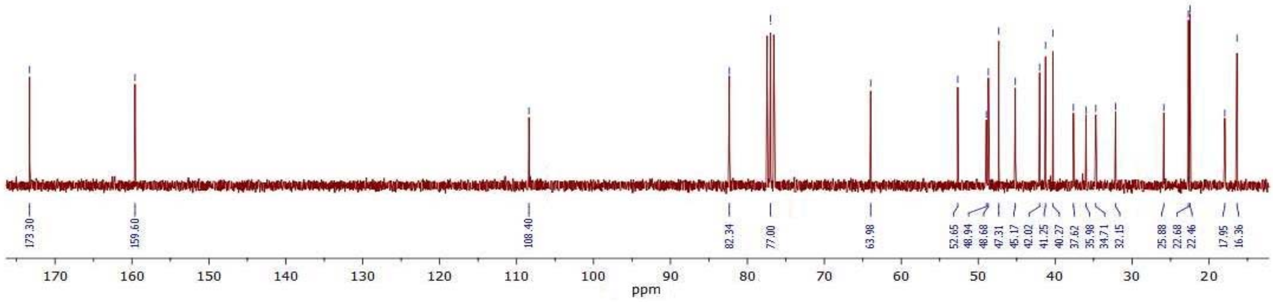

Figure S28. ¹³C-NMR spectrum of compound **12**.

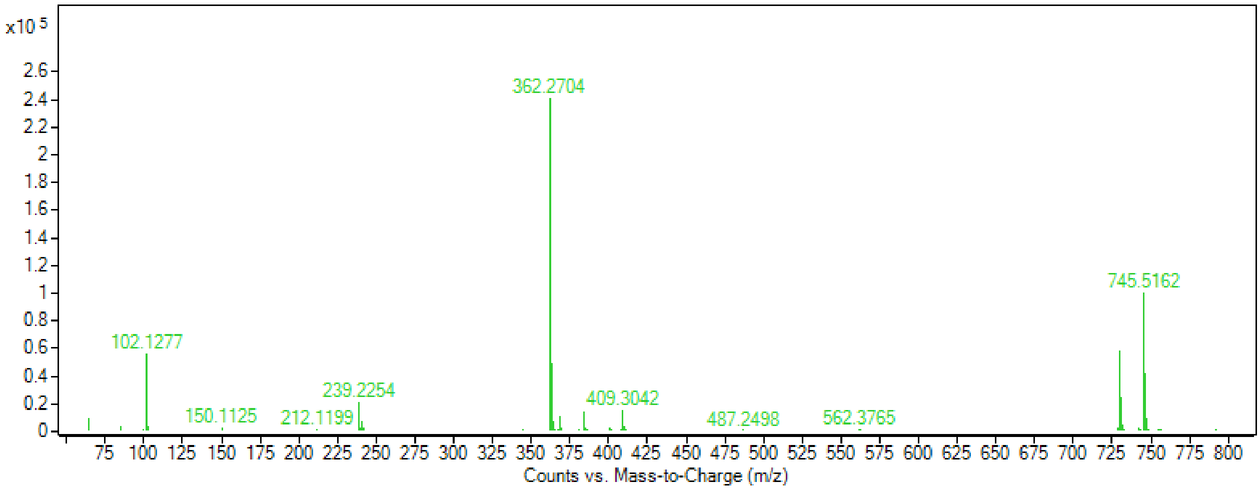

Figure S29. Mass spectrum of compound **12**.

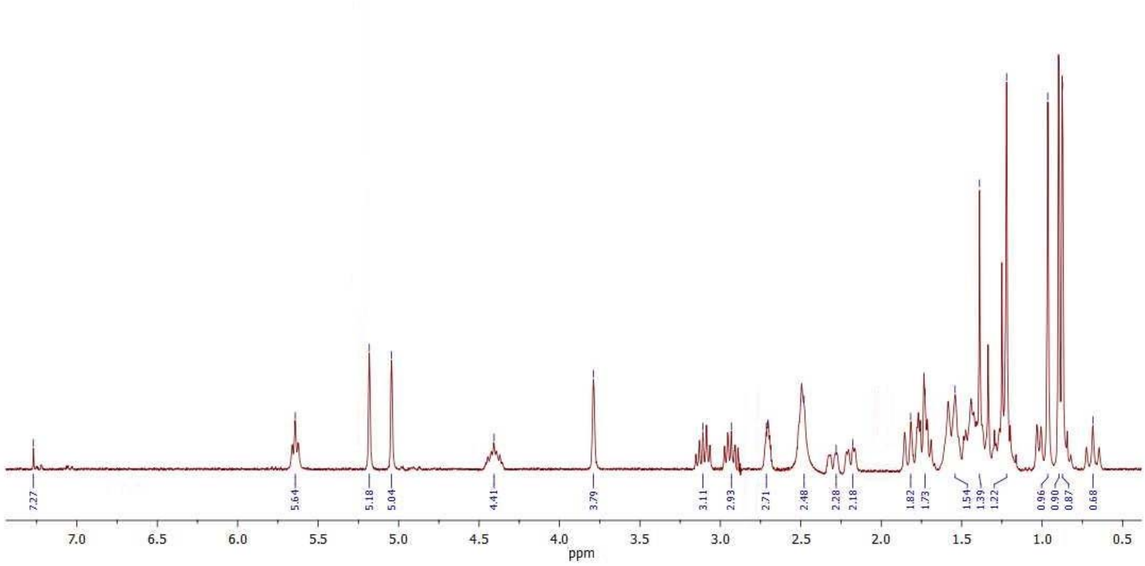

Figure S30. ^{1}H-NMR spectrum of compound **13**.

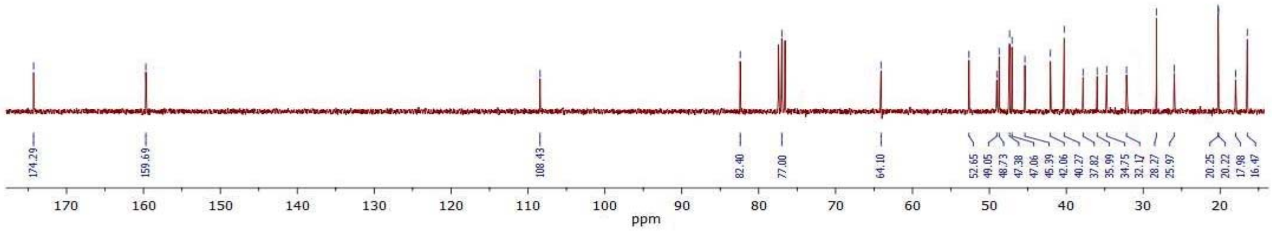

Figure S31. ^{13}C-NMR spectrum of compound **13**.

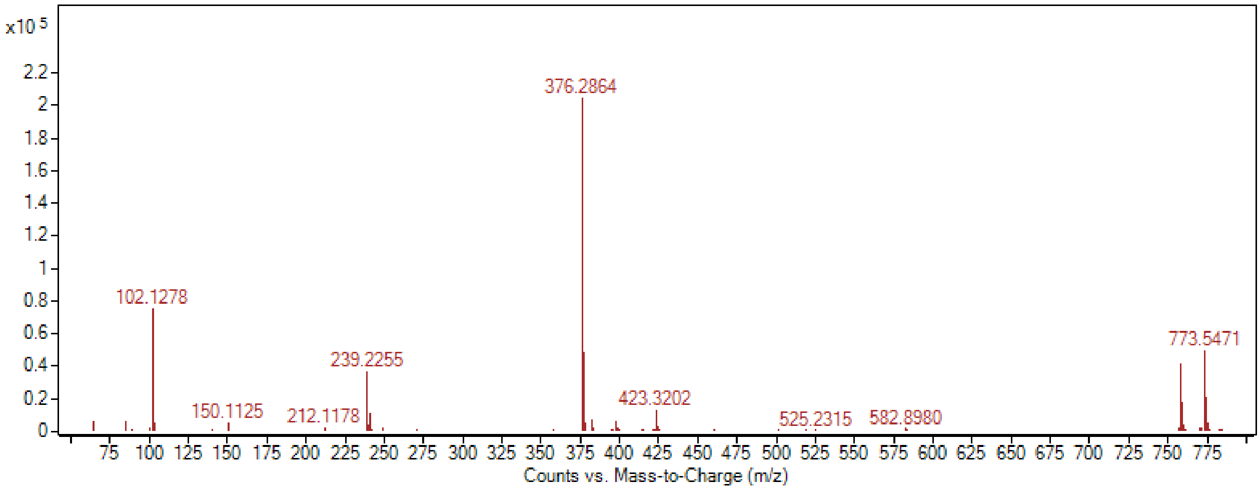

Figure S32. Mass spectrum of compound **13**.

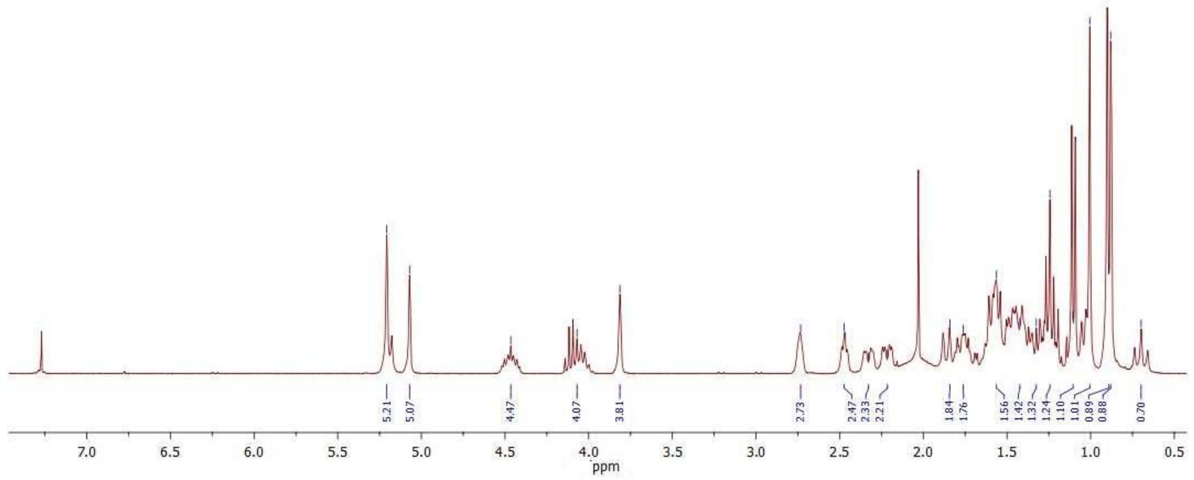

Figure S33. ^{1}H-NMR spectrum of compound **14**.

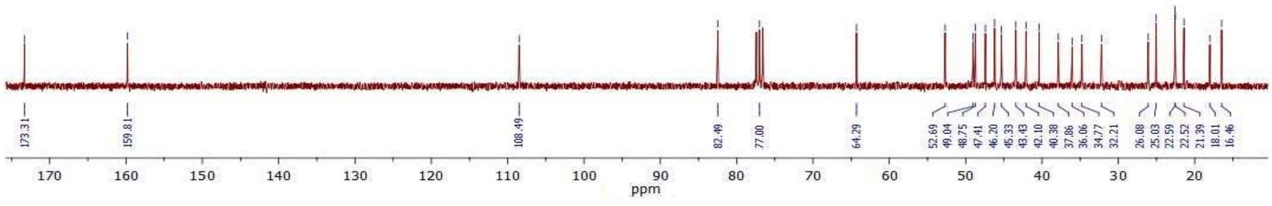

Figure S34. ^{13}C-NMR spectrum of compound **14**.

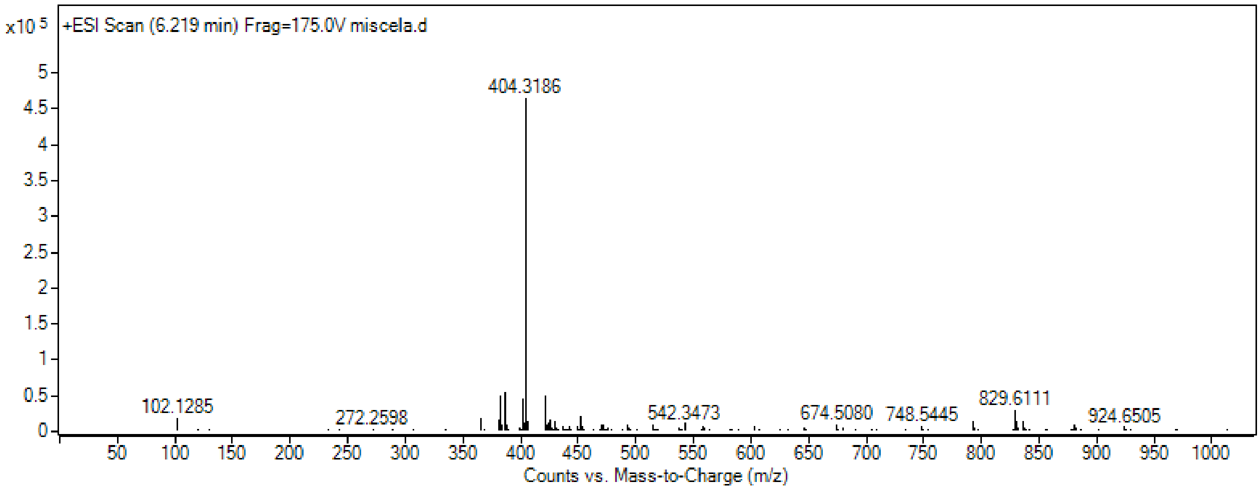

Figure S35. Mass spectrum of compound **14**.

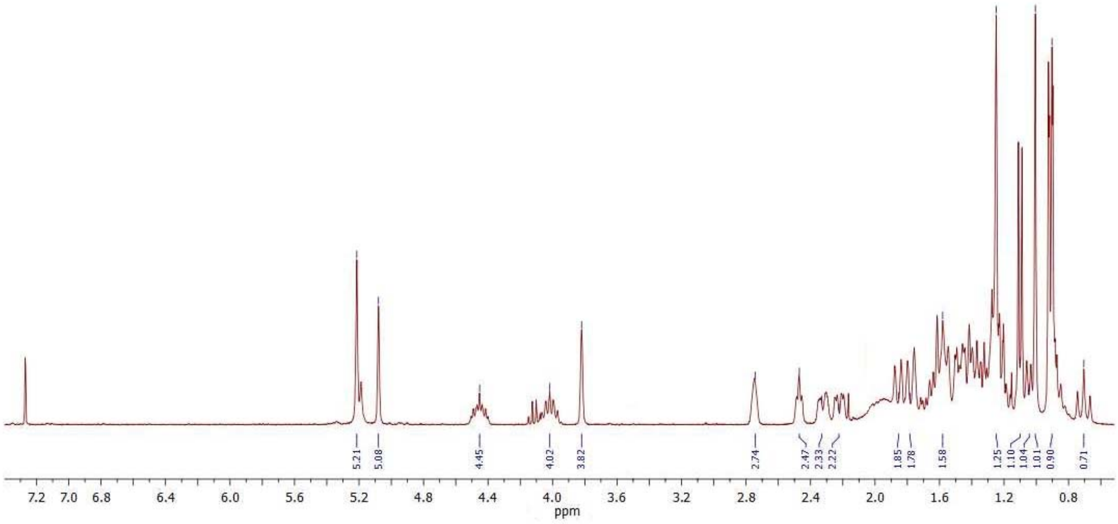

Figure S36. ^{1}H-NMR spectrum of compound **15**.

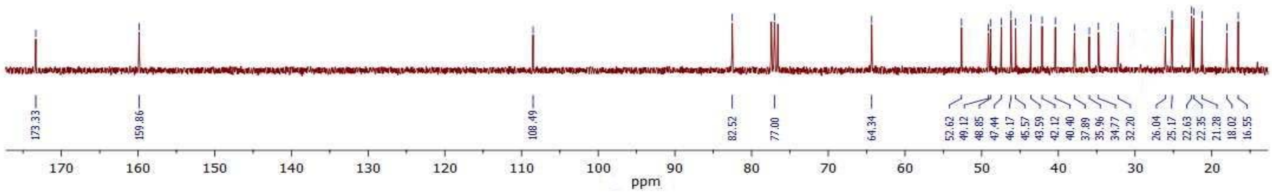

Figure S37. ^{13}C-NMR spectrum of compound **15**.

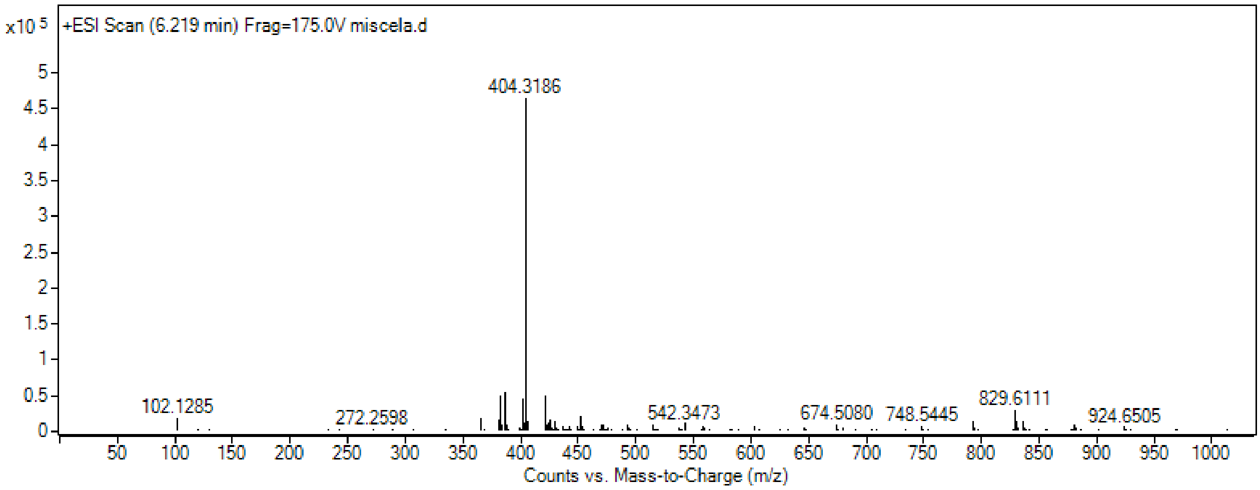

Figure S38. Mass spectrum of compound **15**.

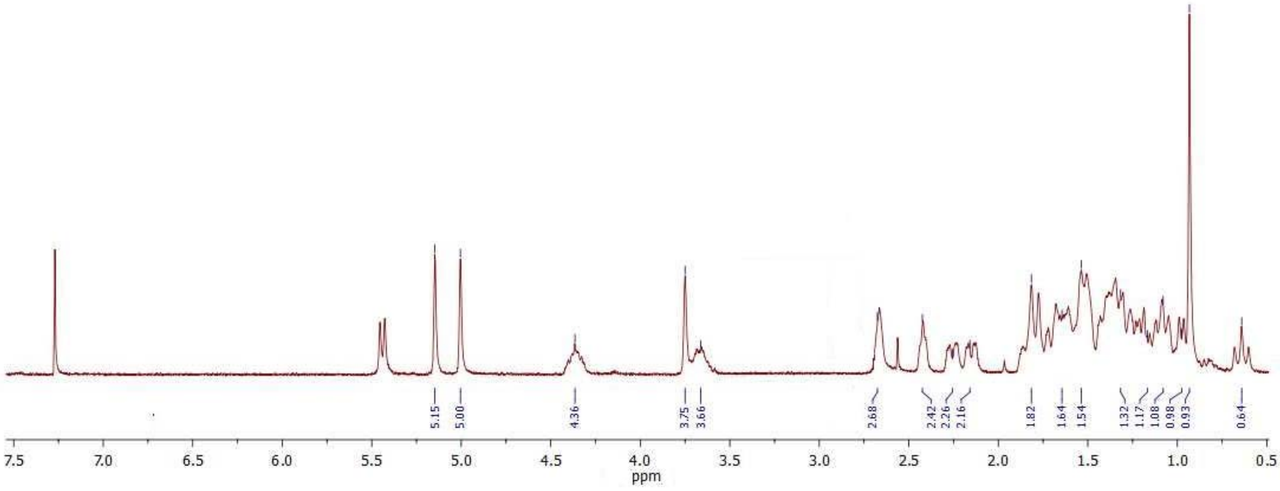

Figure S39. ^{1}H-NMR spectrum of compound **16**.

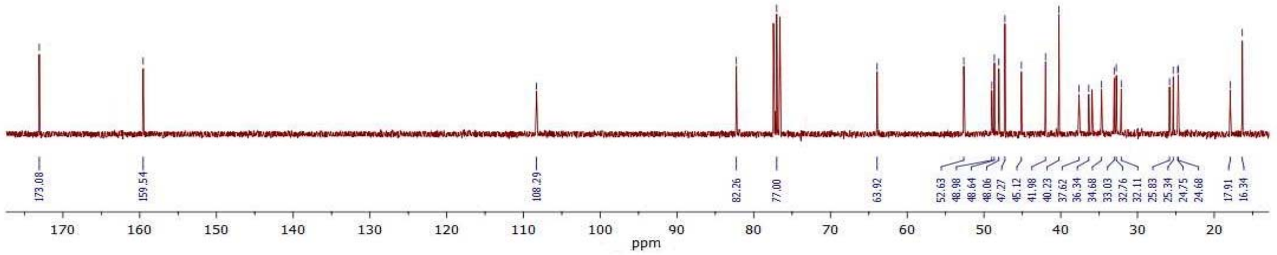

Figure S40. ^{13}C-NMR spectrum of compound **16**.

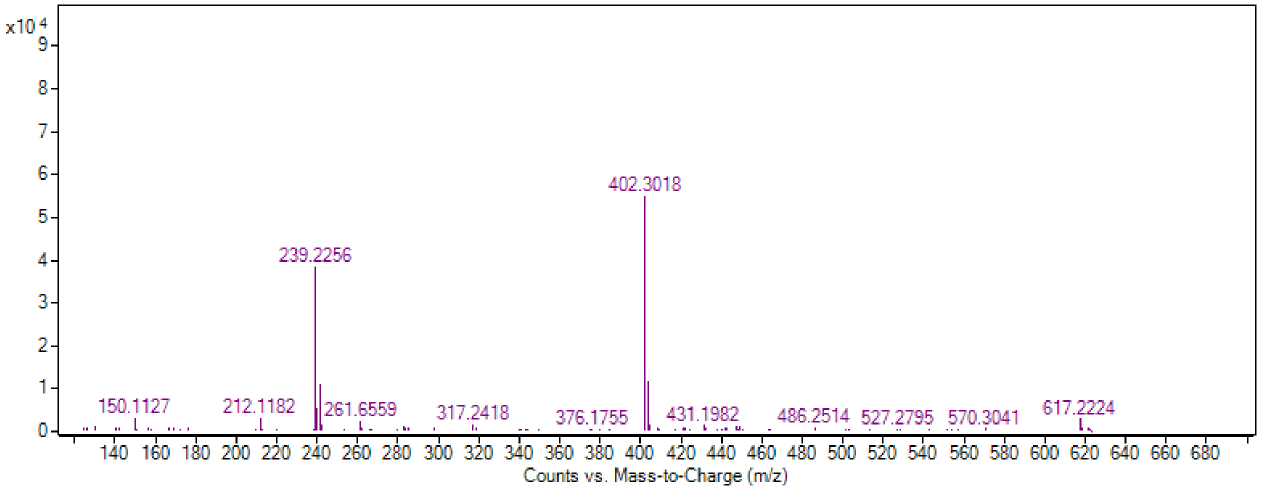

Figure S41. Mass spectrum of compound **16**.

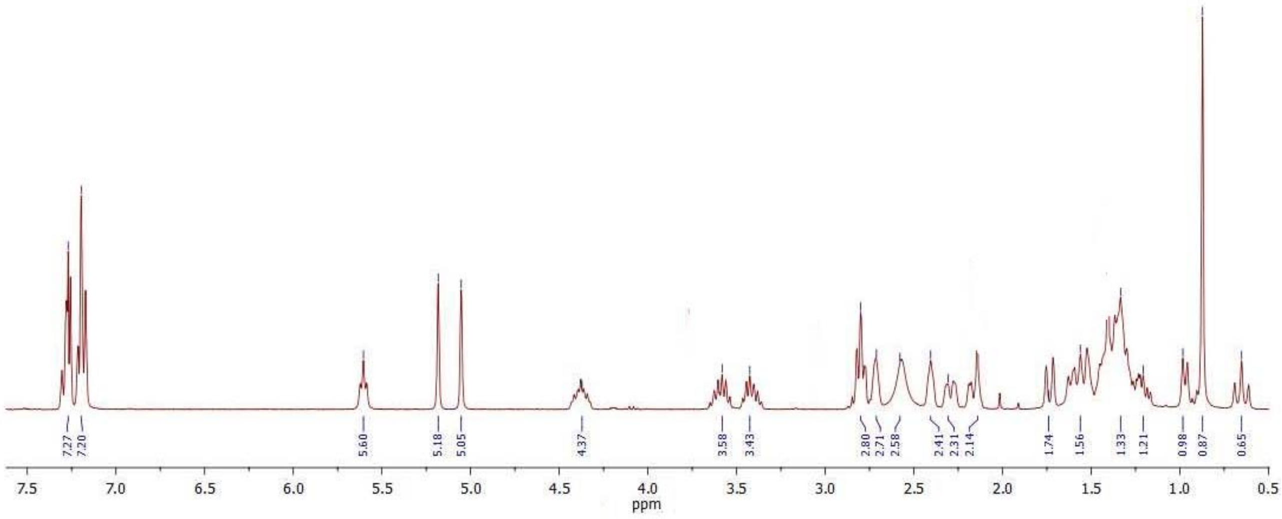

Figure S42. ^{1}H-NMR spectrum of compound **17**.

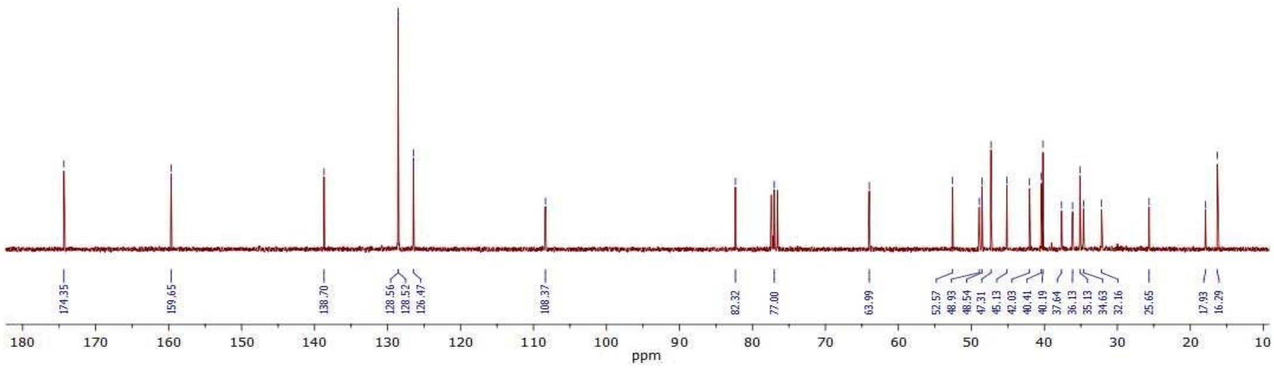

Figure S43. ^{13}C-NMR spectrum of compound **17**.

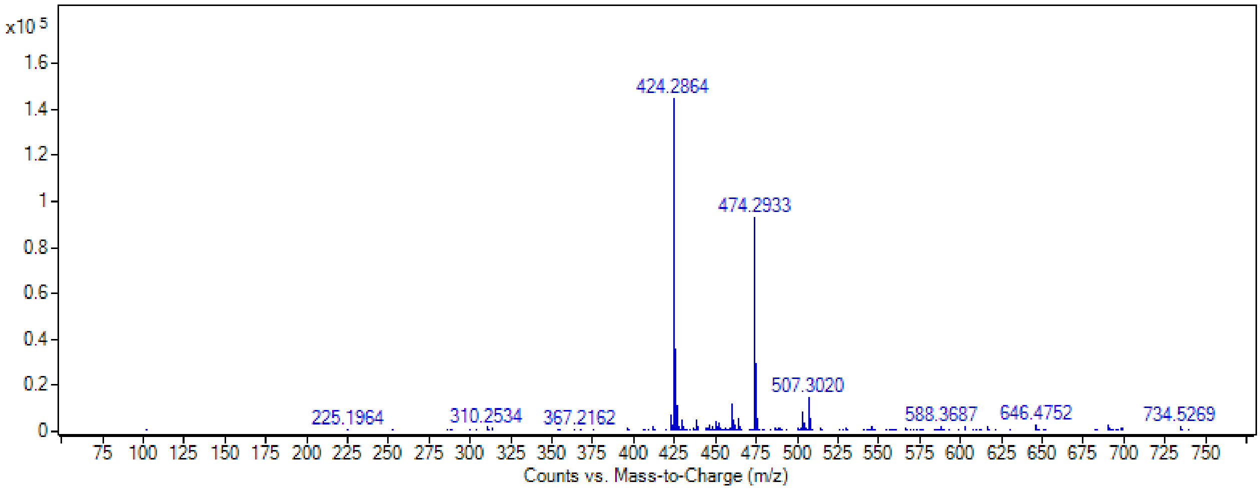

Figure S44. Mass spectrum of compound **17**.

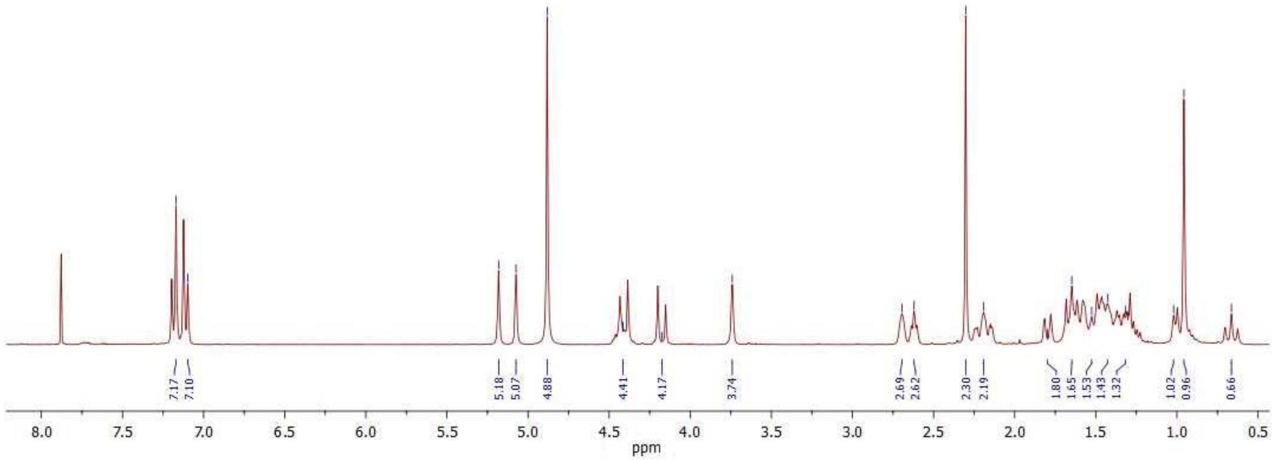

Figure S45. ^{1}H-NMR spectrum of compound **18**.

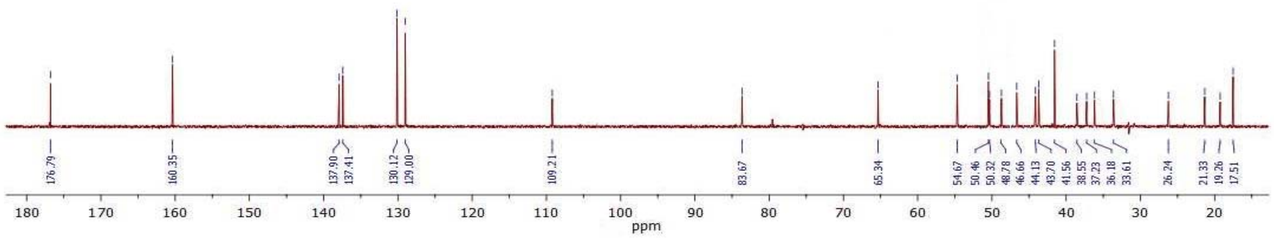

Figure S46. ^{13}C-NMR spectrum of compound **18**.

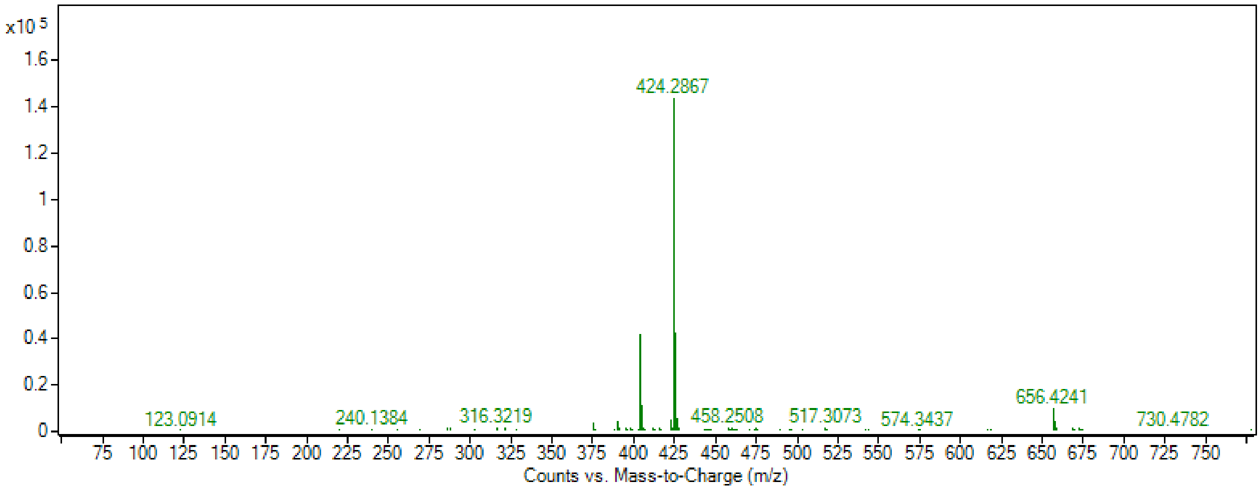

Figure S47. Mass spectrum of compound **18**.

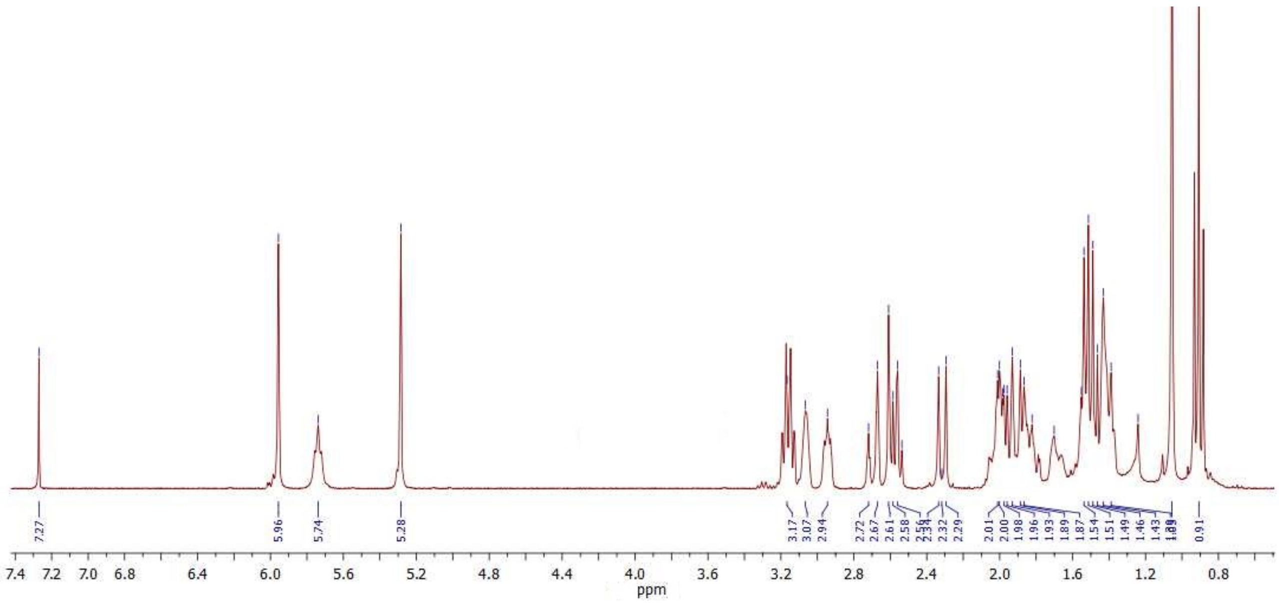

Figure S48. ^{1}H-NMR spectrum of compound **19**.

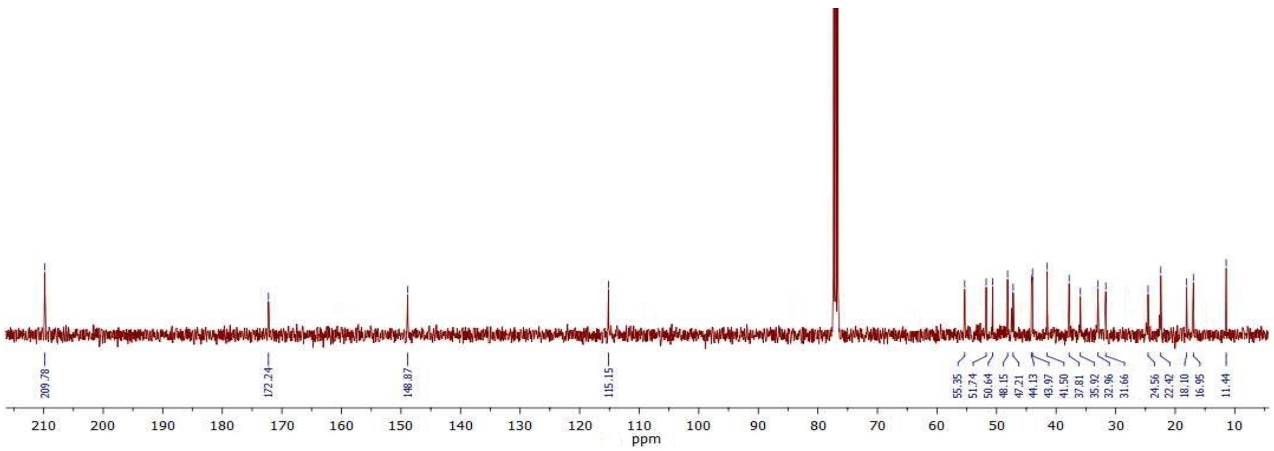

Figure S49. ^{13}C-NMR spectrum of compound **19**.

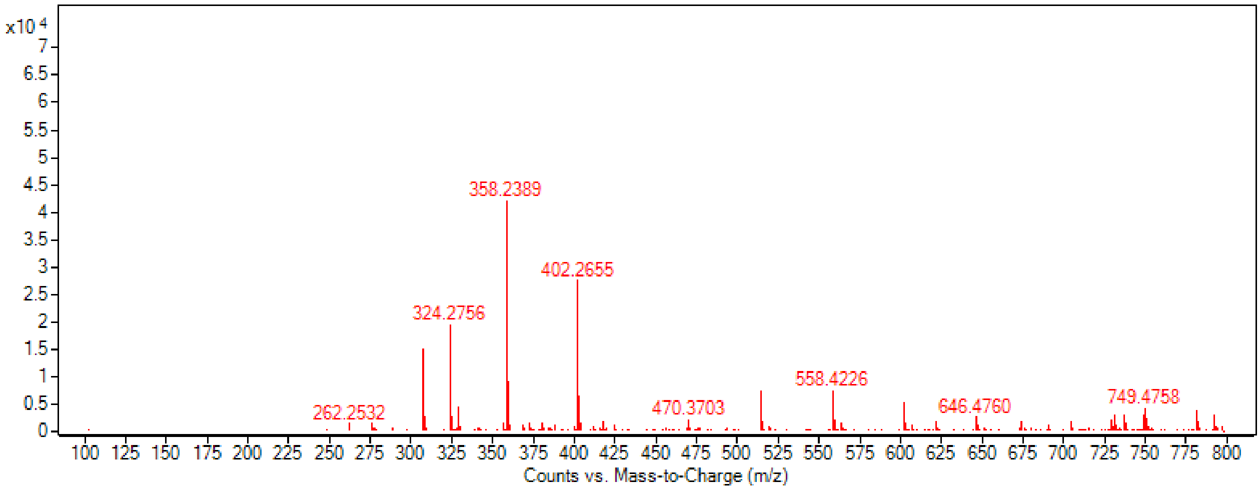

Figure S50. Mass spectrum of compound **19**.

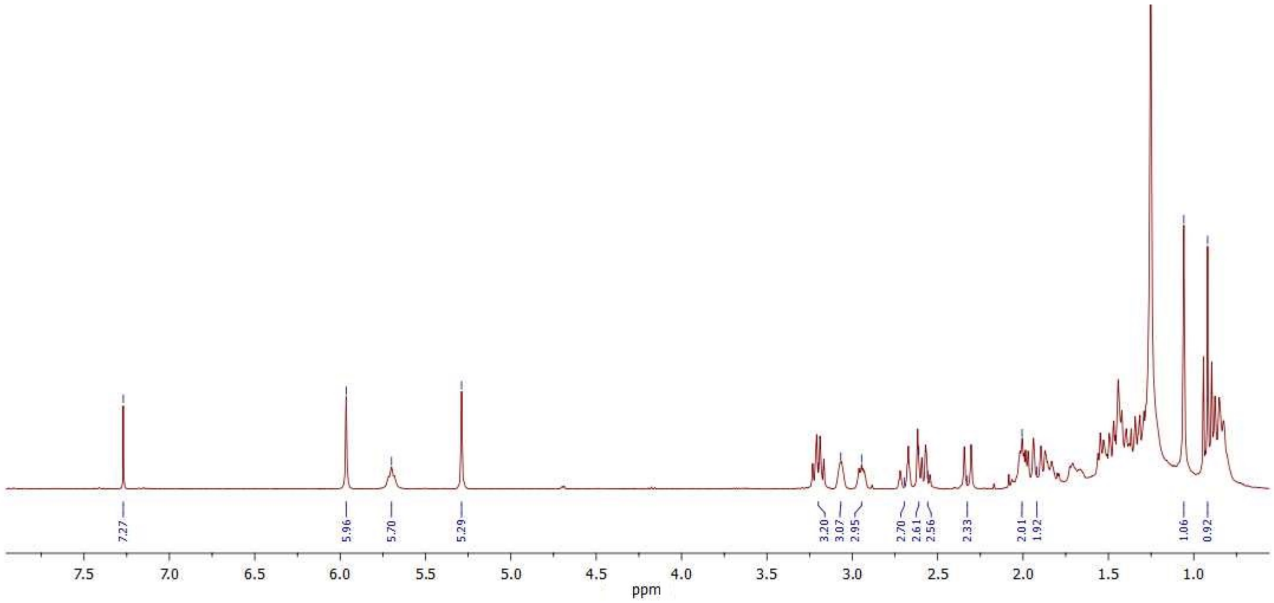

Figure S51. ^{1}H-NMR spectrum of compound **20**.

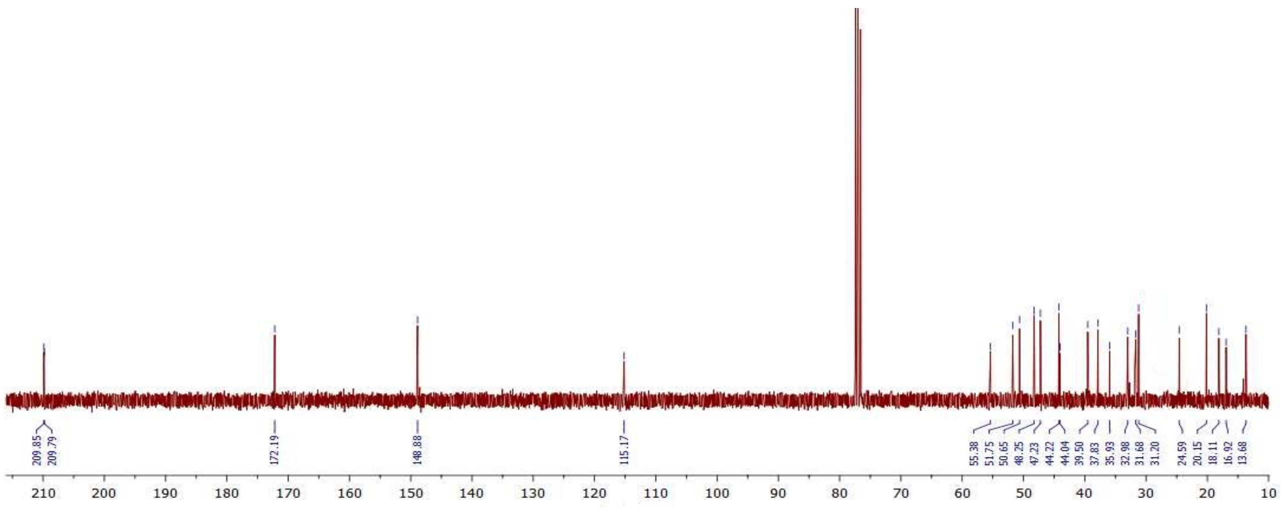

Figure S52. ^{13}C-NMR spectrum of compound **20**.

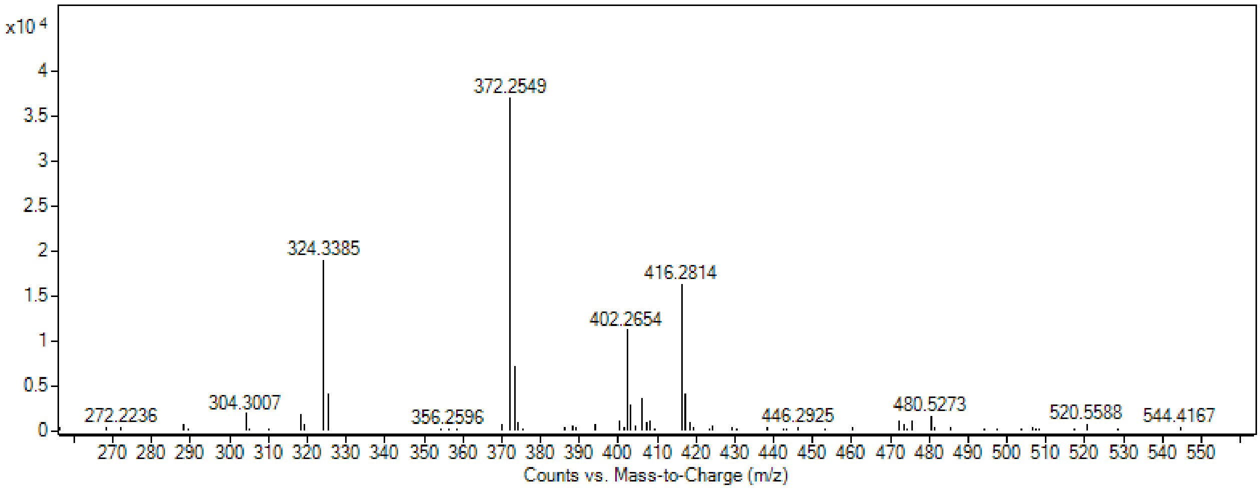

Figure S53. Mass spectrum of compound **20**.

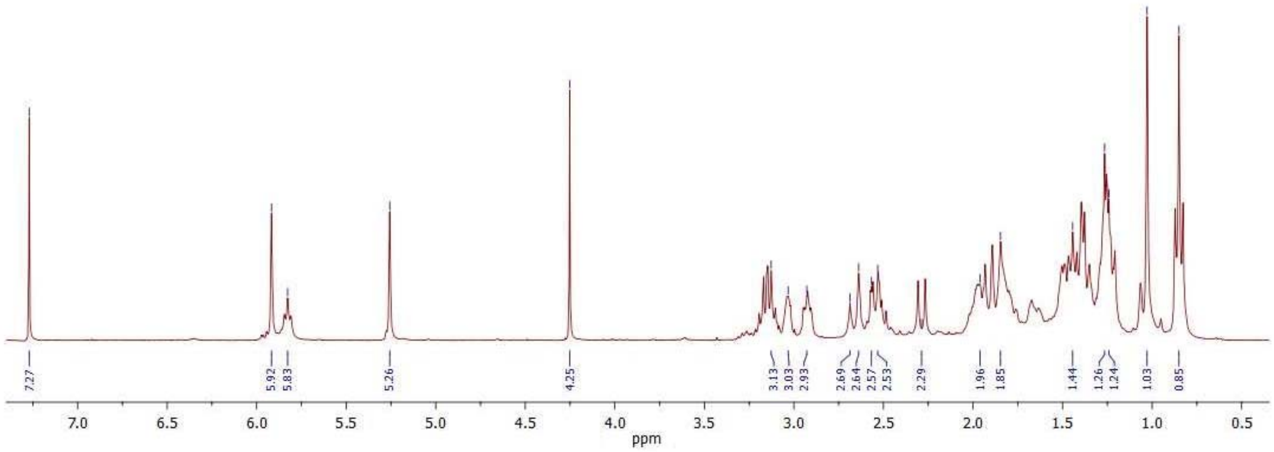

Figure S54. ^{1}H-NMR spectrum of compound **21**.

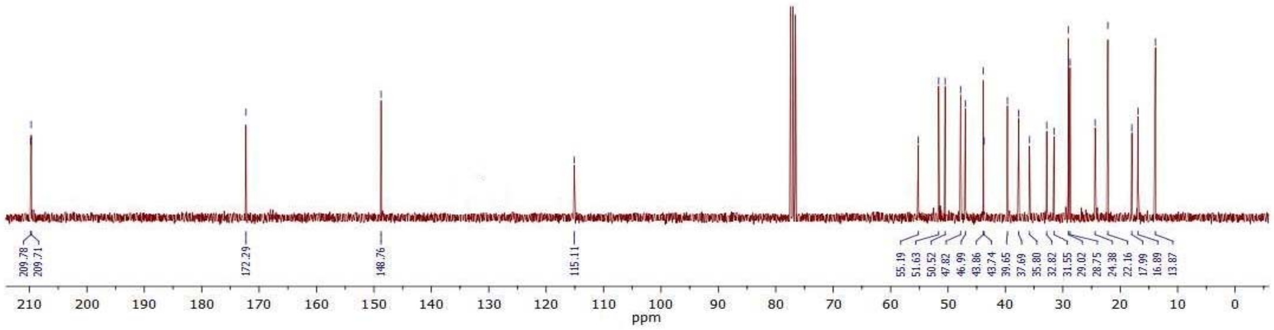

Figure S55. ^{13}C-NMR spectrum of compound **21**.

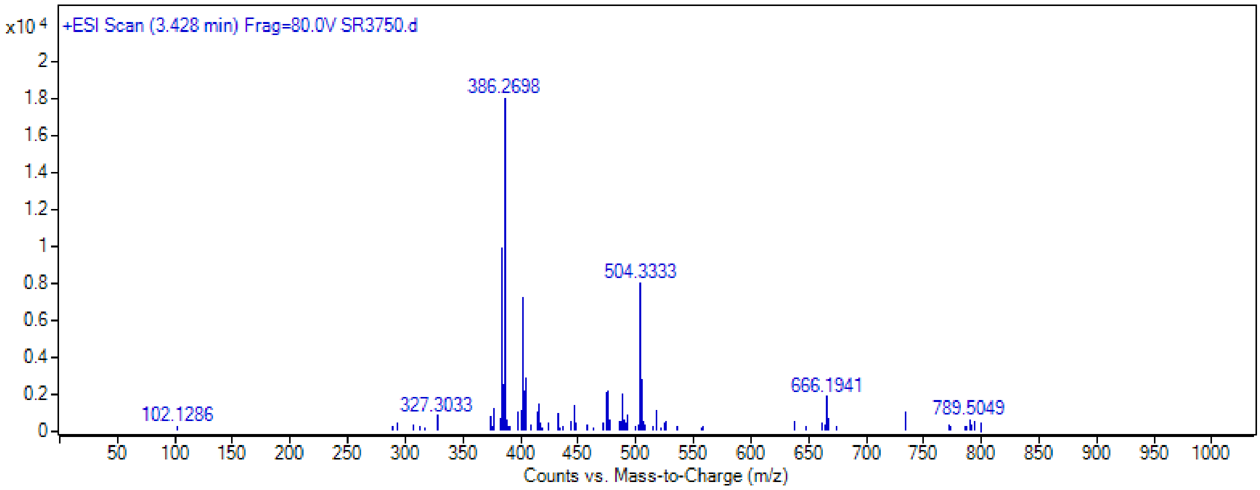

Figure S56. Mass spectrum of compound **21**.

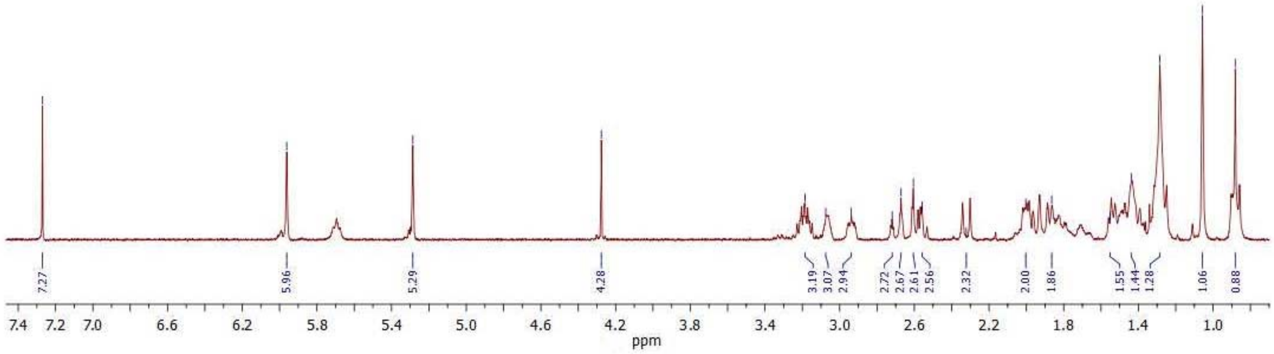

Figure S57. ^{1}H-NMR spectrum of compound **22**.

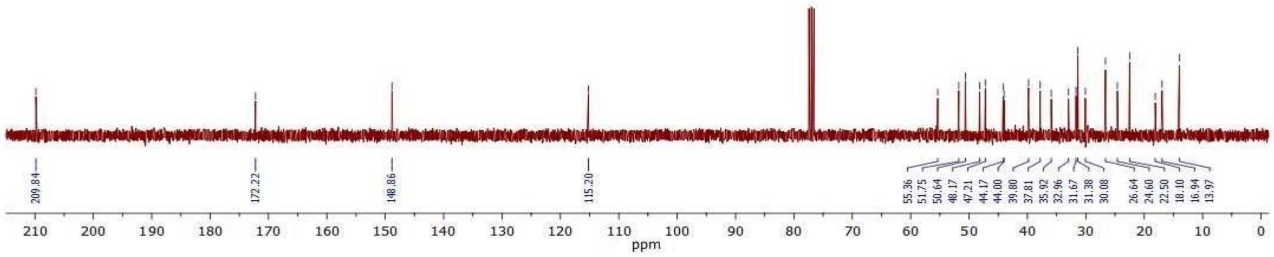

Figure S58. ^{13}C-NMR spectrum of compound **22**.

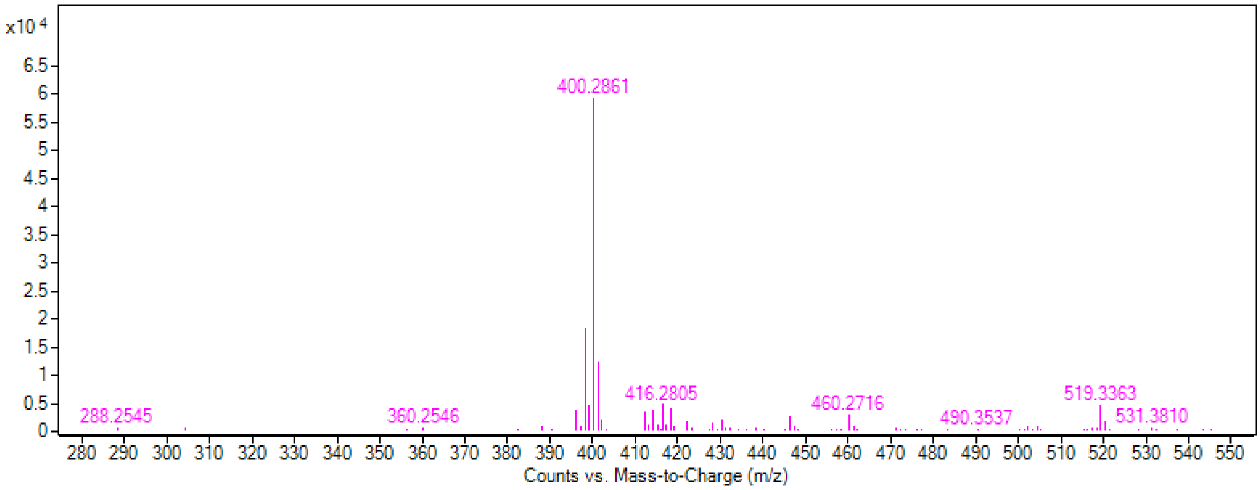

Figure S59. Mass spectrum of compound **22**.

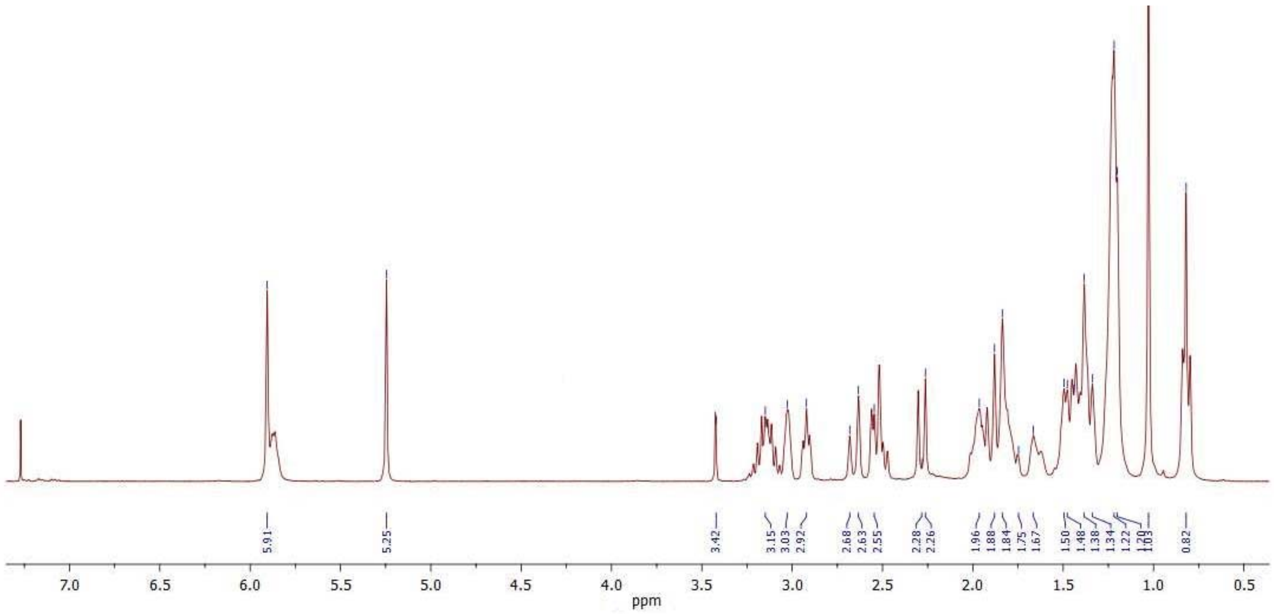

Figure S60. ^{1}H-NMR spectrum of compound **23**.

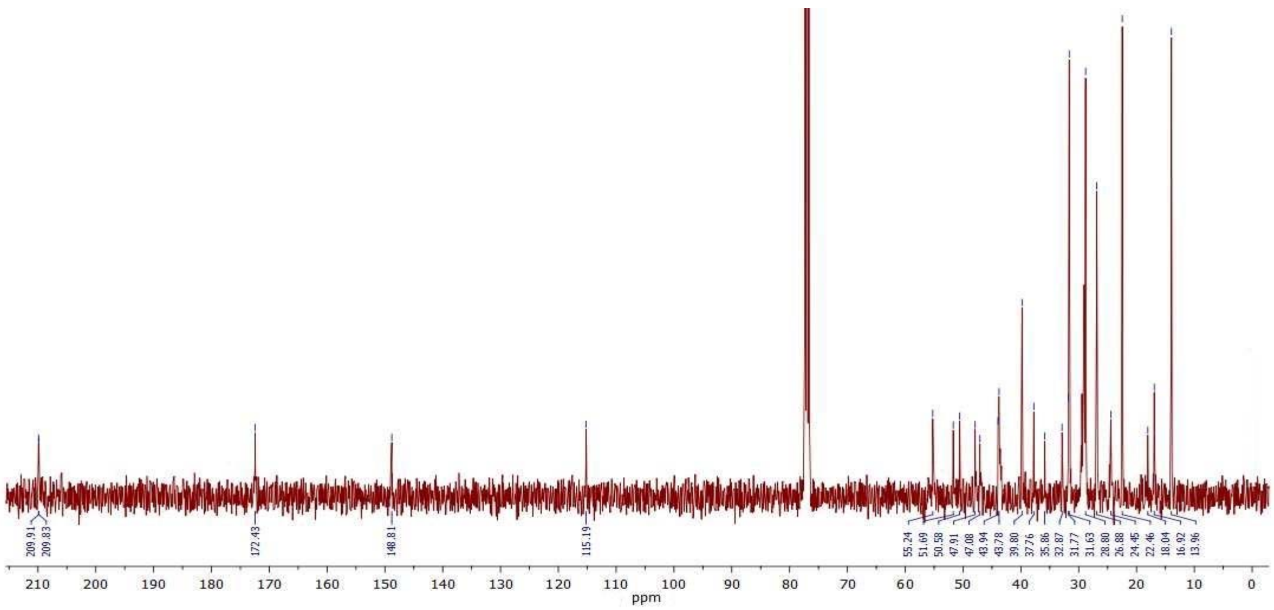

Figure S61. ^{13}C-NMR spectrum of compound **23**.

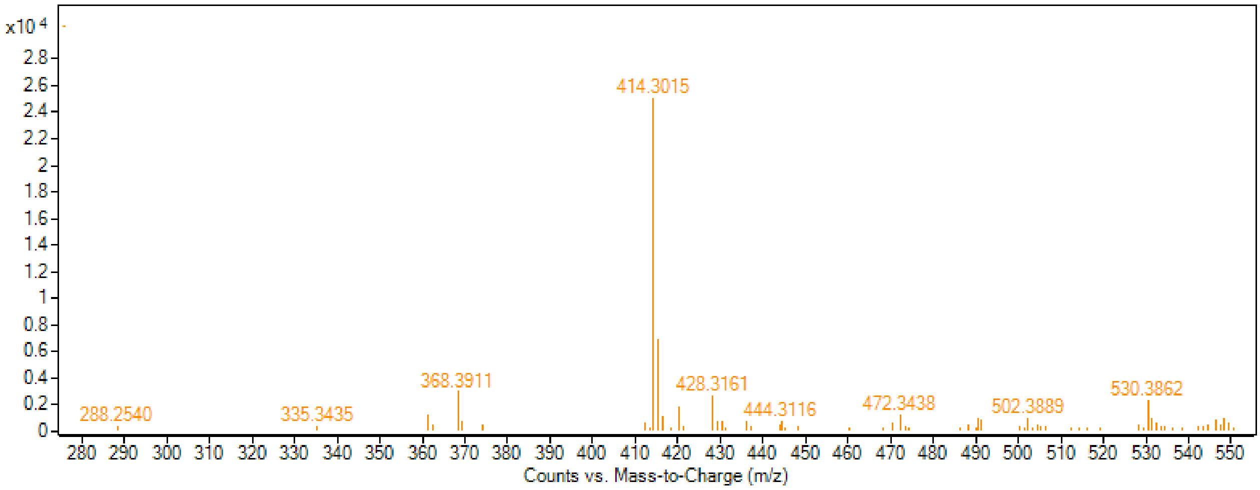

Figure S62. Mass spectrum of compound **23**.

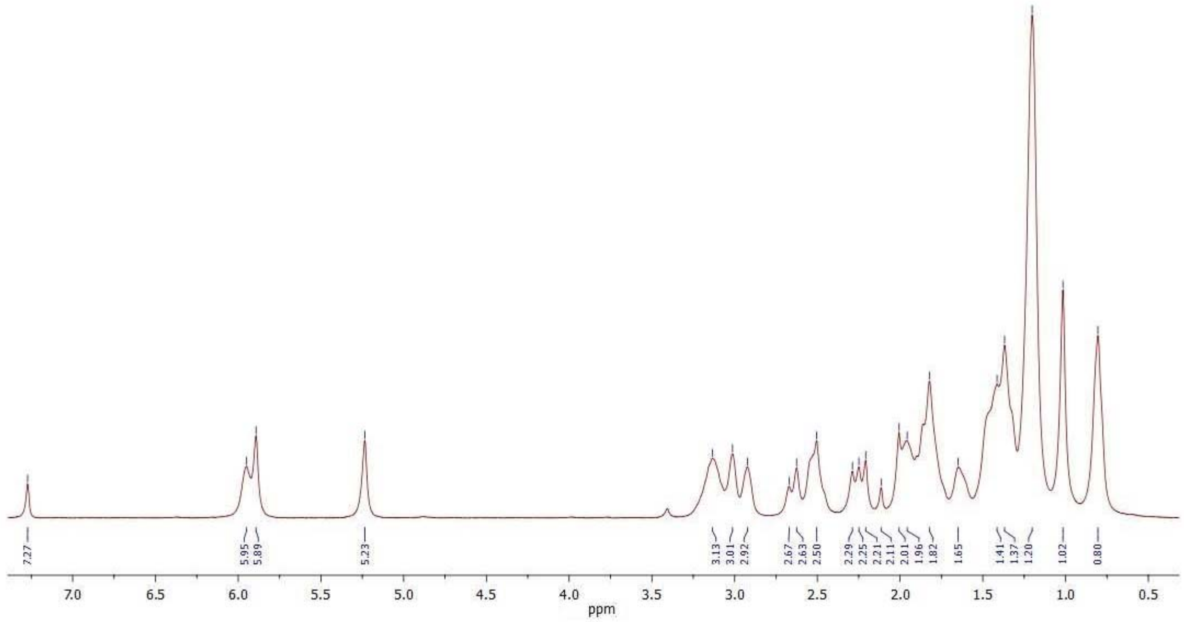

Figure S63. ^{1}H-NMR spectrum of compound **24**.

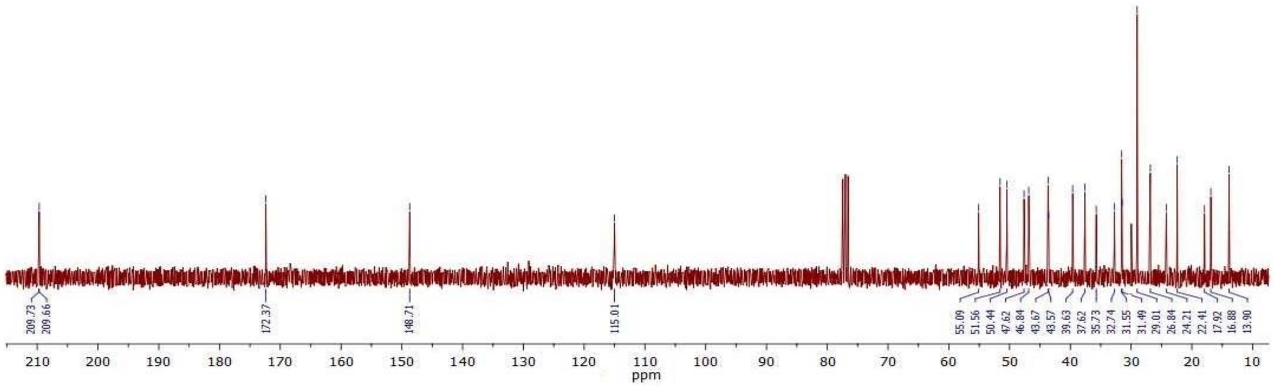

Figure S64. ^{13}C-NMR spectrum of compound **24**.

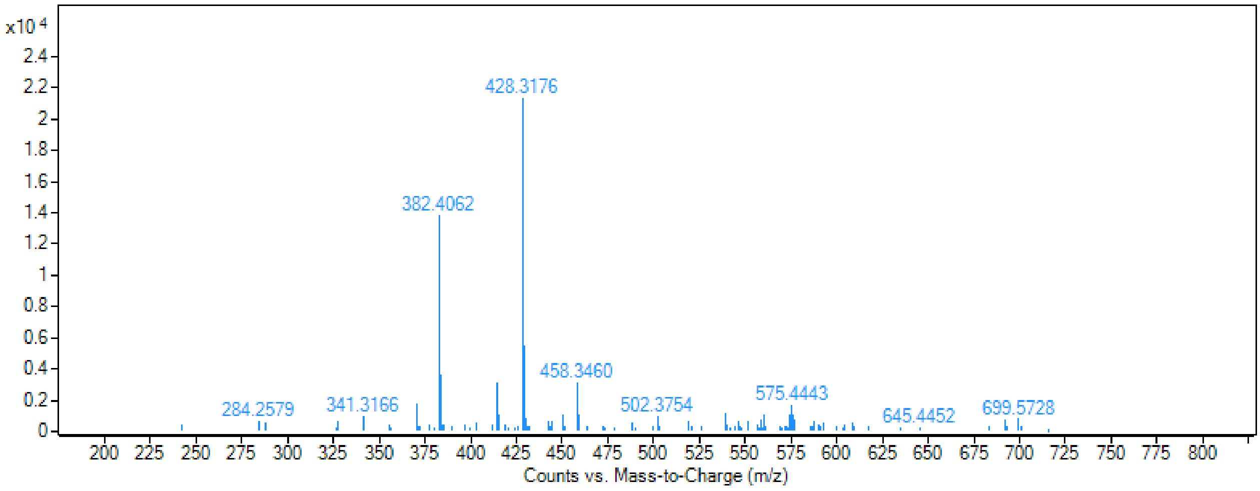

Figure S65. Mass spectrum of compound **24**.

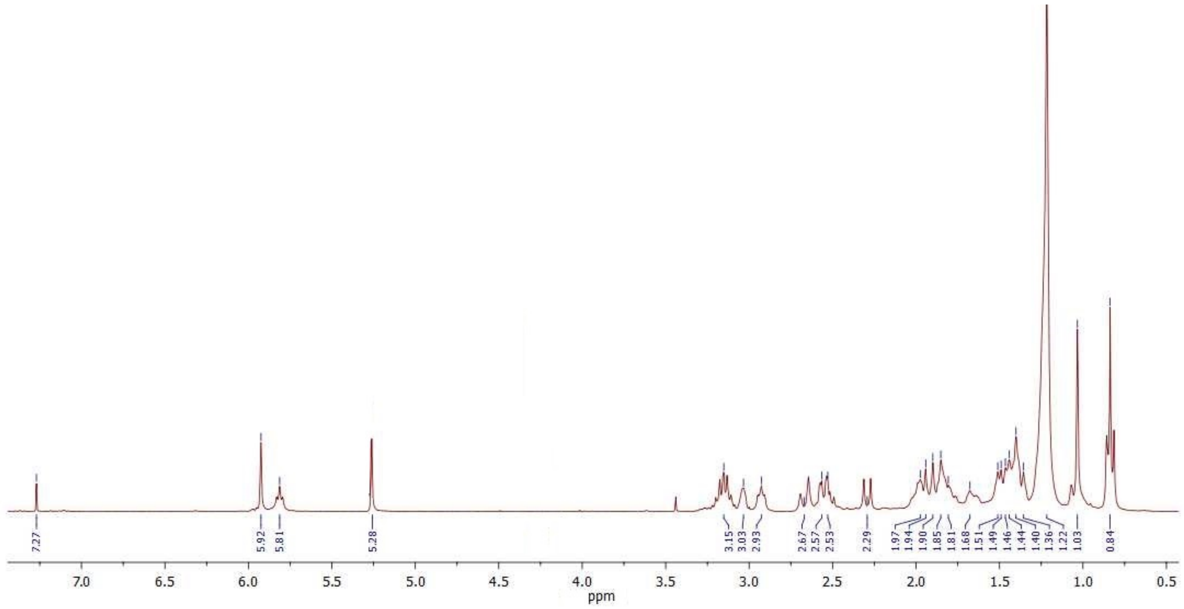

Figure S66. ^{1}H-NMR spectrum of compound **25**.

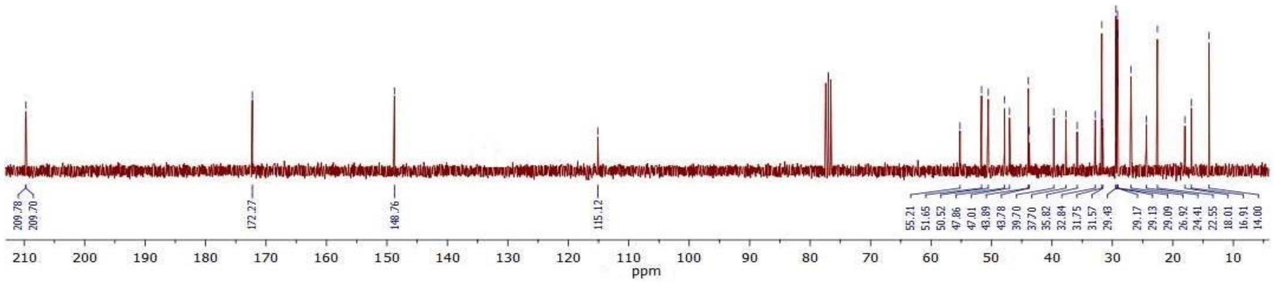

Figure S67. ^{13}C-NMR spectrum of compound **25**.

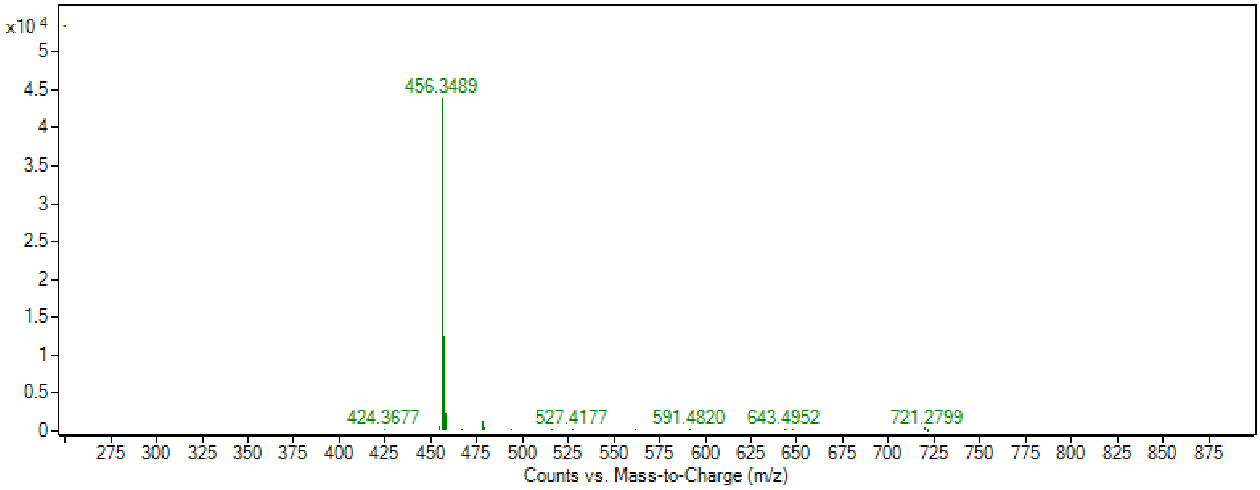

Figure S68. Mass spectrum of compound **25**.

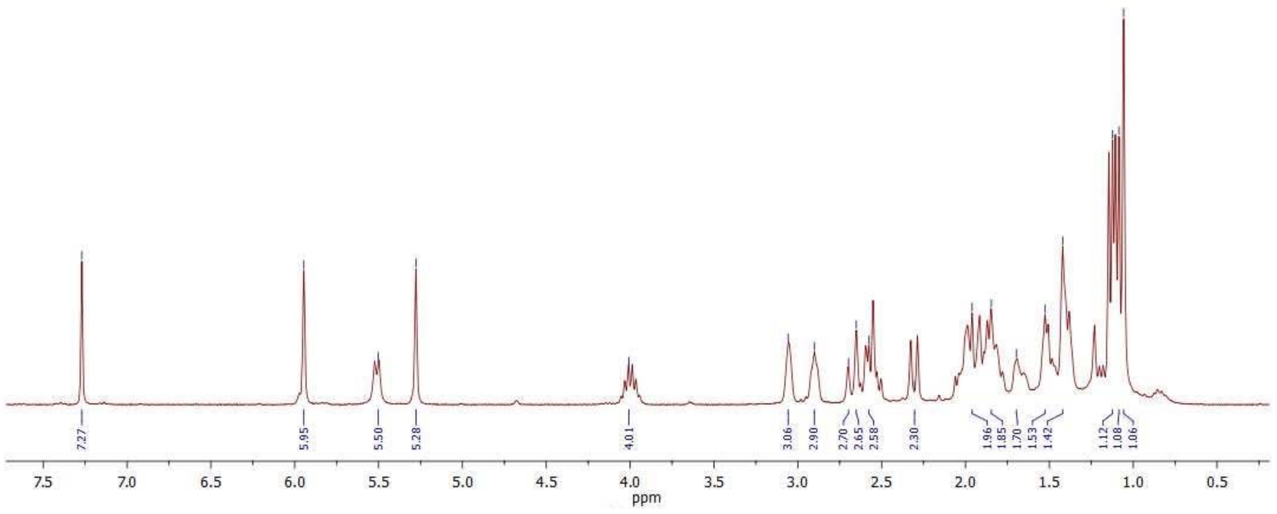

Figure S69. ^{1}H-NMR spectrum of compound **26**.

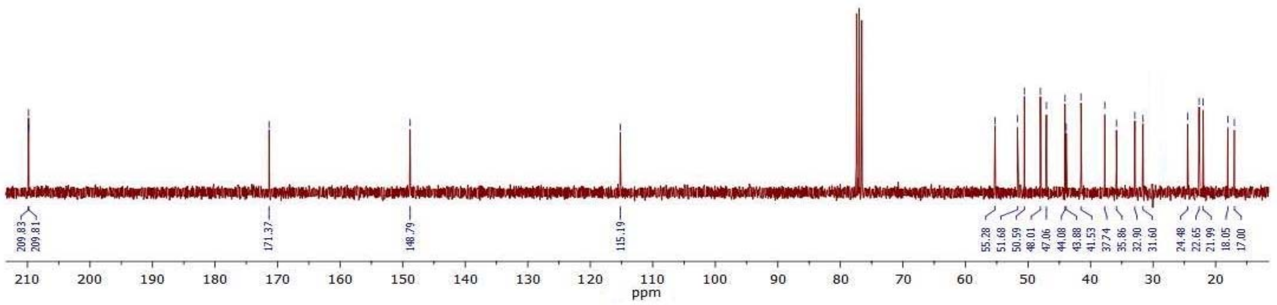

Figure S70. ^{13}C-NMR spectrum of compound **26**.

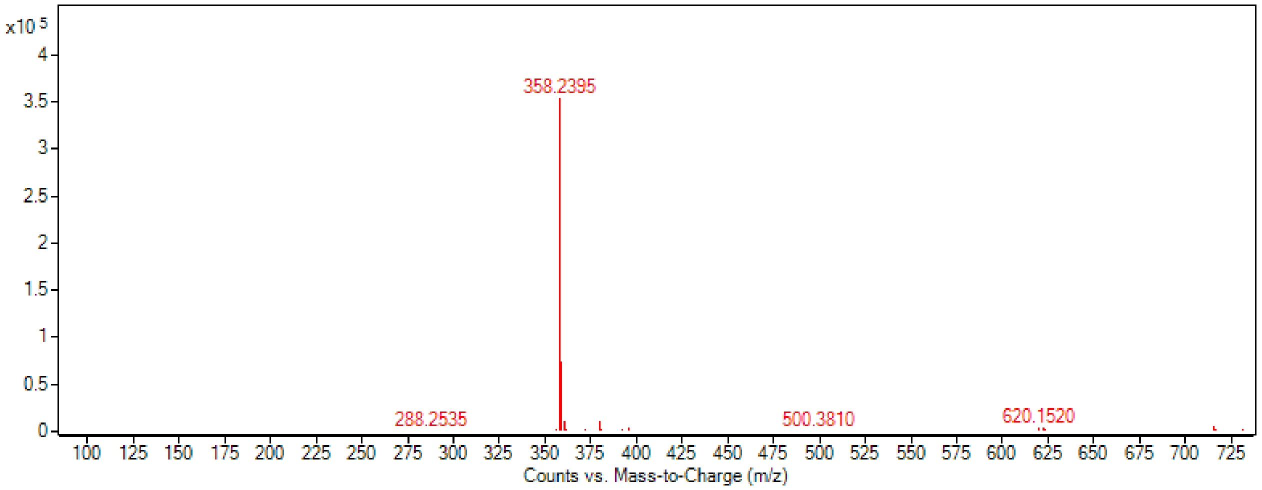

Figure S71. Mass spectrum of compound **26**.

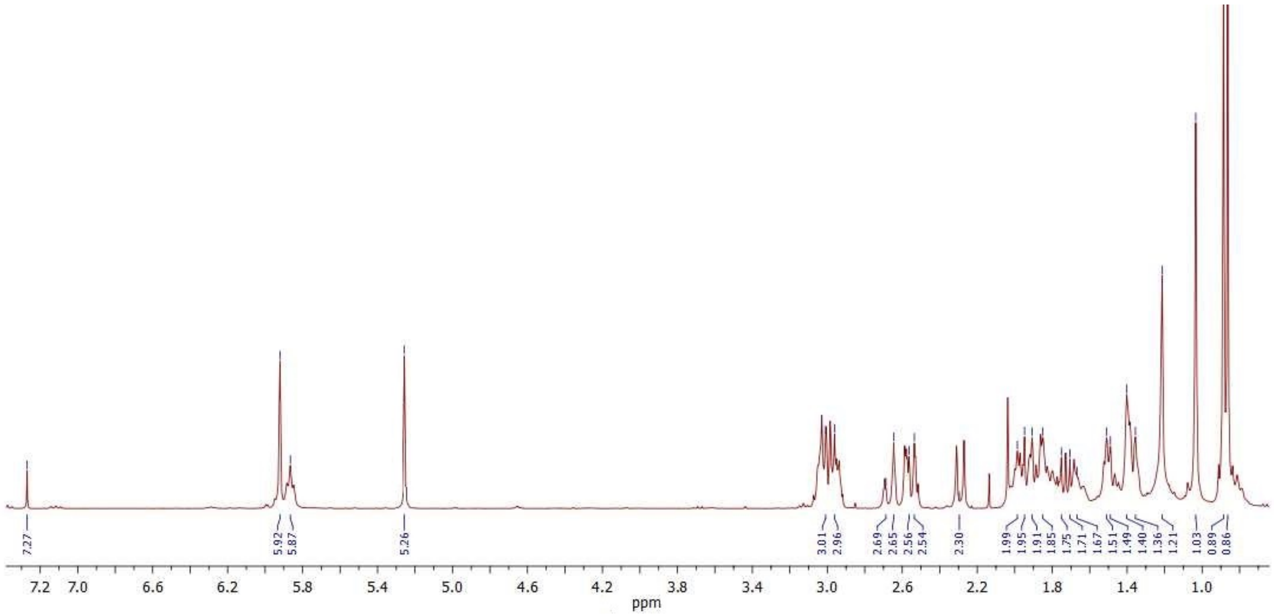

Figure S72. ^{1}H-NMR spectrum of compound **27**.

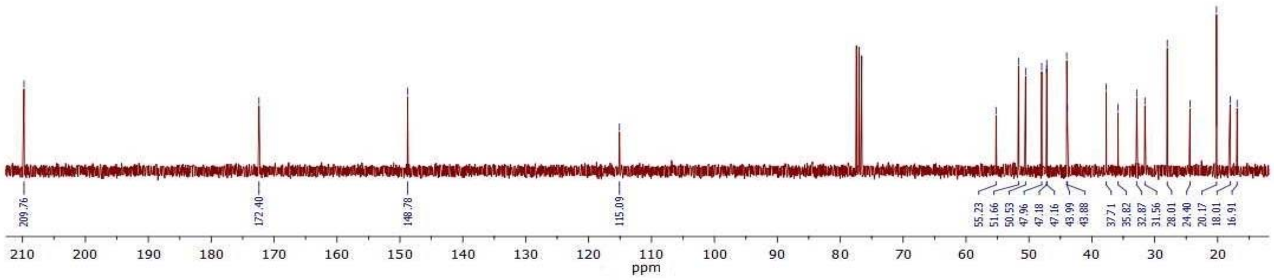

Figure S73. ^{13}C-NMR spectrum of compound **27**.

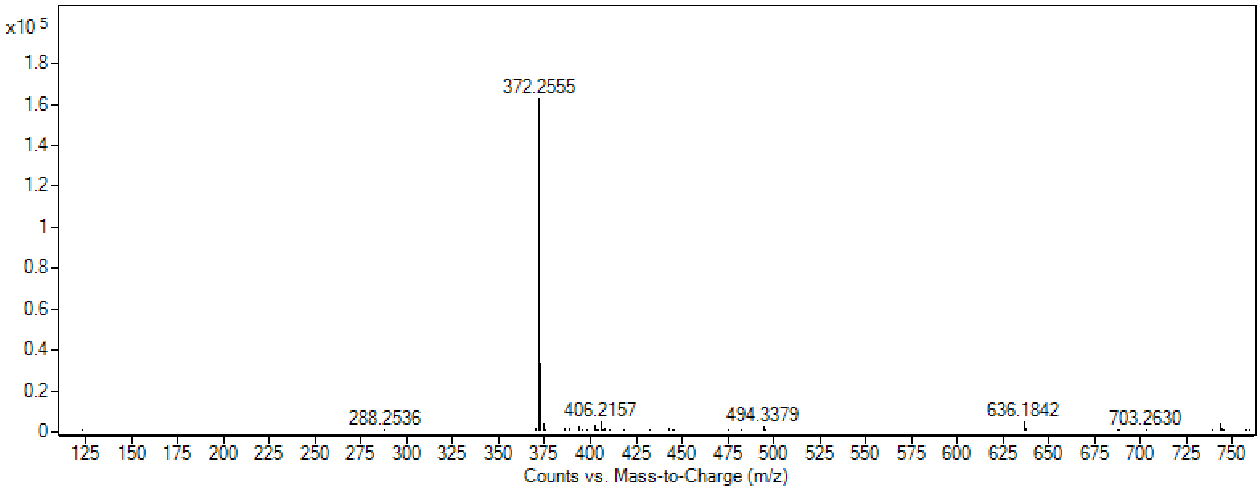

Figure S74. Mass spectrum of compound **27**.

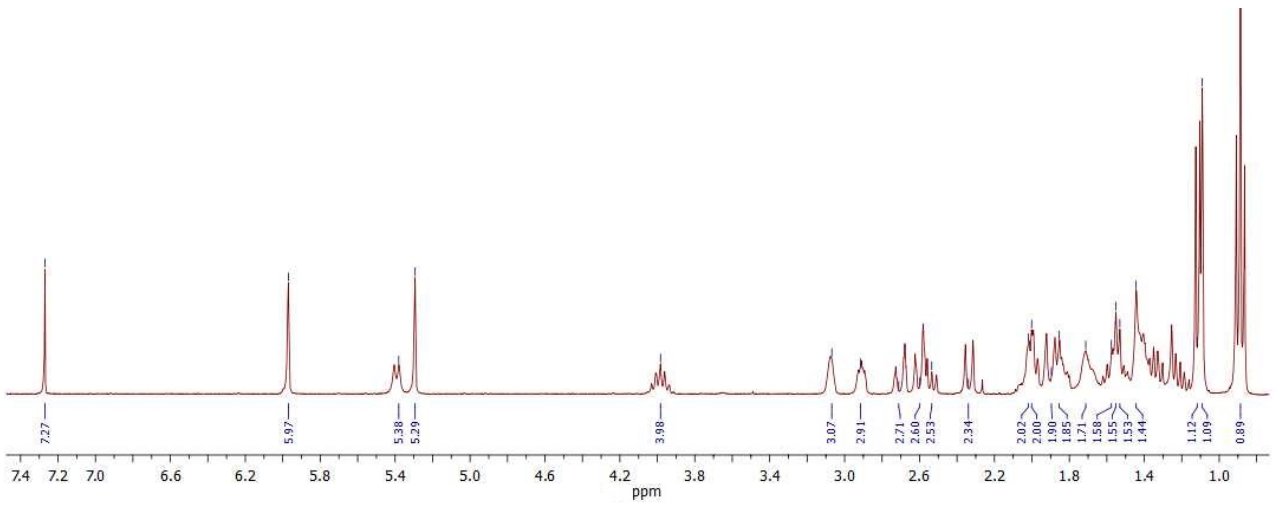

Figure S75. ^{1}H-NMR spectrum of compound **28**.

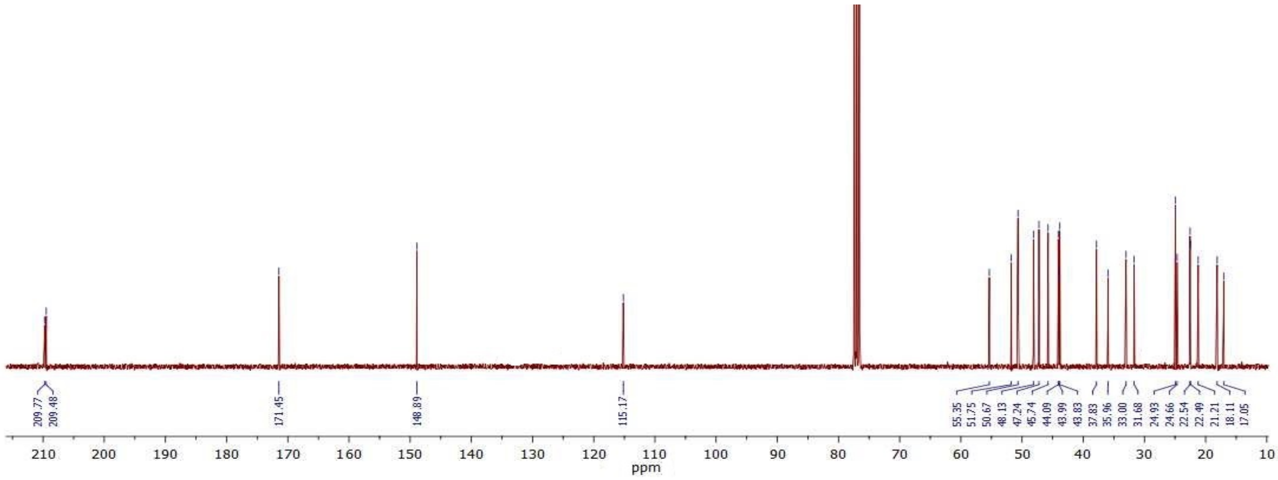

Figure S76. ^{13}C-NMR spectrum of compound **28**.

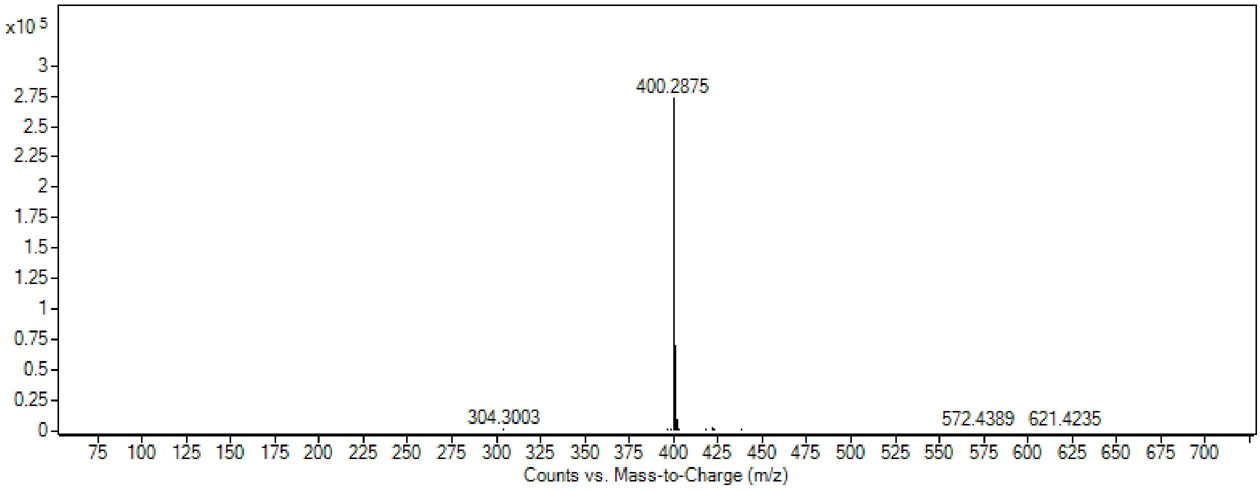

Figure S77. Mass spectrum of compound **28**.

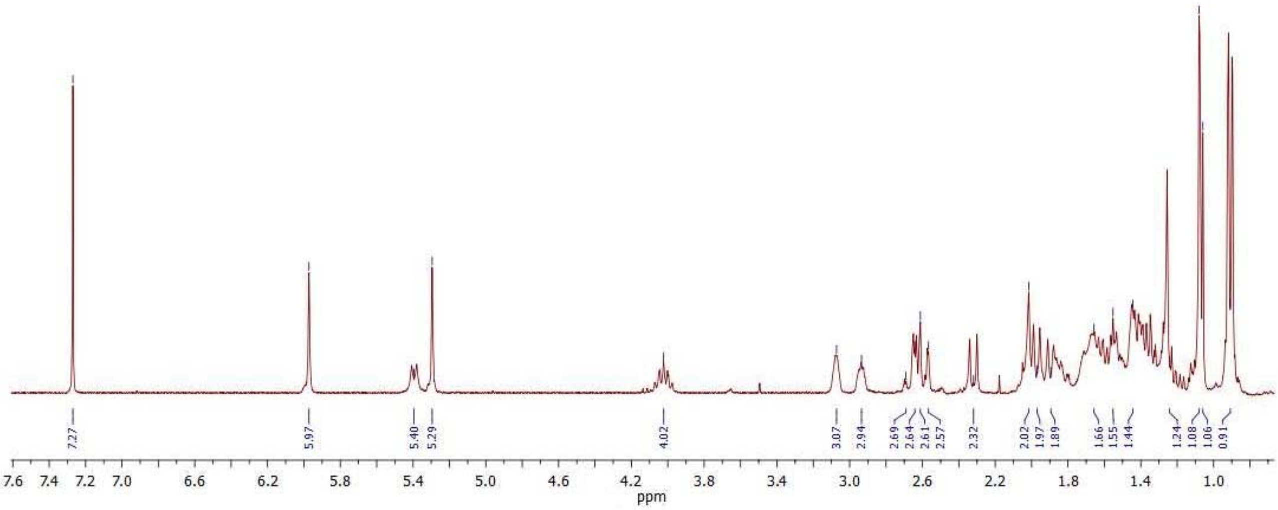

Figure S78. ^{1}H-NMR spectrum of compound **29**.

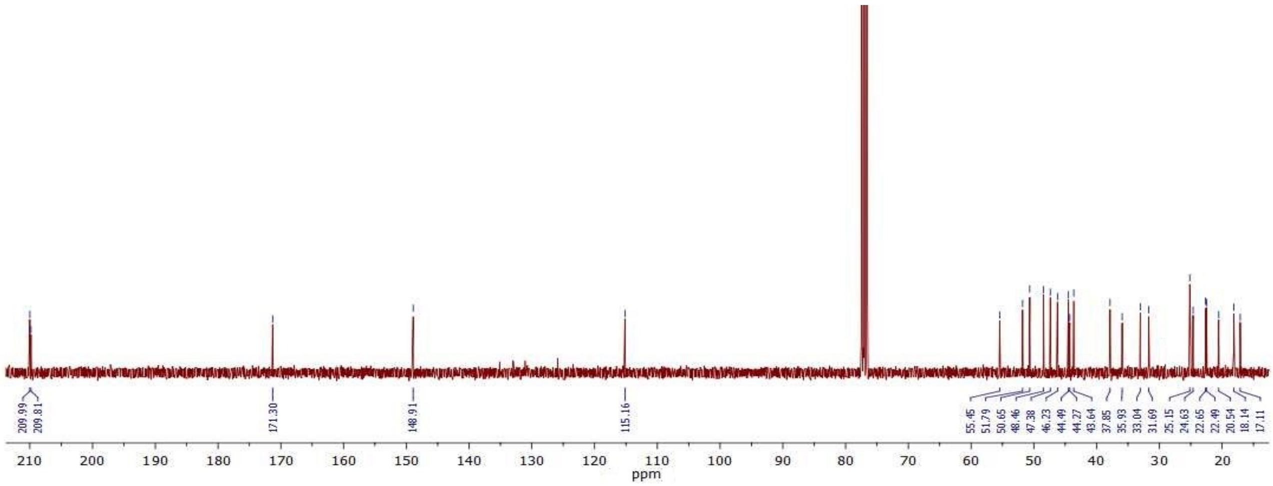

Figure S79. ^{13}C-NMR spectrum of compound **29**.

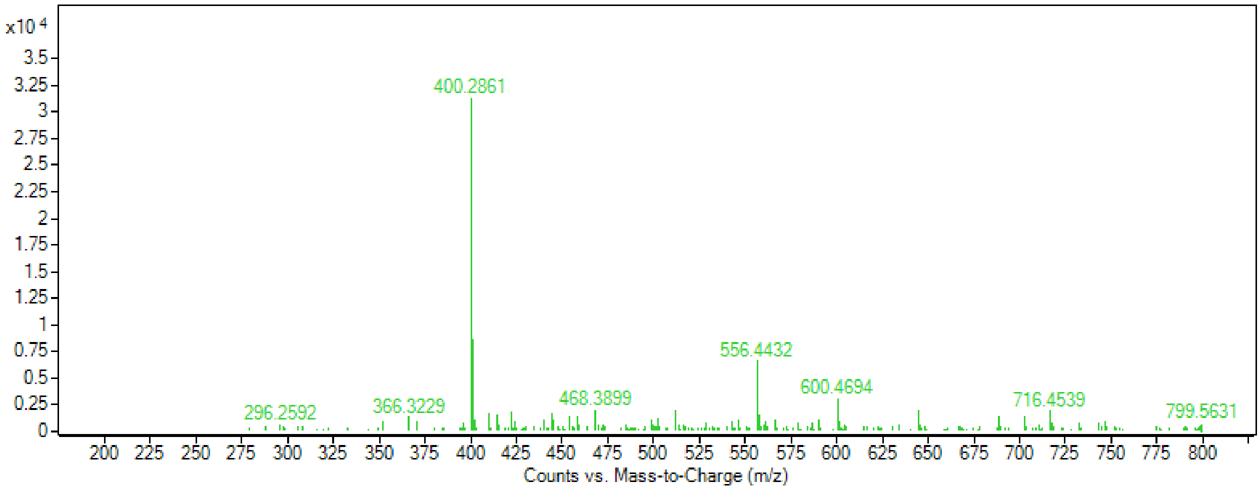

Figure S80. Mass spectrum of compound **29**.

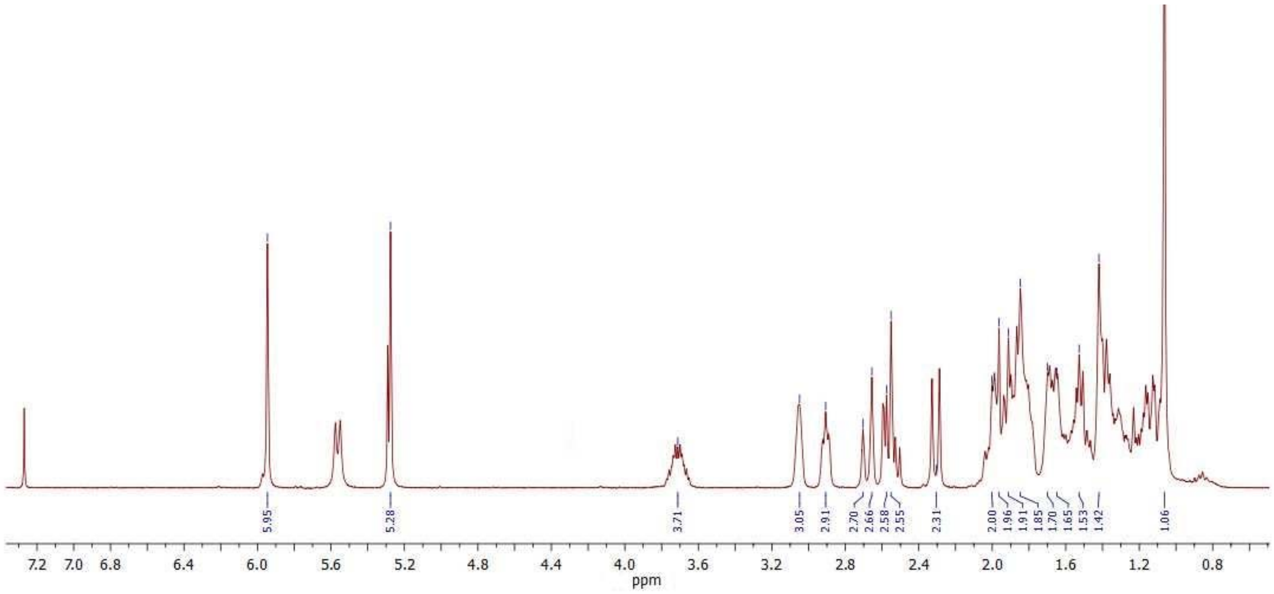

Figure S81. ^{1}H-NMR spectrum of compound **30**.

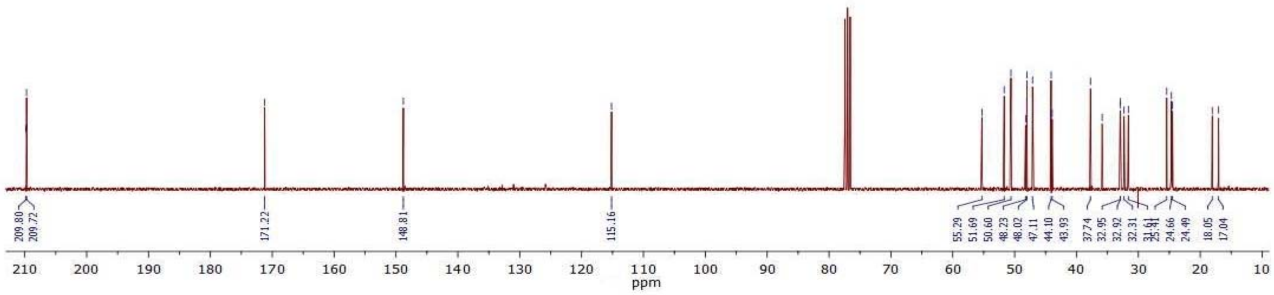

Figure S82. ^{13}C-NMR spectrum of compound **30**.

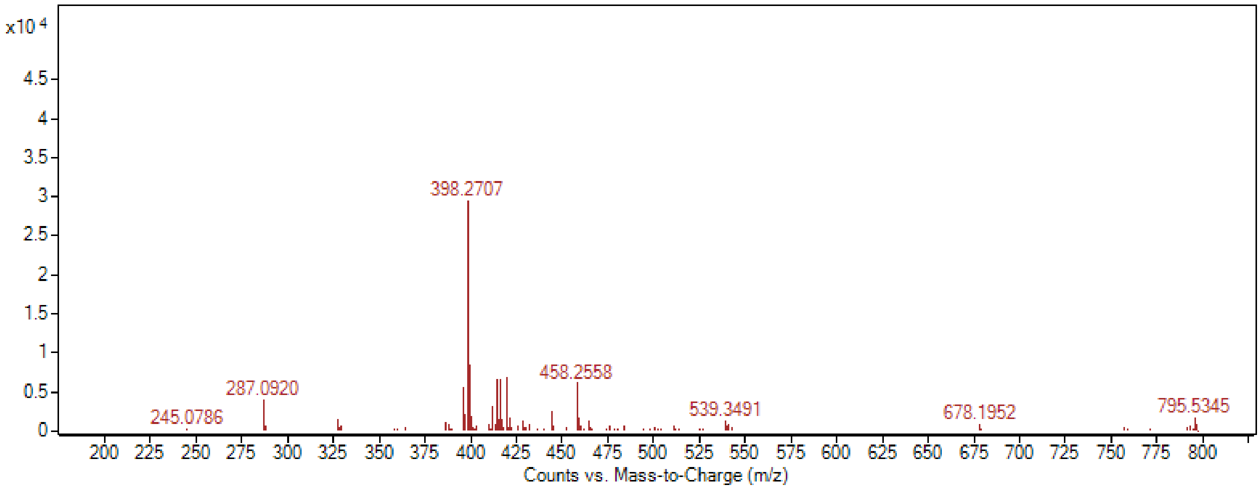

Figure S83. Mass spectrum of compound **30**.

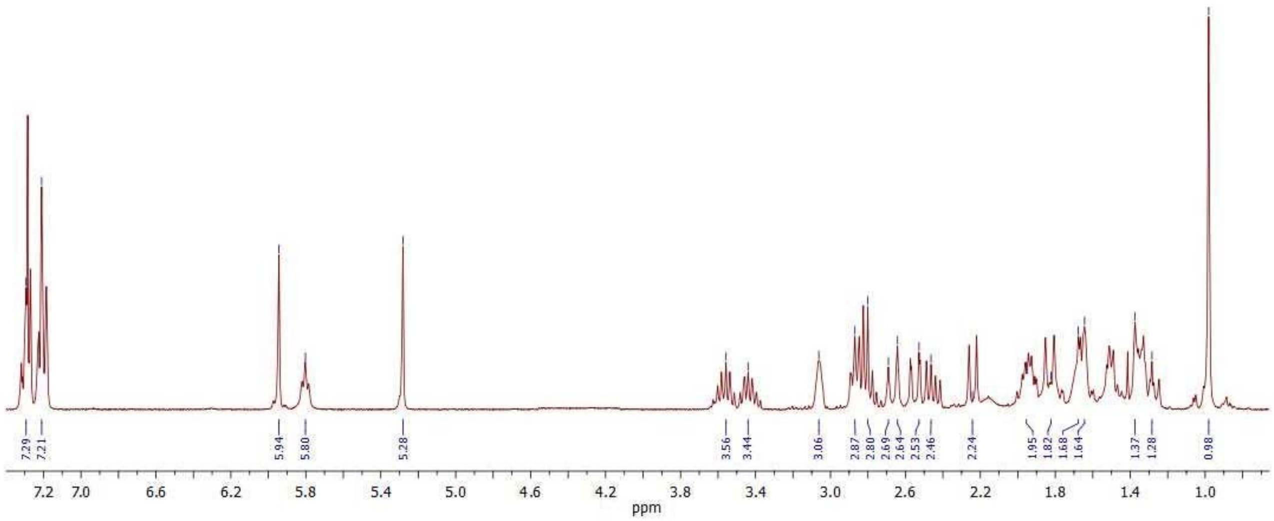

Figure S84. ^{1}H-NMR spectrum of compound **31**.

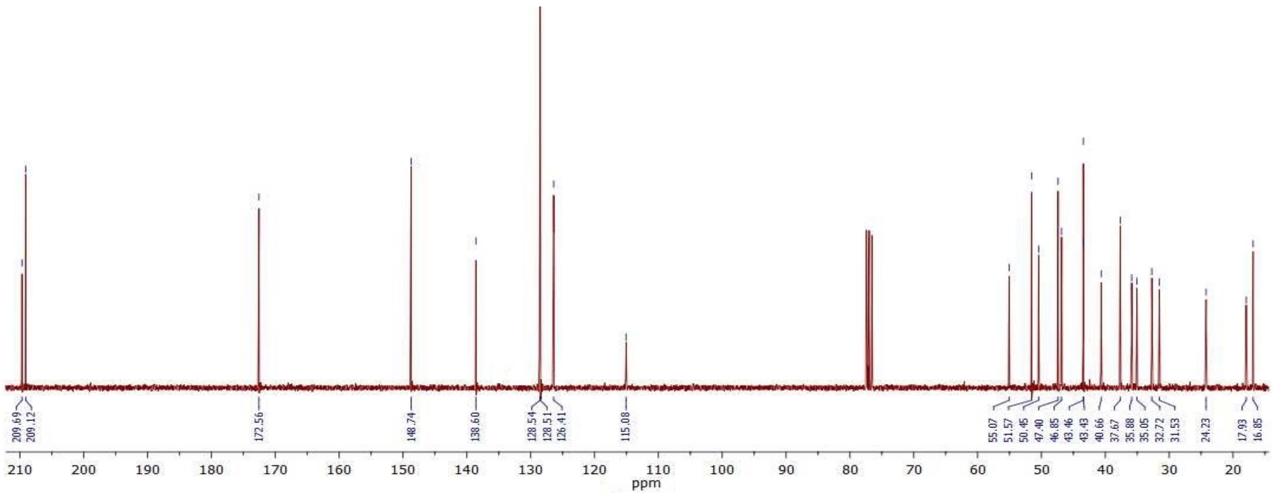

Figure S85. ^{13}C-NMR spectrum of compound **31**.

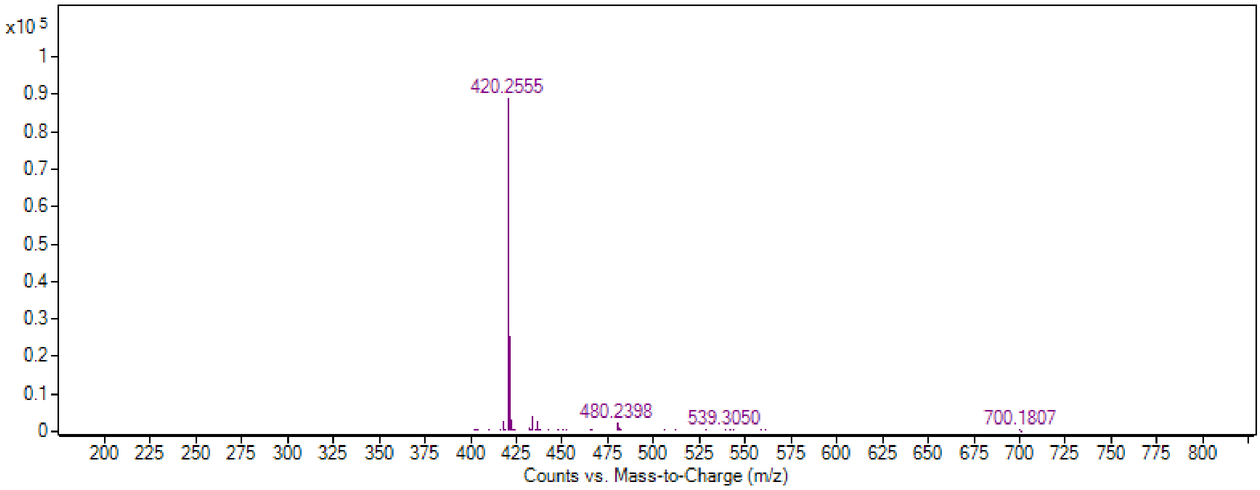

Figure S86. Mass spectrum of compound **31**.

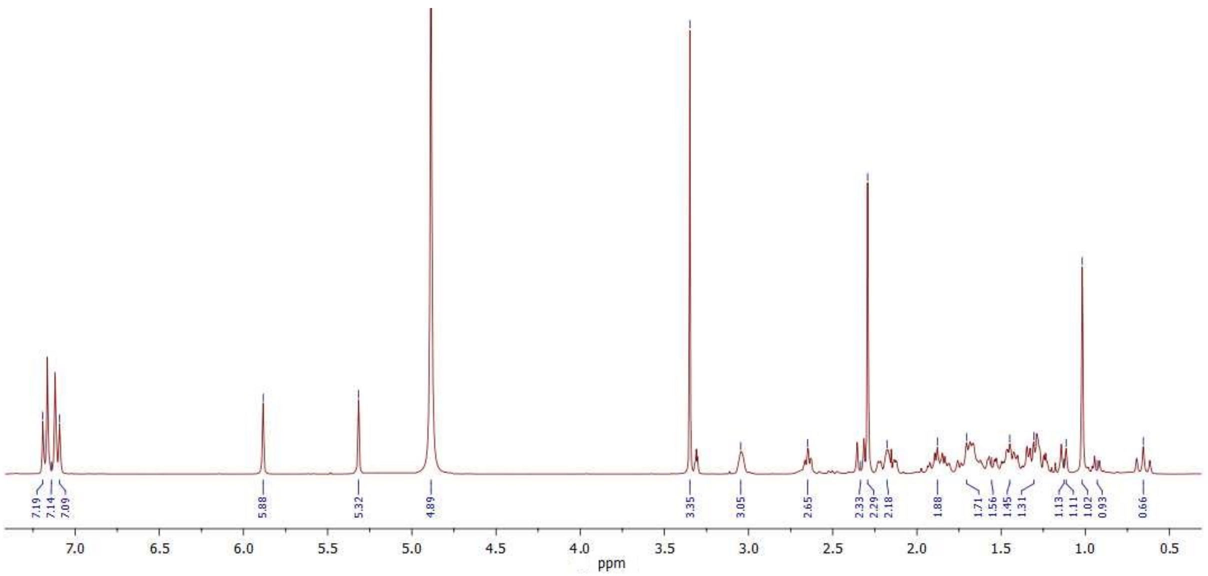

Figure S87. ^{1}H-NMR spectrum of compound **32**.

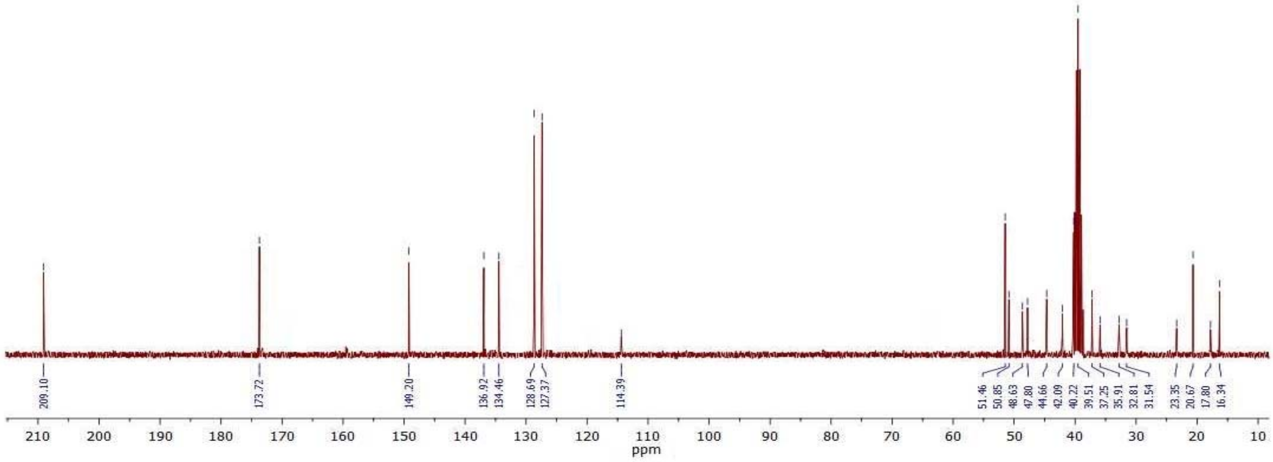

Figure S88. ^{13}C-NMR spectrum of compound **32**.

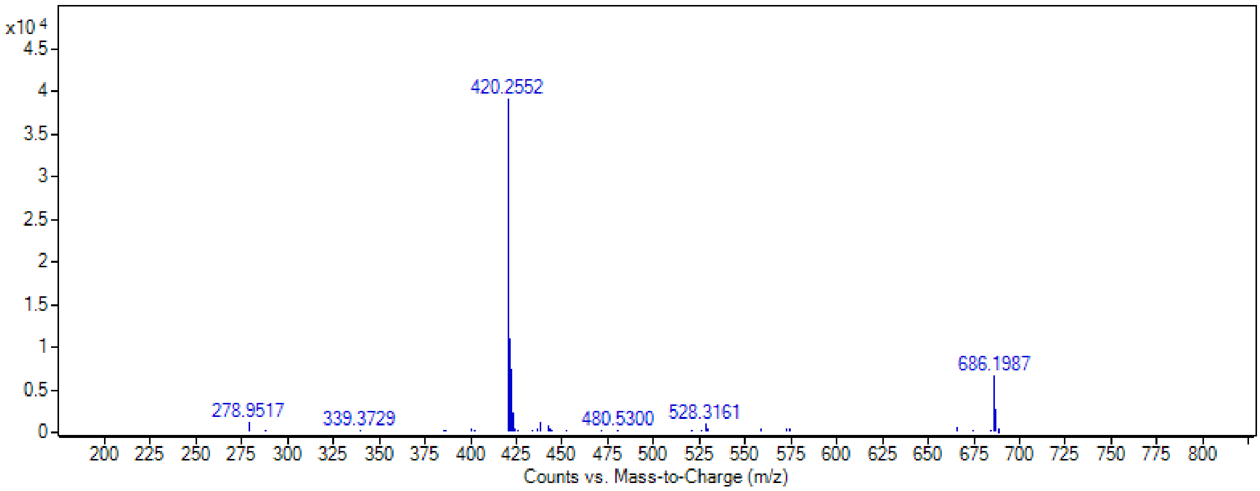

Figure S89. Mass spectrum of compound **32**.
